# Skin Mucus of Gilthead Sea Bream (*Sparus aurata* L.). Protein Mapping and Regulation in Chronically Stressed Fish

**DOI:** 10.3389/fphys.2017.00034

**Published:** 2017-02-01

**Authors:** Jaume Pérez-Sánchez, Genciana Terova, Paula Simó-Mirabet, Simona Rimoldi, Ole Folkedal, Josep A. Calduch-Giner, Rolf E. Olsen, Ariadna Sitjà-Bobadilla

**Affiliations:** ^1^Nutrigenomics and Fish Growth Endocrinology Group, Biology, Culture and Pathology of Marine Species, Institute of Aquaculture Torre de la Sal (IATS-CSIC)Castellón, Spain; ^2^Department of Biotechnology and Life Sciences, University of InsubriaVarese, Italy; ^3^Inter-University Centre for Research in Protein Biotechnologies “The Protein Factory” Polytechnic University of Milan and University of InsubriaVarese, Italy; ^4^Institute of Marine Research MatreMatredal, Norway; ^5^Department of Biology, Norwegian University for Science and TechnologyTrondheim, Norway; ^6^Fish Pathology Group Group, Biology, Culture and Pathology of Marine Species, Institute of Aquaculture Torre de la Sal (IATS-CSIC)Castellón, Spain

**Keywords:** chronic stress, cytokeratins, gilthead sea bream, proteome, skin mucus

## Abstract

The skin mucus of gilthead sea bream was mapped by one-dimensional gel electrophoresis followed by liquid chromatography coupled to high resolution mass spectrometry using a quadrupole time-of-flight mass analyzer. More than 2,000 proteins were identified with a protein score filter of 30. The identified proteins were represented in 418 canonical pathways of the Ingenuity Pathway software. After filtering by canonical pathway overlapping, the retained proteins were clustered in three groups. The mitochondrial cluster contained 59 proteins related to oxidative phosphorylation and mitochondrial dysfunction. The second cluster contained 79 proteins related to antigen presentation and protein ubiquitination pathways. The third cluster contained 257 proteins where proteins related to protein synthesis, cellular assembly, and epithelial integrity were over-represented. The latter group also included acute phase response signaling. In parallel, two-dimensional gel electrophoresis methodology identified six proteins spots of different protein abundance when comparing unstressed fish with chronically stressed fish in an experimental model that mimicked daily farming activities. The major changes were associated with a higher abundance of cytokeratin 8 in the skin mucus proteome of stressed fish, which was confirmed by immunoblotting. Thus, the increased abundance of markers of skin epithelial turnover results in a promising indicator of chronic stress in fish.

## Introduction

A keratinized multi-sheet cellular layer (stratum corneum) covers the epidermis of amphibian adults, reptiles, birds and mammals, whereas skin mucus constitutes the outermost epidermal barrier in fish and aquatic amphibian larvae (Schempp et al., 2009). Cutaneous or skin mucus is thus considered a metabolically active tissue with important roles in respiration, ionic and osmotic regulation, excretion, locomotion, communication, sensory perception, thermal regulation and immunological defense (Negus, 1963; Shephard, 1994; Cone, 2009; Esteban, 2012). Several cell types are involved in regulating the composition of the skin mucus layer, although it is mainly shaped by Goblet cells that release mucous granules containing high molecular weight glycoproteins called mucins (Dharmani et al., 2009). These O-glycosylated glycoproteins are present on the apex of all wet-surfaced epithelia with a well-defined expression pattern, which can be disrupted in response to a wide range of injuries or challenges. For instance, recent experiments in gilthead sea bream (*Sparus aurata* L.) indicate that the gene expression pattern of gut mucins is altered by dietary oils and parasitic enteritis (Pérez-Sánchez et al., 2013b). In addition to glycoproteins, glycosaminoglycans, immunoglobulins, lectins, pheromones, and proteolytic enzymes have been identified in the mucus of different fish species (Fletcher and Grant, 1969; Hjelmeland et al., 1983; van de Winkel et al., 1986; Shiomi et al., 1988; Shephard, 1994; Subramanian et al., 2008; Guardiola et al., 2014; Ren et al., 2015). Most of these molecules are involved in fish innate immunity and skin mucus is considered a key component of fish immune responses (Ellis, 2001; Salinas et al., 2011; Esteban, 2012). This is certainly the result of the evolutionary adaptation of fish to survive in a variety of aquatic environments which are rich in pathogenic organisms. However, immune response can be depleted by stressful conditions, such as those resulting from high density or inappropriate aquaculture husbandry. Thus, limiting stress is now considered a key issue to reduce the economic losses due to opportunistic pathogens in intensive fish farming (Mancuso, 2012).

In teleost fish, stress activates the hypothalamus-pituitary-interrenal axis, leading to a rapid release of the glucocorticoid hormone cortisol by the interrenal tissue, the tissue analogous to the adrenal cortex of mammals (Pottinger, 2008; Pankhurst, 2011). Thus, high circulating levels of cortisol are commonly used as indicators of fish acute stress, though there is no consensus on the endocrine profile for chronically stressed animals or how to assess it without invoking further stress (Pankhurst, 2011; Dickens and Romero, 2013). This notion applies to gilthead sea bream exposed to chronic and acute stress (Arends et al., 1999; Rotllant et al., 2000; Calduch-Giner et al., 2010; Fanouraki et al., 2011), even in a higher manner when intermittent and repetitive stressors are considered (Tort et al., 2001; Ibarz et al., 2007). Hence, expression profiling of stress-responsive genes in different target tissues is envisaged as a complementary tool for assessing nutritional and environmental stress in fish (Terova et al., 2005, 2009; Rimoldi et al., 2012, 2016; Montero et al., 2015a,b), and gilthead sea bream in particular (Pérez-Sánchez et al., 2013a; Benedito-Palos et al., 2014; Bermejo-Nogales et al., 2014). However, this type of approach often requires sacrificing specimens, and the use of a biological sample collected in a minimally invasive manner is more advisable. Skin mucus fulfills such specifications, especially taking into account that one of the most apparent responses of fish to stress is the production of a copious amount of skin mucus (Vatsos et al., 2010). Thus, stress associated with live transport increased the production of sulfated and sialyated skin mucins in channel catfish (Tacchi et al., 2015). Ai-Jun et al. (2013) identified lectins and cytokeratins of skin mucus as bioindicators of thermal stress in turbot. Sea lice infestation increased the abundance of lectins in the skin mucus of Atlantic salmon (Easy and Ross, 2009), while transcriptional and proteomic approaches revealed differentially expressed proteins in the skin mucus of Atlantic cod upon natural infection with *Vibrio anguillarum* (Rajan et al., 2013). Likewise, metabolite profiling of fish skin mucus has been successfully applied as a novel approach for the monitoring and surveillance of wild fish health (Ekman et al., 2015; Dzul-Caamal et al., 2016).

Recently, important research efforts have also been invested in mapping the skin mucus proteome of warm-water marine fish, such as gilthead sea bream (Jurado et al., 2015; Sanahuja and Ibarz, 2015; Cordero et al., 2016) and European sea bass (Cordero et al., 2015), which are the two most important species in Mediterranean aquaculture. These studies have made important advances in defining the composition of fish mucus, also highlighting that both probiotics and overcrowding stress induce proteomic changes mostly involved in immune processes. However, so far, very little is known about the effects of other types of stressors that are closely related to daily farming activities, such as people walking alongside tanks, removal of dead fish, and changes in noise and/or light level that potentially provoke a wide variety of stimuli that are difficult to evaluate in a non-invasive and easy manner (Bratland et al., 2010; Nilsson et al., 2012). Thus, the goal of the present study was to gain new insights into the mucus composition of gilthead sea bream, contributing to identify robust and non-invasive biomarkers in a chronic stress model of daily farming activities, which have been previously characterized by means of more conventional stress biomarkers of fish performance at hormonal and liver transcriptional levels (Bermejo-Nogales et al., 2014). To pursue this issue, one-dimensional and two-dimensional proteomic approaches followed by mass spectrometry were combined, taking advantage of a homologous protein database derived from the IATS-CSIC gilthead sea bream transcriptome (Calduch-Giner et al., 2013) for consistent and reliable protein matches.

## Materials and methods

### Animals and mucus collection

Two-year old gilthead sea bream (average body weight of 320 g) coming from the study of Bermejo-Nogales et al. (2014) comprised a control unstressed group (CTRL) and a group of fish exposed to a model of chronic stress that consisted in a fast series of automated stressors (multiple sensorial stressed fish, M-ST): tank shaking, sounds, moving objects into water, water reverse flow and light flashes in random order for 30 min three times a day (9:30 h, 14:30 h, and 18:30 h) for a period of 21 days. At the end of experimental period, eight fish per group were randomly sampled and anesthetized with 100 mg/L MS-222 (Sigma, Saint Louis, MO, USA). Mucus was gently scraped off the normal skin surface of the left side of fish from operculum to tail with sterile microslides, avoiding collection of blood, urine, and feces along with mucus. Skin mucus was then transferred into Eppendorf tubes and immediately frozen at −80°C until analyzed. All procedures were performed wearing gloves to avoid human contaminations and according to the Norwegian National Ethics Board for experimentation with animals (ID No. 4007) and EU legislation (2010/63/EU) on the handling of experimental animals.

### One-dimensional electrophoresis

The protein composition of mucus was first analyzed by one-dimensional electrophoresis (1-DE). Initially, mucus samples from all animals (CTRL and M-ST fish) were pooled, and triplicate samples (54-56 μg) were separated by sodium dodecyl sulfate polyacrylamide gel electrophoresis (SDS-PAGE) using a TGX Any kD precast gel (Bio-Rad, Hercules, CA, USA) run at 200V for 25 min and stained overnight with colloidal Coomassie (Bio-Rad). The gel was then divided into 10 slices (0.65 cm) that were analyzed independently. Proteins in the gel were digested with protein-grade trypsin (Promega, Madison, WI, USA) and concentrated by speed vacuum at a final volume of 12 μL for mass spectrometry.

### Two-dimensional electrophoresis

Individual samples of CTRL and M-ST fish (*n* = 8 for each group) were precipitated by means of the 2-D Clean-Up kit (GE HealthCare Life Sciences, Buckinghamshire, UK), and then solubilized in labeling buffer (7 M urea, 2 M thiourea, 4% w/v CHAPS, 20 mM Tris). The N-hydroxysuccinimide ester dyes Cy2/3/5 were used for minimal labeling following the mixed internal standard methodology of Alban et al. (2003) according to the manufacturer's protocol (GE HealthCare Life Sciences). Briefly, 50 μg of each experimental sample were individually labeled with 400 pmol of either Cy3 or Cy5. In parallel, a mixed internal standard was generated by combining equal amounts of each experimental sample, which were then labeled with 400 pmol of Cy2. Labeling was performed for 60 min on ice in the dark after which the reaction was quenched by adding 10 nM lysine for 10 min.

About 150 μg of protein (incubated in 65 mM DTT and 1% ampholytes) were loaded into Immobiline DryStrips (pH 3-11 NL, 24 cm), rehydrated overnight in 8 M urea, 4% w/v CHAPS, 12 μL/mL DeStreak reagent, 1% ampholytes. After focusing at 32 kVh at 20°C, strips were equilibrated first for 15 min in reducing solution (6 M urea, 50 mM Tris-HCl, 30% v/v glycerol, 2% w/v SDS, 2% w/v DTT) and then in alkylating solution (6 M urea, 50 mM Tris-HCl, 30% v/v glycerol, 2% w/v SDS, 2.5% w/v iodoacetamide) for 15 min. The second dimension (12.5% polyacrylamide, 25 × 21 cm) was run at 20°C at a constant power of 2 W for 60 min followed by 15 W until the bromophenol blue tracking front had run off the end of the gel (6 h). Fluorescence images were obtained on a Typhoon 9,400 scanner (GE HealthCare Life Sciences). Cy2, Cy3, and Cy5 images were scanned at excitation/emission wavelengths of 488/520 nm, 532/580 nm, and 633/670 nm, respectively, at a resolution of 100 μm. Image analysis was performed using DeCyder v.6.5 software (GE HealthCare Life Sciences). Protein spots displaying a statistically significant difference between groups were manually excised from analytical gels and digested with sequencing-grade trypsin prior to mass spectrometry analysis.

### Mass spectrometry

Samples (5 μl) from 1-DE and two-dimensional electrophoresis (2-DE) were analyzed by liquid chromatography coupled to high-resolution mass spectrometry (LC-HRMS) using a quadrupole time-of-flight mass analyzer (qQTOF). Briefly, samples were loaded onto a trap column (NanoLC Column, 3 μ C18-CL, 350 μm × 0.5 mm, Nikkyo Technos Co. Ltd., Tokyo, Japan) desalted with 0.1% TFA at 3 μL/min for 10 min. Peptide mixtures were then loaded onto an analytical column (LC Column, 3 μ C18-CL, 75 μm × 12 cm, Nikkyo Technos Co. Ltd.) equilibrated in 5% acetonitrile and 0.1% formic acid. Separation was carried out with a linear gradient of 5–40% acetonitrile gradient with 0.1% formic acid at a flow rate of 300 nL/min. Peptides were analyzed in a high resolution nanoESI (qQ) TOF mass spectrometer (AB SCIEX TripleTOF 5,600 System, Applied Biosystems/MDS Sciex, Foster City, CA). The (qQ) TOF was operated in information-dependent acquisition mode, in which a 0.25-s TOF MS scan from 350 to 1,250 m/z, was performed, followed by 0.05 s product-ion scans from 100 to 1,500 m/z on the 50 most intensely 2–5 charged ions. The MS proteomics data have been deposited to the ProteomeXchange Consortium via the PRIDE partner repository with the dataset identifiers PXD004115 and PXD004116.

Protein identity was determined using ProteinPilot v4.5 (AB SCIEX, Applied Biosystems/MDS Sciex), which incorporated the Mascot search algorithm (v2.2, Matrix Science, London, UK). ProteinPilot default parameters were used to generate peak list directly from 5600 TripleTOF wiff files. Mascot was used to search the Expasy protein database or the IATS-CSIC gilthead sea bream database (www.nutrigroup-iats.org/seabreamdb) according to the following parameters: trypsin specificity, carbamidomethyl C to fix modification, deamidated (NQ), Gln->pyro-Glu (N-term Q), Glu->pyro-Glu (N-term E), oxidation (M) to variable modification, 75 ppm as peptide mass tolerance and 0.6 Da as fragment mass tolerance. Proteins with a ProteinPilot score higher than 1.3 were identified with a confidence interval ≥95%. Functional analysis of identified proteins was performed by means of the Ingenuity Pathway Analysis (IPA) software (www.ingenuity.com). For each protein in the analysis, the Uniprot accession equivalent for one of the three higher vertebrates model species in IPA (human, rat, or mouse) was searched as previously reported for the transcriptome-encoding proteins of gilthead sea bream (Calduch-Giner et al., 2013).

### Western blot

In order to validate the results of 2-DE analysis, the increased abundance of keratin type II cytoskeletal 8 in M-ST compared to CTRL group was assessed by means of a Western blot analysis using an antibody directed to human cytokeratin 8. Total protein concentration from mucus samples of CTRL and M-ST fish was determined using the Bradford protein assay (Bio-Rad). The quantified protein analyzed remained almost equal in both experimental groups (1 μg/μl) and equal amounts from the two different groups were mixed with 2 × SDS sample buffer (1.5 M Tris, pH 8.8, 0.2% glycerol, 0.4% SDS, 0.1% 2-mercaptoethanol and 0.05% bromophenol blue), heated for 5 min at 50°C and separated by SDS-PAGE. After electrophoresis, proteins were transferred to polyvinylidenedifluoride (PVDF) membranes (Invitrogen, Gaithersburg, MD, USA) at 15 V for 1 h at room temperature. The membranes were then blocked in 5% nonfat dry milk prepared in TBS (20 Mm Tris pH 7.5, 500 mM NaCl) overnight at 4°C. After blocking, membranes were incubated with rabbit anti-human cytokeratin 8 antibody (PA5-29607, Thermo Scientific, Wilgminton, DE, USA) in antibody buffer (0.1% Tween 20, 1% bovine serum albumin), using a 1:2000 dilution of the supplied antibody concentration. The peptide immunogen (252 amino acids in length) of this primary antibody shared 81% identity (93% homology) with the gilthead sea bream sequence of cytokeratin 8. After primary antibody incubation, membranes were washed four times for 10 min each in T-TBS (TBS with 0.1% Tween 20), incubated with HRP-conjugated goat anti-rabbit IgG at 1:9000 dilution in antibody buffer for 2 h at room temperature, and washed four times for 10 min each in T-TBS. Immunodetection was performed using a chemiluminescent system (Western Blotting Luminol Reagent, Santa Cruz Biotechnologies, CA, USA) and the image on the membrane was captured by VersaDoc Imaging system model 5,000.

### Statistical analyses

Quantification of relative protein levels in 2-DE electrophoresis was performed using Decyder v.6.5 software. Statistical significance was assessed using Student's *t*-test (*p* < 0.05) applying the false discovery rate (FDR) to minimize the number of false positive results. Western blot band intensity was quantified using Quantity One 1-D Analysis Software 4.5 (Bio-Rad) and results were compared by means of Student's *t*-test. The significance threshold was set at *p* < 0.05.

## Results and discussion

### Skin mucus proteins in gilthead sea bream

The current study analyzed the skin mucus of gilthead sea bream, combining 1-DE and 2-DE MS-based proteomic approaches. The primary finding was the large number of proteins that were identified by 1-DE followed by LC-HRMS in comparison to previous proteomic studies in this fish species, in which attention was focused on the most abundant proteins with an over-representation of structural and immune-related proteins. Hence, in the first reference proteome map of gilthead sea bream epidermal mucus (Sanahuja and Ibarz, 2015), up to 92 proteins were identified, and the Gene Ontology enrichment process resulted in 12 functional groups of proteins further classified as structural, metabolic and protection-related proteins. Likewise, a limited set of proteins clustered on structural (23), metabolic (25), stress-response proteins (2) and signal transduction (2) were already reported by Jurado et al. (2015). In Atlantic salmon, up to 521 proteins were identified and classified into nine main groups based on their putative biological processes (Provan et al., 2013). In the present study, 1,595 HRMS spectra were identified by comparing the results of the ProteinPilot with the Expasy protein database when the protein score filter was set up at 1.3. However, by using our gilthead sea bream protein database we identified 2,466 spectra with a much higher protein score (≥20). This number was significantly reduced to 2,060 when a protein score filter of 30 was applied (Table S1), but even in this case, the number of identified proteins was relatively high compared to the proteins that compose other mucosal tissues and body fluids in humans (de Souza et al., 2006; Lee et al., 2009; Marimuthu et al., 2011) and other animal models (Sánchez-Juanes et al., 2013; Bennike et al., 2014; Winiarczyk et al., 2015). Certainly, this was favored by the use of a homologous protein database derived from a reference transcriptome with a high coverage of protein-codifying sequences (more than 15,000 unique sequences in Swissprot database), which first increased the consistency of annotation in parallel with the number of protein isoforms or subunits of a given enzyme or protein complex represented in the analyzed samples (e.g., enzyme subunits of the mitochondrial respiratory chain; protein subunits of the eukaryotic translation initiation factor; ribosomal proteins; proteasome subunits, etc.). Alternatively, we cannot exclude differences in fish species regarding turnover of epidermal cells, which might trigger an enhanced flux of proteins from the cutaneous epithelium toward the skin mucus layer as a result of a normal mucus secretion and/or tissue repair and cell desquamation and renewal. This is perhaps more common than initially expected, as the results of our experimental stress model points out.

### Protein characterization and function

**Figure 1:**
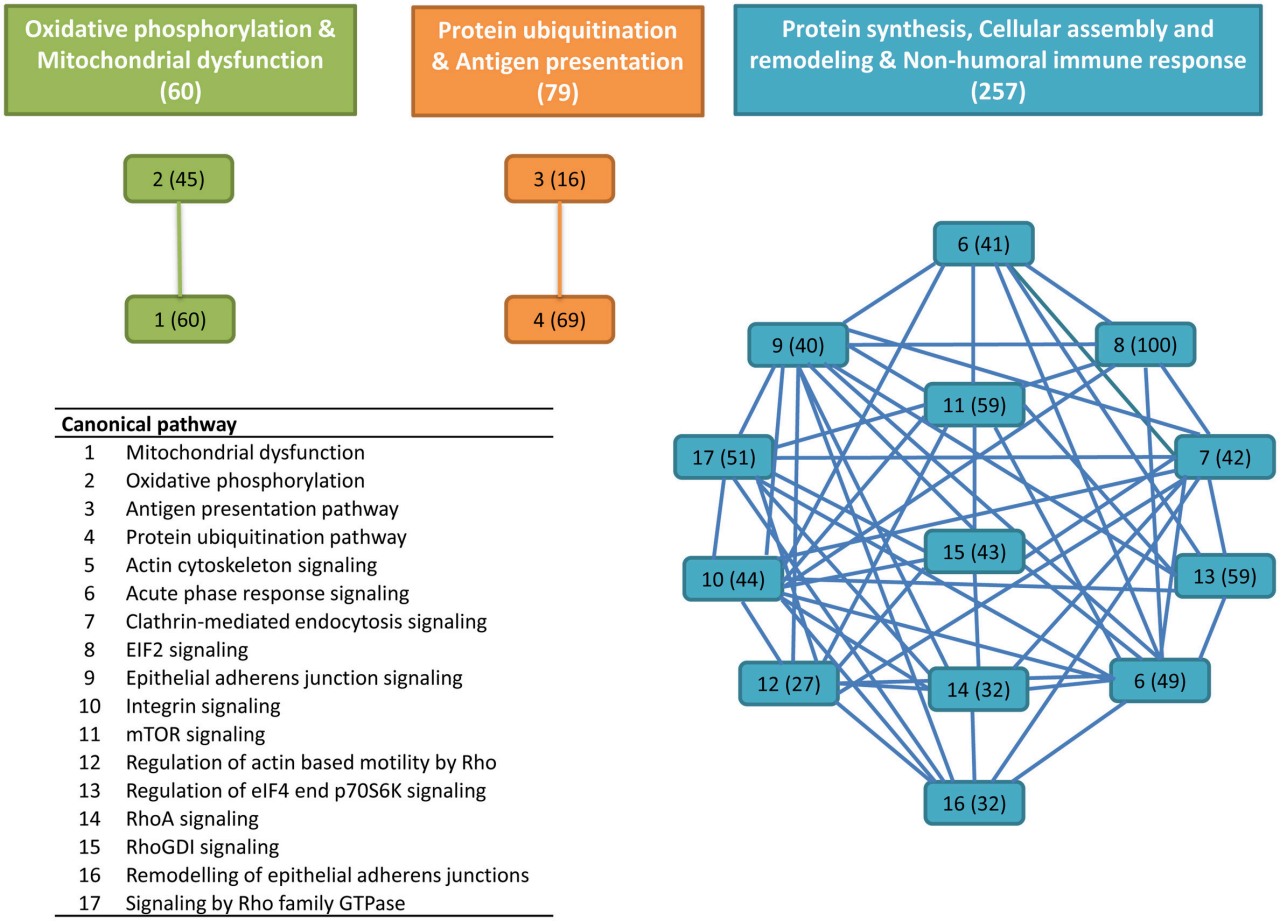
**Overlapping canonical pathway network from gilthead sea bream skin mucus proteins**. This was generated by using Ingenuity Pathway Analysis (IPA) tools. Settings were selected to guarantee a minimum of six common proteins between different canonical pathways. Solid lines show a direct connection between canonical pathways. Numbers assigned to each canonical pathway are represented in the table appended, and numbers in parentheses indicate the number of proteins in each pathway or cluster.

Among the final number of mucus proteins (2,060), more than 89% (1,848 proteins) were eligible for functional pathway analysis using the IPA software. These proteins were represented in 418 canonical pathways out of 644. To easy identify the more relevant pathways and biological processes, an overlapping analysis was performed with a filter of six common proteins among related pathways. From this integrative approach, 17 canonical pathways with significant *p*-values lower than 1E-08 were clustered in three distinct clusters (Figure 1). The first cluster was composed of 60 proteins comprising the canonical pathways “oxidative phosphorylation” and “mitochondrial dysfunction” with a high representation of enzyme subunits of the mitochondrial respiratory chain (NADH dehydrogenase, Complex I; succinate dehydrogenase, Complex II; ubiquinol-cytochrome c reductase, Complex III; cytochrome c oxidase, Complex IV; ATP synthase, Complex V) and mitochondrial cell death and disease factors with both apoptotic (apoptosis-inducing factor 1, caspase 3) and anti-apoptotic (peroxiredoxin 3, PRDX3; peroxiredoxin 5, PRDX5; superoxide dismutase 2, SOD2; Parkinson protein 7, PARK7; nicastrin, NCSTN) roles due to their mediated effects on cell proteolysis, redox sensing, and cell differentiation and proliferation (Table 1).

**Table 1:** **Proteins mapped in the overlapping pathways of oxidative phosphorylation and mitochondrial dysfunction**.

**Protein accession**	**Protein name**	**Protein symbol**	**Canonical pathway(s)**
C2_18809	Aconitase 2, mitochondrial	ACO2	1
C2_6260	Apoptosis-inducing factor 1, mitochondrial	AIFM1	1
C2_1751	ATP synthase subunit alpha, mitochondrial	ATP5A1	1,2
C2_1973	ATP synthase subunit beta, mitochondrial	ATP5B	1,2
C2_18579	ATP synthase subunit gamma, mitochondrial	ATP5C1	1,2
C2_6419	ATP synthase subunit delta, mitochondrial	ATP5D	1,2
C2_24277	ATP synthase subunit epsilon, mitochondrial	ATP5E	1,2
C2_176	ATP synthase subunit b, mitochondrial	ATP5F1	1,2
C2_958	ATP synthase lipid-binding protein, mitochondrial	ATP5G1	1,2
C2_6236	ATP synthase subunit d, mitochondrial	ATP5H	1,2
C2_7051	ATP synthase subunit e, mitochondrial	ATP5I	1,2
C2_1188	ATP synthase subunit f, mitochondrial	ATP5J2	1,2
C2_1627	ATP synthase subunit g, mitochondrial	ATP5L	1,2
C2_123	ATP synthase subunit O, mitochondrial	ATP5O	1,2
C2_3535	Caspase 3	CASP3	1
C2_270	Cytochrome c oxidase subunit 4 isoform 1, mitochondrial	COX4I1	1,2
C2_462	Cytochrome c oxidase subunit 4 isoform 2, mitochondrial	COX4I2	1,2
C2_238	Cytochrome c oxidase subunit 5A, mitochondrial	COX5A	1,2
C2_132	Cytochrome c oxidase subunit 6A, mitochondrial	COX6A1	1,2
C2_1197	Cytochrome c oxidase subunit 6B1	COX6B1	1,2
C2_3512	Cytochrome c oxidase subunit 7B, mitochondrial	COX7B	1,2
C2_4920	Carnitine O-palmitoyltransferase 1, liver isoform	CPT1A	1
C2_568	NADH-cytochrome b5 reductase 3	CYB5R3	1
C2_785	Cytochrome c1, heme protein, mitochondrial	CYC1	1,2
C2_198	Mitochondrial fission 1 protein	FIS1	1
C2_19719	Glutathione reductase, mitochondrial	GSR	1
C2_1416	3-hydroxyacyl-CoA dehydratase 2	HSD17B10	1
C2_106937	ATP synthase subunit a	MT-ATP6	1,2
C2_5715	Cytochrome c oxidase subunit 3	MT-CO3	1,2
C2_5958	Nicastrin	NCSTN	1
C2_8082	NADH dehydrogenase [ubiquinone] 1 alpha subcomplex subunit 1	NDUFA1	1,2
C2_110117	NADH dehydrogenase [ubiquinone] 1 alpha subcomplex subunit 12	NDUFA12	1,2
C2_1985	NADH dehydrogenase [ubiquinone] 1 alpha subcomplex subunit 2	NDUFA2	1,2
C2_3631	NADH dehydrogenase [ubiquinone] 1 alpha subcomplex subunit 4	NDUFA4	1,2
C2_9313	NADH dehydrogenase [ubiquinone] 1 alpha subcomplex subunit 6	NDUFA6	1,2
C2_332	NADH dehydrogenase [ubiquinone] 1 alpha subcomplex subunit 9, mitochondrial	NDUFA9	1,2
C2_11239	Acyl carrier protein, mitochondrial	NDUFAB1	1,2
C2_497	NADH dehydrogenase [ubiquinone] 1 beta subcomplex subunit 10	NDUFB10	1,2
C2_3428	NADH dehydrogenase [ubiquinone] 1 beta subcomplex subunit 4	NDUFB4	1,2
C2_3928	NADH dehydrogenase [ubiquinone] 1 beta subcomplex subunit 6	NDUFB6	1,2
C2_1170	NADH dehydrogenase [ubiquinone] 1 beta subcomplex subunit 7	NDUFB7	1,2
C2_1488	NADH-ubiquinone oxidoreductase 75 kDa subunit, mitochondrial	NDUFS1	1,2
C2_1740	NADH dehydrogenase [ubiquinone] iron-sulfur protein 3, mitochondrial	NDUFS3	1,2
C2_10276	NADH dehydrogenase [ubiquinone] iron-sulfur protein 6, mitochondrial	NDUFS6	1,2
C2_1860	NADH dehydrogenase [ubiquinone] iron-sulfur protein 7, mitochondrial	NDUFS7	1,2
C2_62722	NADH dehydrogenase [ubiquinone] iron-sulfur protein 8, mitochondrial	NDUFS8	1,2
C2_3103	NADH dehydrogenase [ubiquinone] flavoprotein 2, mitochondrial	NDUFV2	1,2
C2_229	Parkinson protein 7	PARK7	1
C2_2292	Pyruvate dehydrogenase E1 component subunit alpha, somatic form, mitochondrial	PDHA1	1
C2_2010	Peroxiredoxin 3	PRDX3	1
C2_4821	Peroxiredoxin 5	PRDX5	1
C2_1571	Succinate dehydrogenase [ubiquinone] flavoprotein subunit, mitochondrial	SDHA	1,2
C2_791	Succinate dehydrogenase [ubiquinone] iron-sulfur subunit, mitochondrial	SDHB	1,2
C2_1642	Superoxide dismutase 2, mitochondrial	SOD2	1
C2_1166	Ubiquinol-cytochrome c reductase, complex III subunit X	UQCR10	1,2
C2_516	Ubiquinol-cytochrome c reductase binding protein	UQCRB	1,2
C2_507	Ubiquinol-cytochrome c reductase, Rieske iron-sulfur polypeptide 1	UQCRFS1	1,2
C2_2118	Ubiquinol-cytochrome c reductase hinge protein	UQCRH	1,2
C2_12420	Ubiquinol-cytochrome c reductase, complex III subunit VII, 9.5kDa	UQCRQ	1,2
C2_318	Voltage-dependent anion-selective channel protein 1	VDAC1	1

*Canonical pathways are indicated by number: (1) Mitochondrial dysfunction; (2) Oxidative phosphorylation*.

As pointed out by Sanahuja and Ibarz (2015), it is still not clear whether the mucus release of glycolytic or mitochondrial enzymes is related to Goblet cell activity or directly to high metabolic activity in the cells of epithelial layers. In any case, increased glycolytic activity has been reported during epidermal infection in Atlantic salmon (Provan et al., 2013) or parental care and mouth-brooding of cichlids (Chong et al., 2006; Iq and Shu-Chien, 2011). Meanwhile, caspase 1 and 6 have been identified in the skin mucus of European sea bass, and it has been suggested that secretion of these cysteine proteases is activated upon danger signals to amplify the inflammatory response (Cordero et al., 2015). The presence of these two caspases was also found in the present study, in addition to a third caspase that was identified as caspase 3. Importantly the caspase 3 cascade is activated by pro-apoptotic mitochondrial molecules such as cytochrome c, and restrained by cellular inhibitors of apoptosis proteins (Srinivasula and Ashwell, 2008). Indeed, elevated levels of caspase 3 in the bloodstream of human patients are considered a symptom of recent myocardial infarction (Agosto et al., 2011). Likewise, PRDXs represent a family of antioxidant proteins with a ubiquitous and differentially regulated abundance in tissues, mucosal surfaces and body fluids (Leyens et al., 2003; Perkins et al., 2015). In the present study, up to four PRDXs (PRDX 1, 4, 5, and 6) were detected in the skin mucus of gilthead sea bream, although only the PRDX5 was represented in the mitochondrial cluster after filtering by canonical pathway overlapping. As reported below, no changes in the abundance of PRDX5 were found in our chronic stress model, although it is noteworthy that this mitochondrial PRDX is highly regulated at the transcriptional level by a wide range of nutritional and environmental stressors (dietary oils, high rearing density and parasitic infections) in the head kidney of gilthead sea bream (Pérez-Sánchez et al., 2011). Additionally, PARK7 is a redox-sensitive chaperone, acting as a sensor of oxidative stress that apparently protects neurons against oxidative stress and cell death, and defects in this gene are the cause of autosomal recessive early-onset Parkinson disease 7 (Bonifati et al., 2003). The presence of this protein in the skin mucus of gilthead sea bream could be viewed, therefore, as part of the antioxidant defense system role of epithelial layers. In this regard, NCSTN might represent another important protein, because in humans it plays a pivotal role in chronic inflammatory skin disease, affecting keratinocyte proliferation, cell-cycle control, and apoptosis (Xiao et al., 2016).

The second node of interconnected skin proteins was composed of 79 proteins involved in protein ubiquitination and antigen presentation pathways with a high representation of major histocompatibility complex, proteasome subunits, ubiquitin enzymes and molecular chaperones, including calnexin, calreticulin and heat shock proteins representative of the six major HSP families based on molecular mass (small HSPs, HSP40, HSP60, HSP70, HSP90 and HSP100) with either cytoplasmic, nuclear plasma membrane or extracellular locations (Table 2). This agrees with the observations made in a previous proteomic gilthead sea bream study, in which more than 1,300 spots were recorded in the skin mucus, but the 100 most abundant were among others ubiquitin/proteasome-related proteins and HSPs (Sanahuja and Ibarz, 2015). Furthermore, in Atlantic cod, changes in proteasome proteins abundance have been reported in response to *V. anguillarum* infection (Rajan et al., 2013) and to challenges with formalin-killed *Aeromonas salmonicida* (Bricknell et al., 2006). Another protein of interest in this cluster was the beta-2-microglobulin, which is now emerging as a consistent marker of immune system activation (Li et al., 2016). This small membrane protein is associated with the heavy chains of class I major histocompatibility complex proteins and serum concentrations are elevated in humans during chronic inflammation, liver disease, renal dysfunction, some acute viral infections, and a number of malignancies associated with the B-lymphocyte lineage (Drüeke and Massy, 2009; Shi et al., 2009). However, to our knowledge no previous reports have addressed the presence and regulation of beta-2-microglobulin in the skin mucus of fish.

**Table 2:** **Proteins mapped in the overlapping pathways of protein ubiquitination and antigen presentation**.

**Protein accession**	**Protein name**	**Protein symbol**	**Canonical pathway(s)**
C2_3233	E3 ubiquitin-protein ligase AMFR	AMFR	4
C2_17008	Anaphase-promoting complex subunit 11	ANAPC11	4
C2_9282	Anaphase-promoting complex subunit 4	ANAPC4	4
C2_6	Beta-2-microglobulin	B2M	3,4
C2_1023	Calreticulin	CALR	3
C2_19770	Calnexin	CANX	3
C2_213	DnaJ homolog subfamily A member 1	DNAJA1	4
C2_5322	DnaJ homolog subfamily C member 17	DNAJC17	4
C2_8665	DnaJ homolog subfamily C member 22	DNAJC22	4
C2_121377	HLA class II histocompatibility antigen, DP beta 1 chain	HLA-DPB1	3
C2_104432	H-2 class II histocompatibility antigen, A-R alpha chain	HLA-DQA1	3
C2_728	DLA class II histocompatibility antigen, DR-1 beta chain	HLA-DR1	3
C2_105193	H-2 class II histocompatibility antigen, E-D alpha chain	HLA-DRA	3
C2_113147	HLA class II histocompatibility antigen, DRB1-4 beta chain	HLA-DRB4	3
C2_4132	Heat shock protein HSP 90-alpha 1	HSP90AA1	4
C2_42	Heat shock protein HSP 90-beta	HSP90AB1	4
C2_1490	Endoplasmin (GRP-94)	HSP90B1	4
C2_6720	Heat shock 70 kDa protein 4	HSPA4	4
C2_25027	78 kDa glucose-regulated protein	HSPA5	4
C2_4763	Heat shock cognate 71 kDa protein	HSPA8	4
C2_82883	Stress-70 protein, mitochondrial	HSPA9	4
C2_10046	Heat shock protein beta-11	HSPB11	4
C2_5222	60 kDa heat shock protein, mitochondrial	HSPD1	4
C2_4023	10 kDa heat shock protein, mitochondrial	HSPE1	4
C2_2116	Heat shock protein 105 kDa	HSPH1	4
C2_121640	Major histocompatibility complex class I-related gene protein	MR1	3
C2_251	Protein disulfide-isomerase A3	PDIA3	3
C2_276	Proteasome subunit alpha type-1	PSMA1	4
C2_39253	Proteasome subunit alpha type-2	PSMA2	4
C2_667	Proteasome subunit alpha type-3	PSMA3	4
C2_89	Proteasome subunit alpha type-5	PSMA5	4
C2_979	Proteasome subunit alpha type-6	PSMA6	4
C2_486	Proteasome subunit alpha type-7	PSMA7	4
C2_303	Proteasome subunit beta type-1-B	PSMB1	4
C2_53426	Proteasome subunit beta type-10	PSMB10	4
C2_4220	Proteasome subunit beta type-2	PSMB2	4
C2_1113	Proteasome subunit beta type-3	PSMB3	4
C2_1989	Proteasome subunit beta type-4 (Fragment)	PSMB4	4
C2_2719	Proteasome subunit beta type-5	PSMB5	3,4
C2_104936	Proteasome subunit beta type-6-B like protein	PSMB6	3,4
C2_4274	Proteasome subunit beta type-9	PSMB9	3,4
C2_4264	26S protease regulatory subunit 4	PSMC1	4
C2_3002	26S protease regulatory subunit 7	PSMC2	4
C2_1666	26S protease regulatory subunit 6A	PSMC3	4
C2_482	26S protease regulatory subunit 6B	PSMC4	4
C2_514	26S protease regulatory subunit 8	PSMC5	4
C2_1520	26S protease regulatory subunit 10B	PSMC6	4
C2_2728	26S proteasome non-ATPase regulatory subunit 1	PSMD1	4
C2_3102	26S proteasome non-ATPase regulatory subunit 11	PSMD11	4
C2_1392	26S proteasome non-ATPase regulatory subunit 12	PSMD12	4
C2_790	26S proteasome non-ATPase regulatory subunit 13	PSMD13	4
C2_807	26S proteasome non-ATPase regulatory subunit 14	PSMD14	4
C2_4556	26S proteasome non-ATPase regulatory subunit 2	PSMD2	4
C2_1006	26S proteasome non-ATPase regulatory subunit 3	PSMD3	4
C2_8032	26S proteasome non-ATPase regulatory subunit 6	PSMD6	4
C2_364	26S proteasome non-ATPase regulatory subunit 7	PSMD7	4
C2_1843	26S proteasome non-ATPase regulatory subunit 8	PSMD8	4
C2_19056	Proteasome activator complex subunit 1	PSME1	4
C2_52053	Proteasome activator complex subunit 2	PSME2	4
C2_159	S-phase kinase-associated protein 1	SKP1	4
C2_6616	Antigen peptide transporter 1	TAP1	3,4
C2_8891	Antigen peptide transporter 2	TAP2	3,4
C2_27605	Tapasin	TAPBP	3
C2_529	Transcription elongation factor B polypeptide 1	TCEB1	4
C2_558	Transcription elongation factor B polypeptide 2	TCEB2	4
C2_15542	Thimet oligopeptidase	THOP1	4
C2_8231	Ubiquitin-like modifier-activating enzyme 1	UBA1	4
C2_5227	Ubiquitin-conjugating enzyme E2 D2	UBE2D2	4
C2_187	Ubiquitin-conjugating enzyme E2 D3	UBE2D3	4
C2_17030	Ubiquitin-conjugating enzyme E2 N	UBE2N	4
C2_23677	Ubiquitin-conjugating enzyme E2 variant 1C	UBE2V1	4
C2_9398	Ubiquitin-protein ligase E3A	UBE3A	4
C2_5640	Ubiquitin carboxyl-terminal hydrolase isozyme L1	UCHL1	4
C2_660	Ubiquitin carboxyl-terminal hydrolase isozyme L3	UCHL3	4
C2_1964	Ubiquitin carboxyl-terminal hydrolase 14	USP14	4
C2_11890	Ubiquitin carboxyl-terminal hydrolase 22	USP22	4
C2_18121	Ubiquitin carboxyl-terminal hydrolase 37	USP37	4
C2_66335	Ubiquitin carboxyl-terminal hydrolase 8	USP8	4
C2_19855	Probable ubiquitin carboxyl-terminal hydrolase FAF-X	USP9X	4

*Canonical pathways are indicated by number: (3) Antigen presentation pathway; (4) Protein ubiquitination pathway*.

The third cluster was the most populated one with 257 proteins in 13 interconnected canonical pathways (Table 3). Many of them are involved in protein synthesis (EIF2 signaling, mTOR signaling) and the maintenance of epithelial integrity (remodeling of epithelial adherens junctions, regulation of actin-based motility by Rho, epithelial adherens junction signaling, etc.) with also an important representation of proteins of acute phase response signaling. This set of proteins included among others, alpha-2-HS-glycoprotein, alpha-2-macroglobulin, amyloid P component, apolipoprotein A-I, angiotensinogen, ceruloplasmin, complement component 2, 3, 5, and 9, complement factor B, ferritin, fibrinogen, hemopexin, inter-alpha-trypsin inhibitor heavy chain H2 and H3, serpin peptidase inhibitor, transthyretin and transferrin. Most of them have been reported in other proteomic studies of mucosal surfaces, being this finding consistent with a key role of mucosal immunity during the course of most fish infections, probably due to the fact that aquatic environment favors a more intimate contact with pathogens (Salinas et al., 2011; Esteban, 2012). We are still far from fully exploiting this information on a routine basis, but our study will contribute to enlarge the list of immune-relevant proteins that are susceptible to be included in protein arrays or more targeted immune kits.

**Table 3:** **Proteins mapped in the overlapping pathways of protein synthesis, cellular assembly and remodeling and non-humoral immune response**.

**Protein accession**	**Protein name**	**Protein symbol**	**Canonical pathway(s)**
s_flp0005a11_f_1	Alpha-2-macroglobulin	A2M	6
C2_2	Actin, cytoplasmic 1	ACTB	5,7,9,10,12,14,15,16,17
C2_1387	Actin, alpha cardiac	ACTC1	5,7,9,10,12,14,15,16,17
C2_102126	Actin, cytoplasmic 2	ACTG1	5,7,9,10,14,15,16,17
C2_1801	Alpha-actinin-3	ACTN3	5,9,10,16
C2_26557	Alpha-actinin-4	ACTN4	5,9,10,16
C2_3453	Actin-related protein 2-A	ACTR2	5,7,9,10,12,14,15,16,17
C2_1771	Actin-related protein 3	ACTR3	5,7,9,10,12,14,15,16,17
C2_5961	Protein argonaute-2	AGO2	8,13
C2_17965	Angiotensinogen	AGT	6
C2_22548	Alpha-2-HS-glycoprotein	AHSG	6
C2_2100	Protein AMBP	AMBP	6
C2_37278	AP-1 complex subunit beta-1	AP1B1	7
C2_5784	AP-2 complex subunit beta	AP2B1	7
C2_1474	AP-2 complex subunit mu-1-A	AP2M1	7
C2_14608	Serum amyloid P-component	APCS	6
C2_1042	Apolipoprotein A-I	APOA1	6,7
s_rl0001e11_f_1	Apolipoprotein B-100	APOB	7
C2_5591	Apolipoprotein Eb	APOE	7
C2_487	ADP-ribosylation factor 1-like 2	ARF1	10
C2_2038	ADP-ribosylation factor 4	ARF4	10
C2_3418	ADP-ribosylation factor 6	ARF6	7,10,16
C2_1604	Rho GDP-dissociation inhibitor 1	ARHGDIA	12,15
C2_22184	Rho guanine nucleotide exchange factor 5	ARHGEF5	15,17
C2_18222	Actin-related protein 2/3 complex subunit 1A	ARPC1A	5,7,9,10,12,14,15,16,17
C2_4212	Actin-related protein 2/3 complex subunit 1B	ARPC1B	5,7,9,10,12,14,15,16,17
C2_22529	Actin-related protein 2/3 complex subunit 2	ARPC2	5,7,9,10,12,14,15,16,17
C2_4166	Actin-related protein 2/3 complex subunit 3	ARPC3	5,7,9,10,12,14,15,16,17
C2_427	Actin-related protein 2/3 complex subunit 4	ARPC4	5,7,9,10,12,14,15,16,17
C2_503	Actin-related protein 2/3 complex subunit 5	ARPC5	5,7,9,10,12,14,15,16,17
FM156976	Arf-GAP with SH3 domain, ANK repeat and PH domain-containing protein 1	ASAP1	10
FP333165	Complement C2	C2	6
C2_1398	Complement C3	C3	6
s_flp0005d01_f_1	Complement C5	C5	6
s_rl0001d01_f_1	Complement component C9	C9	6
C2_15607	Calpain-1 catalytic subunit	CAPN1	10
C2_55335	Calpain-5	CAPN5	10
C2_44459	Calpain-8	CAPN8	10
C2_5497	Calpain small subunit 1	CAPNS1	10
C2_9606	CD2-associated protein	CD2AP	7
C2_16241	Cdc42 effector protein 2	CDC42EP2	14,17
C2_2045	Cadherin-1	CDH1	9,15,16,17
C2_11775	Cadherin-2	CDH2	9,15,17
C2_9760	Complement factor B	CFB	6
C2_1192	Cofilin-2	CFL2	5,14,15,17
C2_1929	Calcium-binding protein p22	CHP1	7
C2_9111	CAP-Gly domain-containing linker protein 1	CLIP1	9,16,17
C2_39986	Ceruloplasmin	CP	6
C2_540	Casein kinase II subunit alpha	CSNK2A1	7
C2_8203	Casein kinase II subunit beta	CSNK2B	7
C2_13521	Catenin alpha-2	CTNNA2	9,16
C2_23957	Catenin delta-1	CTNND1	9,16
C2_33608	Cytoplasmic FMR1-interacting protein 2	CYFIP2	5
C2_6275	Dynamin-2	DNM2	7,16
C2_25618	Eukaryotic translation initiation factor 1A, X-chromosomal	EIF1AX	8,13
C2_4011	Eukaryotic translation initiation factor 2 subunit 1	EIF2S1	8,13
C2_5966	Eukaryotic translation initiation factor 2 subunit 2	EIF2S2	8,13
C2_1614	Eukaryotic translation initiation factor 2 subunit 3	EIF2S3	8,13
C2_533	Eukaryotic translation initiation factor 3 subunit A	EIF3A	8,11,13
C2_630	Eukaryotic translation initiation factor 3 subunit B	EIF3B	8,11,13
C2_114	Eukaryotic translation initiation factor 3 subunit E	EIF3E	8,11,13
C2_92	Eukaryotic translation initiation factor 3 subunit F	EIF3F	8,11,13
C2_1542	Eukaryotic translation initiation factor 3 subunit G	EIF3G	8,11,13
C2_312	Eukaryotic translation initiation factor 3 subunit I	EIF3I	8,11,13
C2_5501	Eukaryotic translation initiation factor 3 subunit K	EIF3K	8,11,13
C2_372	Eukaryotic translation initiation factor 3 subunit L	EIF3L	8,11,13
C2_68	Eukaryotic translation initiation factor 3 subunit M	EIF3M	8,11,13
C2_406	Eukaryotic initiation factor 4A-I	EIF4A1	8,11,13
C2_746	Eukaryotic initiation factor 4A-II	EIF4A2	8,11,13
C2_1414	Eukaryotic initiation factor 4A-III	EIF4A3	8,11,13
C2_1810	Eukaryotic translation initiation factor 4E	EIF4E	8,11,13
C2_2581	Eukaryotic translation initiation factor 4E-binding protein 2	EIF4EBP2	13
C2_2506	Eukaryotic translation initiation factor 4 gamma 1	EIF4G1	8,11,13
C2_31468	Prothrombin	F2	5,6,7
FP332283	Fibrinogen beta chain	FGB	6
C2_38689	Fibrinogen gamma chain	FGG	6
C2_49308	Formin-binding protein 1 homolog	FNBP1	10,11,12,15,17
C2_105435	Ferritin light chain, oocyte isoform	FTL	6
C2_32560	Rab GDP dissociation inhibitor alpha	GDI1	15
C2_455	Rab GDP dissociation inhibitor beta	GDI2	15
C2_75053	Guanine nucleotide-binding protein subunit alpha-11	GNA11	15,17
C2_5820	Guanine nucleotide-binding protein subunit alpha-13	GNA13	5,14,15,17
C2_3738	Guanine nucleotide-binding protein G(i) subunit alpha-1	GNAI1	15,17
C2_6805	Guanine nucleotide-binding protein G(k) subunit alpha	GNAI3	15,17
C2_35672	Guanine nucleotide-binding protein G(t) subunit alpha-2	GNAT2	15,17
C2_8162	Guanine nucleotide-binding protein G(I)/G(S)/G(T) subunit beta-1	GNB1	15,17
C2_41327	Guanine nucleotide-binding protein G(I)/G(S)/G(T) subunit beta-2	GNB2	15,17
C2_45	Guanine nucleotide-binding protein subunit beta-2-like 1	GNB2L1	15,17
C2_3160	Guanine nucleotide-binding protein subunit beta-4	GNB4	15,17
C2_64382	Guanine nucleotide-binding protein G(I)/G(S)/G(O) subunit gamma-12	GNG12	1,15,17
C2_7512	Guanine nucleotide-binding protein G(I)/G(S)/G(O) subunit gamma-2	GNG2	15,17
C2_4717	Growth factor receptor-bound protein 2	GRB2	5,6,7,8,10,13
C2_265	Gelsolin	GSN	5,12
C2_2149	Heme oxygenase	HMOX1	6,11
C2_61488	Heterogeneous nuclear ribonucleoprotein K	HNRNPK	6
C2_27397	Hemopexin	HPX	6
C2_4763	Heat shock cognate 71 kDa protein	HSPA8	7
C2_23533	Inhibitor of nuclear factor kappa-B kinase subunit epsilon	IKBKE	6
C2_757	Interleukin-6 receptor subunit alpha	IL6R	6
C2_5031	Ras GTPase-activating-like protein IQGAP1	IQGAP1	5,9,16,17
C2_7014	Ras GTPase-activating-like protein IQGAP2	IQGAP2	5
FP333792	Inter-alpha-trypsin inhibitor heavy chain H2	ITIH2	6
C2_12615	Inter-alpha-trypsin inhibitor heavy chain H3	ITIH3	6
C2_23019	Junction plakoglobin	JUP	9
C2_29495	Kininogen (Fragments)	KNG1	5
C2_2112	GTPase KRas	KRAS	5,6,8,9,10,11,13
C2_24867	Dual specificity mitogen-activated protein kinase kinase 6	MAP2K6	6
C2_10488	Mitogen-activated protein kinase 1	MAPK1	5,6,8,10,11,13,17
C2_15543	Microtubule-associated protein RP/EB family member 1	MAPRE1	16
C2_86	Moesin	MSN	5,14,15,17
C2_29145	Myosin-11	MYH11	5,9
C2_14197	Myosin-6	MYH6	5,9
C2_515	Myosin-9	MYH9	5,9
C2_179	Myosin light chain 1, skeletal muscle isoform	MYL1	5,9,12,14,15,17
C2_2556	Myosin light chain 3, skeletal muscle isoform	MYL3	5,9,12,14,15,17
C2_3500	Myosin light polypeptide 6	MYL6	5,9,12,14,15,17
C2_2090	Myosin regulatory light polypeptide 9	MYL9	5,9,10,12,14,15,17
C2_2234	Myosin regulatory light chain 2, smooth muscle minor isoform	MYLPF	5,12,14,15,17
C2_59686	Myosin-Ie	MYO1E	7
C2_62194	Myosin-VI	MYO6	7
C2_82048	Nuclear factor NF-kappa-B p105 subunit	NFKB1	6,17
C2_24789	Ephexin-1	NGEF	14
C2_113264	Nucleoside diphosphate kinase A1	NME1	16
C2_4961	Glucocorticoid receptor	NR3C1	6
C2_3168	Polyadenylate-binding protein 1	PABPC1	8,13
C2_3226	Serine/threonine-protein kinase PAK 2	PAK2	5,10,12,15,17
C2_2505	3-phosphoinositide-dependent protein kinase 1	PDPK1	6,8,11,13
C2_22976	Profilin-1	PFN1	5,12,14
C2_4778	1-phosphatidylinositol-3-phosphate 5-kinase	PIKFYVE	5,12,14,15,17
C2_70006	1-phosphatidylinositol-4,5-bisphosphate phosphodiesterase gamma-1	PLCG1	10
C2_4412	1-phosphatidylinositol-4,5-bisphosphate phosphodiesterase gamma-2	PLCG2	10
C2_1167	Serine/threonine-protein phosphatase PP1-beta catalytic subunit	PPP1CB	5,8,10,12,14
C2_553	Serine/threonine-protein phosphatase PP1-gamma catalytic subunit	PPP1CC	8
C2_11251	Serine/threonine-protein phosphatase 2A catalytic subunit alpha isoform	PPP2CA	11,13
C2_7368	Serine/threonine-protein phosphatase 2A catalytic subunit beta isoform	PPP2CB	11,13
C2_7698	Serine/threonine-protein phosphatase 2A 65 kDa regulatory subunit A beta isoform	PPP2R1B	11,13
C2_22338	Serine/threonine-protein phosphatase 2B catalytic subunit alpha isoform	PPP3CA	7
C2_6942	Calcineurin subunit B type 1	PPP3R1	7
C2_9126	Protein kinase C beta type	PRKCB	11
C2_3156	Ras-related protein Rab-11A	RAB11A	7
C2_452	Ras-related protein Rab-11B	RAB11B	7
C2_9276	Ras-related protein Rab-4B	RAB4B	7
C2_4291	Ras-related protein Rab-5A	RAB5A	7,16
C2_6579	Ras-related protein Rab-5B	RAB5B	7,16
C2_7828	Ras-related protein Rab-5C	RAB5C	7,16
C2_53708	Ras-related protein Rab7	RAB7A	7,16
C2_1097	Ras-related C3 botulinum toxin substrate 1	RAC1	5,7,9,10,11,12,15,17
C2_121610	Ras-related C3 botulinum toxin substrate 2	RAC2	5,10,12
C2_935	Ras-related protein Ral-B	RALB	10
C2_879	Ras-related protein Rap-1b	RAP1B	9,1
C2_16564	Radixin	RDX	5,14,15,17
C2_131	Transforming protein RhoA	RHOA	5,9,10,11,12,14,15,17
C2_30428	Rho-related GTP-binding protein RhoC	RHOC	10,11,12,15,17
C2_11478	Rho-related GTP-binding protein RhoG	RHOG	10,11,12,15,17
C2_96072	60S ribosomal protein L10	RPL10	8
C2_67	60S ribosomal protein L10a	RPL10A	8
C2_236	60S ribosomal protein L11	RPL11	8
C2_587	60S ribosomal protein L12	RPL12	8
C2_453	60S ribosomal protein L13	RPL13	8
C2_441	60S ribosomal protein L13a	RPL13A	8
C2_142	60S ribosomal protein L14	RPL14	8
C2_279	60S ribosomal protein L15	RPL15	8
C2_1244	60S ribosomal protein L17	RPL17	8
C2_24040	60S ribosomal protein L18	RPL18	8
C2_12358	60S ribosomal protein L18a	RPL18A	8
C2_700	60S ribosomal protein L19	RPL19	8
C2_94336	60S ribosomal protein L21	RPL21	8
C2_39656	60S ribosomal protein L22	RPL22	8
C2_115552	60S ribosomal protein L22-like 1	RPL22L1	8
C2_373	60S ribosomal protein L23	RPL23	8
C2_1007	60S ribosomal protein L23a	RPL23A	8
C2_119969	60S ribosomal protein L24	RPL24	8
C2_392	60S ribosomal protein L26	RPL26	8
C2_102117	60S ribosomal protein L27	RPL27	8
C2_343	60S ribosomal protein L27a	RPL27A	8
C2_1383	60S ribosomal protein L28	RPL28	8
C2_12	60S ribosomal protein L3	RPL3	8
C2_17126	60S ribosomal protein L30	RPL30	8
C2_1127	60S ribosomal protein L31	RPL31	8
C2_72598	60S ribosomal protein L34	RPL34	8
C2_89835	60S ribosomal protein L35	RPL35	8
C2_11574	60S ribosomal protein L35a	RPL35A	8
C2_2019	60S ribosomal protein L36	RPL36	8
C2_1788	60S ribosomal protein L36a	Rpl36a	8
C2_796	60S ribosomal protein L37	RPL37	8
C2_64323	60S ribosomal protein L38	RPL38	8
C2_25	60S ribosomal protein L4	RPL4	8
C2_143	60S ribosomal protein L5	RPL5	8
C2_827	60S ribosomal protein L6	RPL6	8
C2_434	60S ribosomal protein L7	RPL7	8
C2_98	60S ribosomal protein L7a	RPL7A	8
C2_174	60S ribosomal protein L8	RPL8	8
C2_156	60S ribosomal protein L9	RPL9	8
C2_47	60S acidic ribosomal protein P0	RPLP0	8
C2_46323	60S acidic ribosomal protein P1	RPLP1	8
C2_6923	60S acidic ribosomal protein P2	RPLP2	8
C2_8805	40S ribosomal protein S10	RPS10	8,11,13
C2_367	40S ribosomal protein S11	RPS11	8,11,13
C2_583	40S ribosomal protein S12	RPS12	8,11,13
C2_20708	40S ribosomal protein S14	RPS14	8,11,13
C2_10570	40S ribosomal protein S15	RPS15	8,11,13
C2_414	40S ribosomal protein S15a	RPS15A	8,11,13
C2_7337	40S ribosomal protein S16	RPS16	8,11,13
C2_971	40S ribosomal protein S17	RPS17	8,11,13
C2_17339	40S ribosomal protein S18	RPS18	8,11,13
C2_955	40S ribosomal protein S19	RPS19	8,11,13
C2_62581	40S ribosomal protein S2	RPS2	8,11,13
C2_232	40S ribosomal protein S20	RPS20	8,11,13
C2_11917	40S ribosomal protein S21	RPS21	8,11,13
C2_1243	40S ribosomal protein S23	RPS23	8,11,13
C2_1271	40S ribosomal protein S24	RPS24	8,11,13
C2_310	40S ribosomal protein S25	RPS25	8,11,13
C2_698	40S ribosomal protein S26	RPS26	8,11,13
C2_33738	Ubiquitin-40S ribosomal protein S27a	RPS27A	8,11,13
C2_50749	40S ribosomal protein S28	RPS28	8,11,13
C2_23479	40S ribosomal protein S29	RPS29	8,11,13
C2_17873	40S ribosomal protein S3	RPS3	8,11,13
C2_593	40S ribosomal protein S4	RPS4	8,11,13
C2_433	40S ribosomal protein S5	RPS5	8,11,13
C2_164	40S ribosomal protein S6	RPS6	8,11,13
C2_2840	Ribosomal protein S6 kinase 2 alpha	RPS6KA1	11
C2_38170	Ribosomal protein S6 kinase alpha-3	RPS6KA3	11
C2_133	40S ribosomal protein S7	RPS7	8,11,13
C2_13671	40S ribosomal protein S8	RPS8	8,11,13
C2_252	40S ribosomal protein S9	RPS9	8,11,13
C2_16	40S ribosomal protein SA	RPSA	8,11,13
C2_23094	Ras-related protein R-Ras	RRAS	5,6,8,9,10,11,13
C2_7026	Ras-related protein R-Ras2	RRAS2	5,6,8,9,10,11,13
C2_16505	Septin-10	SEPT10	14,17
C2_5603	Septin-2	SEPT2	14,17
C2_22270	Septin-6	SEPT6	14,17
C2_8875	Septin-7	SEPT7	14,17
C2_9888	Septin-8-A	SEPT8	14,17
C2_14294	Serpin peptidase inhibitor, clade A (alpha-1 antiproteinase, antitrypsin), member 1	SERPINA1	6,7
C2_9982	Serpin peptidase inhibitor, clade F (alpha-2 antiplasmin, pigment epithelium derived factor), member 2	SERPINF2	6
C2_9305	Endophilin-A1	SH3GL2	7
C2_4801	SHC-transforming protein 1	SHC1	5,6,8,10,13
C2_1642	Superoxide dismutase [Mn], mitochondrial	SOD2	6
C2_4186	Protein phosphatase Slingshot homolog	SSH1	5
C2_2871	Signal transducer and activator of transcription 3	STAT3	6
C2_15171	Transcription factor 7-like 2	TCF7L2	9
C2_7158	Transferrin	TF	6,7
C2_83924	Talin-1	TLN1	5,1
C2_1309	Tumor necrosis factor receptor superfamily member 1B	TNFRSF1B	6
C2_45863	Activated CDC42 kinase 1	TNK2	10
C2_2326	Tumor necrosis factor receptor type 1-associated DEATH domain protein	TRADD	6
C2_1557	Titin	TTN	5,10,14
C2_75383	Transthyretin	TTR	6
C2_38179	Tubulin alpha-1A chain	TUBA1A	9,16
C2_20113	Tubulin alpha-4A chain	TUBA4A	9,16
C2_905	Tubulin beta chain	TUBB	9,16
C2_90	Tubulin beta-1 chain	TUBB1	9,16
C2_14202	Tubulin beta-2C chain	TUBB4B	9,16
C2_6783	Ubiquitin-60S ribosomal protein L40	UBA52	8
C2_19855	Probable ubiquitin carboxyl-terminal hydrolase FAF-X	USP9X	7
C2_9594	Vinculin	VCL	5,9,10,16
C2_3381	Vimentin	VIM	17

*Canonical pathways are indicated by number: (5) Actin cytoskeleton signaling; (6) Acute phase response signaling; (7) Clathrin-mediated endocytosis signaling; (8) EIF2 signaling; (9) Epithelial adherens junction signaling; (10) Integrin signaling; (11) mTOR signaling; (12) Regulation of actin based motility by Rho; (13) Regulation of eIF4 and p70S6K signaling; (14) RhoA signaling; (15) RhoGDI signaling; (16) Remodeling of epithelial adherens junctions; (17) Signaling by Rho family GTPase*.

### Stress-regulated proteins

**Figure 2:**
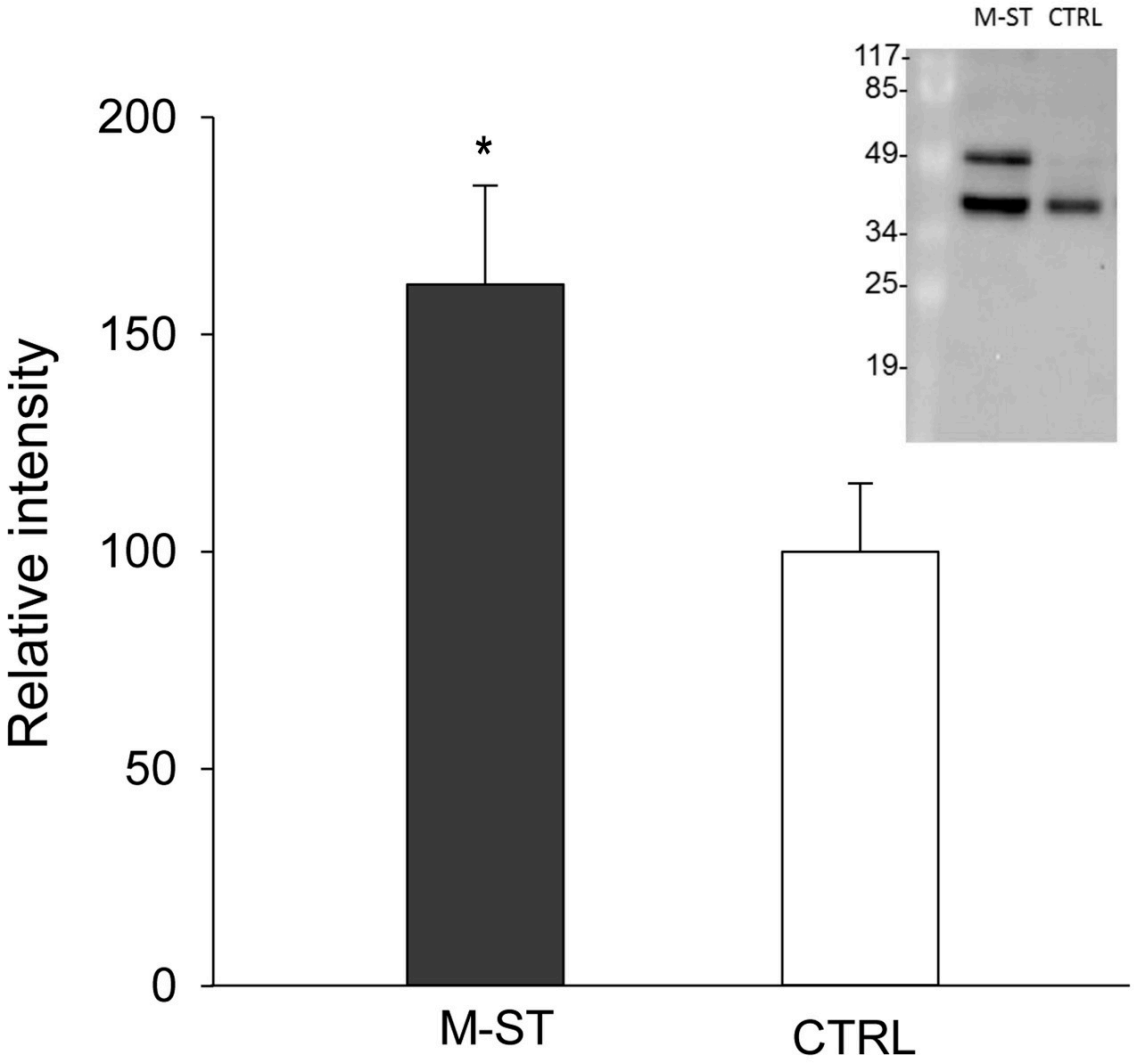
**Relative keratin type II cytoskeletal 8 protein levels in skin mucus of multiple sensorial stressed fish (M-ST) and control unstressed fish (CTRL)**. Values of expression relative to control are the mean ± SEM of eight individuals. Asterisk indicated significant differences (*p* < 0.05, Student's *t*-test) between groups. Insert shows a representative western blot using the rabbit anti-human cytokeratin 8 antibody.

Principal components analysis from image processing of 2-DE of the mucus proteins from control CTRL vs. M-ST did not clearly separate individuals from both groups (Figure S1). Thus, only six spots were found to show a different significant (*p*-value < 0.03) abundance in stressed fish, with three upregulated (fold-change 1.6–2.7) and three down-regulated (0.6–0.7) proteins. The six protein spots were unequivocally identified by comparing the LC-MS/MS data with the gilthead sea bream transcriptome database, with a 100% of identity for all peptide sequences with the corresponding accession (Table 4). Down-regulated spots were elongation factor 2 (spot 743; GenBank accession KY388506) and cytoplasmic actin (spots 1,549 and 1,816; GenBank accession KY388507). Spot 2,181 (fold-change 1.64) was identified as the mitochondrial protein cytochrome c1 heme (GenBank accession KC217621), whereas the two most upregulated spots (spots 815, 1,321) were both recognized as keratin type II cytoskeletal 8 (GenBank accession KY388508). The higher abundance of immunoreactive cytokeratin 8 proteins in the mucus of M-ST fish was confirmed by Western blot (Figure 2), where the most abundant band was that of lower molecular weight (38–40 kDa). Cytokeratin 8 is a highly modified protein, but our working hypothesis is that this band was a proteolytically cleaved form. Protein spots, representing type I or type II keratin fragments, have also been reported at different stages of development in amphibians (Domanski and Helbing, 2007) and Atlantic cod larvae (Sveinsdóttir et al., 2008). Likewise, different fragments of cytokeratin 8 were detected by immunoblotting in colorectal biopsies of human cancer patients (Khan et al., 2011). Of note, gilthead sea bream cytokeratine 8 has a high identity (61%) and homology (69%) with the same protein of human origin, but the identified peptide sequences matched exactly with the gilthead sea bream protein sequence and not with that of human, so the risk of potential handling contamination was discarded.

**Table 4:** **Protein spots identified as differentially expressed in gilthead sea bream skin mucus after multiple sensorial stress**.

**Spot number**	**Accession number**	**Protein name**	***p*-value**	**Average ratio (M-ST/CTRL)**	**Identified peptide sequences**
743	C2_534	Elongation factor 2	0.026	0.71	APLMVYISK/CDLLYEGPPDDEAAMGIK/EGVLCEENMR/FSVSPVVR/GGG QIIPTAR/GGGQIIPTARR/NCDSKAPLMVYISK/RVLYACELTAEPR/SDPVVS YR/TILMMGR/VAVEAKNPADLPK/VFSGSVSTGLK/VFSGSVSTGLKVR/VLYACEL TAEPR/VMKFSVSPVVR
815	C2_1442	Keratin type II cytoskeletal 8	0.014	2.71	ANLEAQIAEAEER/AQYEDIANR/FASFIDKVR/IRDLEDALQR/NLDMDSIVAEVK
1,321	C2_1442	Keratin type II cytoskeletal 8	0.024	1.78	DTSVIVEMDNSR/FASFIDKVR/FLEQQNK/IRDLEDALQR/LALDIEIATYRK/NM QGLVEDFK/YEDEINK/YEDEINKR
1,549	C2_2	Actin, cytoplasmic 1	0.019	0.71	AGFAGDDAPR/AVFPSIVGRPR/DLTDYLMK/IIAPPERK/LAPSTMKIK/SYELP DGQVITIGNER
1,816	C2_2	Actin, cytoplasmic 1	0.026	0.63	DLYANTVLSGGTTMYPGIADR/GYSFTTTAER/SYELPDGQVITIGNER/VAPEE HPVLLTEAPLNPK/VAPEEHPVLLTEAPLNPKANR
2,181	C2_785	Cytochrome c1, heme protein mitochondrial	0.018	1.64	LSDYFPKPYPNPESAR/NLVGVSHTEAEVK

Clear evidence for the prominent mechanical function of keratins comes from multiple human diseases and murine knockouts. However, distinct keratins emerge as highly dynamic scaffolds contributing to cell size determination, translation control, proliferation, malignant transformation and various stress responses (Magin et al., 2007; Loschke et al., 2015). Importantly, this also applies to fish and different reports show that keratins from skin mucus possess anti-bacterial activity owing to their pore-forming properties (Molle et al., 2008; Rajan et al., 2011). Relatively little is known about the precise mechanisms responsible for assembly and pathology, although it has been suggested that keratins can act as a “phosphate sponge” absorbing the stress-activated phosphate kinases, thereby, reducing their adverse effect and protecting cells from injury (Ku and Omary, 2006). Indeed, differential regulation of keratin phosphorylation is related to intricate functional properties of specific epithelial cell types (Tao et al., 2006; Busch et al., 2012; Majumdar et al., 2012). In our case, changes in the abundance of cytokeratin 8 in the skin mucus of gilthead sea bream would support some type of epithelia damage in fish diagnosed as chronically stressed, showing reduced growth and feed conversion efficiency, strong-down regulation of markers of mitochondrial activity and biogenesis in combination with a high variable and non-significant increase of plasma cortisol levels (Bermejo-Nogales et al., 2014). Since aerobic metabolism is the most important source of reactive oxygen species (ROS), this mitochondrial metabolic feature was considered as part of the adaptive stress response that reduced ROS production when fish face an increased risk of oxidative stress in our stress model that mimicked daily farming activities. The magnitude of the changes observed in the skin mucus proteome was, however, lower than expected. It can be argued that this fact might reflect the high allostatic capacity of our fish strain to cope with chronic stress. Indeed, in other less intrusive models of chronic stress, fish were intensively chased for 5 min 2 times per day after lowering water level and data on growth parameters evidenced a real stress adaptation with a switch from aerobic to more anaerobic metabolism without changes in plasma cortisol levels (Bermejo-Nogales et al., 2014). Additionally, other factors including season, age and nutritional background should be considered in an holistic manner to ultimately understand the extent to which the skin mucus proteome of gilthead sea bream is regulated by environmental and nutritional stressors, helping to understand how stress condition can be fine evaluated at the farm scale level without evoking further stress.

## Conclusions

A high resolution mass spectrometry-based proteomic approach was able to identify 2,062 proteins in the skin mucus of gilthead bream after matching in a homologous protein database. Three major clusters with more than 350 proteins were retained after filtering by canonical pathway overlapping. Among them, proteins of oxidative phosphorylation, mitochondrial dysfunction, protein ubiquitination, immune response, epithelial remodeling, and cellular assembly were highly represented. This was reinforced by the observation that major changes related to the abundance of cytokeratin 8 in the skin mucus of stressed fish under our experimental model of chronic stress were found by means of 2-DE methodology and confirmed by immunoblotting. All this information will be useful in developing more targeted approaches that address specific changes in the skin mucus proteome of farmed fish, with special emphasis on markers of skin epithelial cell turnover.

## Author contributions

JP, RO, and AS conceived and designed the study. OF supervised animal handling and sampling. JP, GT, PS, SR, and JC performed protein identification and functional characterization of mucus proteome and stress-regulated proteins. JP and PS conducted Western blot analysis. JP, GT, AS, and JC wrote the manuscript. All authors read and approved the final manuscript.

## Funding

This study was funded by the European Union (AQUAEXCEL, FP7/2007/2013; grant agreement No. 262336, Aquaculture infrastructures for excellence in European fish research) project. The views expressed in this work are the sole responsibility of the authors and do not necessarily reflect the views of the European Commission. Additional funding was obtained from the Spanish Ministerio de Economía y Competitividad (MI2-Fish, AGL2013-48560) and from Generalitat Valenciana (PROMETEO FASE II-2014/085). Proteomics study was done at Proteomics laboratory of University of Valencia, Spain (SCSIE). This laboratory is a member of Proteored, PRB2-ISCIII and is supported by grant PT13/0001, of the PE I+D+i 2013–2016, funded by Instituto de Salud Carlos III and Fondo Europeo de Desarrollo Regional (FEDER).

### Conflict of interest statement

The authors declare that the research was conducted in the absence of any commercial or financial relationships that could be construed as a potential conflict of interest.
