# Supplementary material for: Skin Mucus of Gilthead Sea Bream (Sparus aurata L.). Protein Mapping and Regulation in Chronically Stressed Fish
Source: Front Physiol. 2017 Feb 1;8:34. doi: 10.3389/fphys.2017.00034 (PMC5288811; doi:10.3389/fphys.2017.00034)
Supplement: Supplementary file 1 [file Table1.PDF]

Table S1. Identified proteins from gilthead sea bream skin mucus. S/C indicates the total protein score (S) and percentage of coverage (C) for each identified protein. U/T indicates the number of total unique peptides (U) against total matched peptides (T)

Accession number	Protein annotation	S/C	U/T
C2_6951	[Pyruvate dehydrogenase [lipoamide]] kinase isozyme 2, mitochondrial	30/7.8	9/10
C2_1480	1,2-dihydroxy-3-keto-5-methylthiopentene dioxygenase	40/3.7	4/6
C2_4023	10 kDa heat shock protein, mitochondrial	169/40.7	9/12
C2_4764	116 kDa U5 small nuclear ribonucleoprotein component	101/12.5	4/4
C2_99849	14-3-3 protein beta/alpha	113/28.2	5/7
C2_2298	14-3-3 protein beta/alpha-1	583/17.4	13/43
C2_268	14-3-3 protein epsilon	1046/19.8	19/63
C2_2474	14-3-3 protein gamma-1	344/13.9	14/48
C2_1017	14-3-3 protein zeta	1408/26	22/99
C2_1229	14-3-3-like protein 1	2273/29.2	26/112
C2_34474	14-3-3-like protein 2	772/29	10/43
C2_2006	15 kDa selenoprotein	65/5.2	3/4
C2_4902	17-beta-hydroxysteroid dehydrogenase 14	112/7.3	8/10
C2_43940	1-acyl-sn-glycerol-3-phosphate acyltransferase epsilon	33/7	2/2
C2_4093	1-acyl-sn-glycerol-3-phosphate acyltransferase gamma	145/4.4	7/9
C2_4778	1-phosphatidylinositol-3-phosphate 5-kinase	51/4.7	3/6
C2_12986	1-phosphatidylinositol-4,5-bisphosphate phosphodiesterase delta-1	268/17.1	16/18
C2_70006	1-phosphatidylinositol-4,5-bisphosphate phosphodiesterase gamma-1	32/4.8	2/7
C2_4412	1-phosphatidylinositol-4,5-bisphosphate phosphodiesterase gamma-2	39/3.5	6/15
C2_3170	2,4-dienoyl-CoA reductase, mitochondrial	45/3	3/5
C2_1520	26S protease regulatory subunit 10B	111/12.8	8/9
C2_4264	26S protease regulatory subunit 4	288/25	13/18
C2_1666	26S protease regulatory subunit 6A	90/12.3	5/5
C2_482	26S protease regulatory subunit 6B	60/8.1	4/9
C2_3002	26S protease regulatory subunit 7	103/13.3	6/11
C2_514	26S protease regulatory subunit 8	233/23.2	9/13
C2_2728	26S proteasome non-ATPase regulatory subunit 1	928/21.9	20/55
C2_3102	26S proteasome non-ATPase regulatory subunit 11	113/12.7	11/20
C2_1392	26S proteasome non-ATPase regulatory subunit 12	463/21.1	16/26
C2_790	26S proteasome non-ATPase regulatory subunit 13	31/4.2	2/2
C2_807	26S proteasome non-ATPase regulatory subunit 14	105/21.1	6/9
C2_4556	26S proteasome non-ATPase regulatory subunit 2	1091/20.9	22/57
C2_1006	26S proteasome non-ATPase regulatory subunit 3	424/34.5	20/31
C2_8032	26S proteasome non-ATPase regulatory subunit 6	362/27.5	12/19
C2_364	26S proteasome non-ATPase regulatory subunit 7	71/7	4/4
C2_1843	26S proteasome non-ATPase regulatory subunit 8	324/16.5	11/12
C2_7280	28S ribosomal protein S27, mitochondrial	39/4.8	3/3
C2_10322	2-aminoethanethiol dioxygenase	40/9.2	6/10
C2_19129	2-oxoglutarate dehydrogenase, mitochondrial	186/19.6	7/11
C2_44272	2-oxoglutarate dehydrogenase-like, mitochondrial	135/9.4	7/16
C2_2479	2-oxoisovalerate dehydrogenase subunit alpha, mitochondrial	133/12.9	7/10
C2_2676	2-oxoisovalerate dehydrogenase subunit beta, mitochondrial	44/3.8	1/2
C2_704	39S ribosomal protein L18, mitochondrial	35/7.9	3/4
C2_1416	3-hydroxyacyl-CoA dehydratase 2	32/11.9	6/8
C2_7568	3-hydroxyacyl-CoA dehydrogenase type-2	147/30.7	6/6
C2_850	3-hydroxybutyrate dehydrogenase type 2	182/21	7/8
C2_1580	3-hydroxyisobutyrate dehydrogenase, mitochondrial	97/8.6	5/8
C2_742	3-oxo-5-beta-steroid 4-dehydrogenase	38/9.2	5/6

C2_8112	3-oxoacyl-[acyl-carrier-protein] reductase FabG	139/29.4	7/8
C2_2505	3-phosphoinositide-dependent protein kinase 1	43/5.6	7/8
C2_8805	40S ribosomal protein S10	418/45.1	11/28
C2_367	40S ribosomal protein S11	634/34.7	15/41
C2_583	40S ribosomal protein S12	318/43.1	9/18
C2_14171	40S ribosomal protein S13	1474/35.3	18/54
C2_20708	40S ribosomal protein S14	522/36.8	10/23
C2_10570	40S ribosomal protein S15	190/18.8	4/12
C2_414	40S ribosomal protein S15a	713/28.9	13/42
C2_7337	40S ribosomal protein S16	1479/60.4	22/85
C2_971	40S ribosomal protein S17	326/31.1	6/15
C2_17339	40S ribosomal protein S18	656/34.7	16/45
C2_955	40S ribosomal protein S19	884/53.2	14/44
C2_684	40S ribosomal protein S2	1599/50.2	15/81
C2_62581	40S ribosomal protein S2	265/55.4	3/7
C2_232	40S ribosomal protein S20	422/6.2	7/14
C2_11917	40S ribosomal protein S21	206/11.8	2/4
C2_1243	40S ribosomal protein S23	347/24.9	6/24
C2_1271	40S ribosomal protein S24	298/19.8	3/8
C2_310	40S ribosomal protein S25	358/11.6	9/18
C2_698	40S ribosomal protein S26	571/23.9	7/33
C2_175	40S ribosomal protein S27	254/20.8	5/18
C2_50749	40S ribosomal protein S28	281/14.8	7/11
C2_23479	40S ribosomal protein S29	54/15.6	4/4
C2_17873	40S ribosomal protein S3	2016/74	25/113
C2_2139	40S ribosomal protein S3a	2126/58.5	22/93
C2_593	40S ribosomal protein S4	1725/38.3	24/96
C2_23275	40S ribosomal protein S4, X isoform	1132/62.8	14/63
C2_433	40S ribosomal protein S5	795/33.7	12/38
C2_164	40S ribosomal protein S6	416/30.3	10/34
C2_133	40S ribosomal protein S7	917/34.5	18/54
C2_13671	40S ribosomal protein S8	1201/47.9	13/58
C2_252	40S ribosomal protein S9	609/35.8	19/64
C2_16	40S ribosomal protein SA	1226/25.9	11/39
C2_4721	4-aminobutyrate aminotransferase, mitochondrial	35/3.1	6/7
C2_10695	52 kDa repressor of the inhibitor of the protein kinase	47/5.4	4/6
C2_4852	6.8 kDa mitochondrial proteolipid	43/5.1	1/1
C2_5222	60 kDa heat shock protein, mitochondrial	311/15.4	14/16
C2_47	60S acidic ribosomal protein P0	1239/48.1	15/47
C2_46323	60S acidic ribosomal protein P1	78/13.6	2/4
C2_6923	60S acidic ribosomal protein P2	45/14	2/2
C2_167	60S ribosomal protein L10	4128/35.6	22/169
C2_96072	60S ribosomal protein L10	167/31.3	8/15
C2_67	60S ribosomal protein L10a	1008/46.7	14/42
C2_236	60S ribosomal protein L11	490/26	7/24
C2_587	60S ribosomal protein L12	1083/36	10/38
C2_453	60S ribosomal protein L13	329/18.7	11/25
C2_441	60S ribosomal protein L13a	135/19.8	15/34
C2_142	60S ribosomal protein L14	575/23	8/19
C2_279	60S ribosomal protein L15	572/24.6	12/48
C2_1244	60S ribosomal protein L17	549/32.4	15/36
C2_24040	60S ribosomal protein L18	655/49.3	12/37
C2_12358	60S ribosomal protein L18a	669/47.8	14/42
C2_700	60S ribosomal protein L19	139/24	7/13
C2_94336	60S ribosomal protein L21	369/37.6	7/15

C2_39656	60S ribosomal protein L22	308/15.8	5/10
C2_115552	60S ribosomal protein L22-like 1	34/12.7	2/3
C2_373	60S ribosomal protein L23	469/44.7	9/24
C2_1007	60S ribosomal protein L23a	370/32.2	9/23
C2_119969	60S ribosomal protein L24	162/35.4	7/22
C2_392	60S ribosomal protein L26	456/30.5	14/30
C2_102117	60S ribosomal protein L27	253/43.1	7/15
C2_343	60S ribosomal protein L27a	492/32.2	6/15
C2_1383	60S ribosomal protein L28	487/44.9	14/29
C2_7015	60S ribosomal protein L29	56/4.2	2/11
C2_12	60S ribosomal protein L3	1261/33.9	24/91
C2_17126	60S ribosomal protein L30	775/40.2	7/28
C2_1127	60S ribosomal protein L31	163/27	9/17
C2_19060	60S ribosomal protein L32	192/20.1	4/7
C2_72598	60S ribosomal protein L34	188/19.2	9/25
C2_89835	60S ribosomal protein L35	149/33.1	5/12
C2_11574	60S ribosomal protein L35a	192/35	9/18
C2_2019	60S ribosomal protein L36	34/11.1	2/3
C2_1788	60S ribosomal protein L36a	66/23.5	6/10
C2_796	60S ribosomal protein L37	41/7.2	2/3
C2_64323	60S ribosomal protein L38	213/32.6	4/12
C2_25	60S ribosomal protein L4	1088/28.7	17/56
C2_143	60S ribosomal protein L5	430/37.4	18/45
C2_827	60S ribosomal protein L6	646/35.4	12/39
C2_434	60S ribosomal protein L7	432/36.2	11/35
C2_98	60S ribosomal protein L7a	1207/30.9	12/44
C2_174	60S ribosomal protein L8	927/46	16/46
C2_156	60S ribosomal protein L9	543/17.9	12/32
C2_3317	6-phosphofructokinase type C	45/4.8	3/5
C2_4969	6-phosphofructokinase, liver type	73/7	8/11
C2_3427	6-phosphofructokinase, muscle type	45/3.8	4/8
C2_531	6-phosphogluconate dehydrogenase, decarboxylating	568/18.3	10/20
C2_6550	6-phosphogluconolactonase	67/5.7	2/2
C2_14692	6-pyruvoyl tetrahydrobiopterin synthase	49/4.7	2/2
C2_25027	78 kDa glucose-regulated protein	1080/29.1	8/32
C2_1823	Abhydrolase domain-containing protein 10, mitochondrial	137/12.3	5/7
C2_40874	Abhydrolase domain-containing protein 14B	181/28	4/6
C2_4551	Abhydrolase domain-containing protein 16A	45/3.7	4/6
C2_7395	Abhydrolase domain-containing protein 2-B	36/5.6	8/16
C2_85224	Abnormal spindle-like microcephaly-associated protein homolog	31/6.9	1/1
C2_18371	Abnormal spindle-like microcephaly-associated protein homolog	38/8.9	5/7
C2_37579	Absent in melanoma 1 protein	43/10.4	3/8
C2_5203	Acetylcholine receptor subunit alpha	1761/14.3	16/91
C2_18254	Acetylcholine receptor subunit gamma	31/8.1	9/17
C2_53898	Acetyl-CoA acetyltransferase, cytosolic	47/7.5	4/5
C2_181	Acetyl-CoA acetyltransferase, mitochondrial	54/3.8	3/3
C2_44535	Acetyl-CoA carboxylase 1	67/15.1	3/4
C2_78963	Acetyl-CoA carboxylase 2	50/11.1	2/4
C2_14060	Acetyl-coenzyme A synthetase, cytoplasmic	78/8.1	5/7
C2_51380	Achaete-scute homolog 5	40/6.7	1/3
C2_2879	Acid ceramidase	76/7.8	7/12
C2_405	Acidic leucine-rich nuclear phosphoprotein 32 family member A	50/5.1	3/3
C2_18809	Aconitase 2, mitochondrial	78/16	6/8
C2_119067	Actin	4639/28.6	19/256
C2_1387	Actin, alpha cardiac	4714/37	19/267

C2_2	Actin, cytoplasmic 1	9076/40.5	26/383
C2_102126	Actin, cytoplasmic 2	6696/39.9	21/286
C2_29626	Actin, non-muscle 6.2	6218/54.1	19/253
C2_89711	Actin-binding LIM protein 2	34/3.1	1/1
C2_21312	Actin-binding protein IPP	53/5.1	4/6
C2_1852	Actin-related protein 2	126/8.5	5/11
C2_18222	Actin-related protein 2/3 complex subunit 1A	742/37.6	15/47
C2_4212	Actin-related protein 2/3 complex subunit 1B	82/5.6	4/7
C2_22529	Actin-related protein 2/3 complex subunit 2	276/21	10/19
C2_4166	Actin-related protein 2/3 complex subunit 3	314/24.1	7/12
C2_427	Actin-related protein 2/3 complex subunit 4	402/19.2	7/31
C2_503	Actin-related protein 2/3 complex subunit 5	175/13.6	11/14
C2_3453	Actin-related protein 2-A	330/25.3	12/27
C2_1771	Actin-related protein 3	426/20.7	11/19
C2_45863	Activated CDC42 kinase 1	34/6.6	3/5
C2_11239	Acyl carrier protein, mitochondrial	42/3.4	1/1
C2_2532	Acylamino-acid-releasing enzyme	250/19.6	16/25
C2_3692	Acyl-CoA dehydrogenase family member 9, mitochondrial	57/5.7	5/10
C2_8427	Acyl-CoA synthetase family member 2, mitochondrial	261/12.4	10/15
C2_649	Acyl-CoA-binding protein	36/5.7	3/13
C2_38044	Acyl-coenzyme A thioesterase 10, mitochondrial	30/18.7	4/5
C2_1648	Acyl-protein thioesterase 1	114/7.7	7/12
C2_584	Adaptin ear-binding coat-associated protein 2	75/10.5	7/8
C2_2187	Adenosylhomocysteinase A	1709/24.5	19/89
C2_121631	Adenosylhomocysteinase B	549/24.4	5/23
C2_6468	Adenylate kinase 2, mitochondrial	219/27.1	13/24
C2_83	Adenylate kinase isoenzyme 1	149/15.6	9/13
C2_45578	Adenylate kinase isoenzyme 4, mitochondrial	100/24.5	7/8
C2_6546	Adenylosuccinate lyase	53/5.6	4/4
C2_59354	Adenylosuccinate synthetase	180/25.5	4/6
C2_41283	Adenylyl cyclase-associated protein 1	1344/53.9	10/42
C2_5806	Adenylyl cyclase-associated protein 2	48/4.5	5/9
C2_93073	ADP,ATP carrier protein	1277/40.3	11/69
C2_89214	ADP,ATP carrier protein 1	460/15.9	5/26
C2_927	ADP/ATP translocase 2	1264/28.6	12/60
C2_19743	ADP/ATP translocase 3	486/24.9	9/48
C2_1001	ADP-ribosylation factor 1	619/8.7	7/29
C2_487	ADP-ribosylation factor 1-like 2	609/16.9	10/32
C2_2038	ADP-ribosylation factor 4	782/22.7	10/35
C2_3418	ADP-ribosylation factor 6	94/11.5	7/9
C2_12596	ADP-ribosylation factor GTPase-activating protein 2	76/8.1	10/24
C2_1966	ADP-ribosylation factor-like protein 1	118/8.6	7/12
C2_58720	ADP-ribosylation factor-like protein 11	51/19.3	4/12
C2_59465	ADP-ribosylation factor-like protein 6-interacting protein 1	88/7.4	4/11
C2_20513	ADP-ribosylation factor-like protein 8A	115/12.1	3/4
C2_2357	ADP-ribosylation factor-like protein 8B	77/7.2	5/12
C2_1343	ADP-ribosylation factor-like protein 8B-A	79/9.1	4/5
C2_5000	ADP-ribosylation factor-like protein 9	54/6.2	6/7
C2_3920	ADP-sugar pyrophosphatase	190/9.8	6/8
C2_21659	AFG3-like protein 2	38/8.6	4/8
C2_970	Aflatoxin B1 aldehyde reductase member 2	332/28.2	12/14
C2_1483	AH receptor-interacting protein	40/2.9	4/5
C2_26362	A-kinase anchor protein 1, mitochondrial	75/10.8	4/22
C2_83267	A-kinase anchor protein 9	52/12.4	4/11
C2_4628	Alanyl-tRNA synthetase, cytoplasmic	209/16	12/15

C2_288	Alcohol dehydrogenase class-3	238/22.6	8/19
C2_399	Aldehyde dehydrogenase, mitochondrial	83/7.6	10/12
C2_99868	Aldo-keto reductase family 1 member C23-like protein	37/7.5	2/10
C2_1189	Aldose reductase	679/28	16/31
C2_2742	Alpha-1,3-mannosyl-glycoprotein 2-beta-N-acetylglucosaminyltransferase	100/8.2	6/6
C2_6331	Alpha-1-macroglobulin	735/22	16/41
C2_22548	Alpha-2-HS-glycoprotein	166/9.6	5/6
s_flp0005a11_f_1	Alpha-2-macroglobulin	46/7.6	1/2
C2_23524	Alpha-2-macroglobulin-like protein 1	250/5.9	3/7
C2_101470	Alpha-actinin-1	470/16.8	3/14
C2_1801	Alpha-actinin-3	1145/10.8	16/58
C2_26557	Alpha-actinin-4	2873/66.5	38/124
C2_1946	Alpha-aminoadipic semialdehyde dehydrogenase	374/18.1	11/21
C2_7345	Alpha-aminoadipic semialdehyde synthase, mitochondrial	68/4.3	4/4
C2_440	Alpha-enolase	635/28.1	14/40
C2_23595	Alpha-glucosidase 2	67/3.9	3/4
C2_7302	Alpha-N-acetylglucosaminidase	58/2.7	3/4
C2_1920	Alpha-soluble NSF attachment protein	290/12.9	8/9
C2_616	Aminoacyl tRNA synthase complex-interacting multifunctional protein 1	64/10.9	3/3
C2_12687	Aminoacyl tRNA synthase complex-interacting multifunctional protein 2	196/25.1	12/15
C2_17008	Anaphase-promoting complex subunit 11	43/9.1	2/10
C2_9282	Anaphase-promoting complex subunit 4	44/5.8	6/9
C2_13475	Androgen receptor	52/5	9/15
C2_2687	Angiotensin-converting enzyme	45/3.6	3/7
C2_17965	Angiotensinogen	48/8.2	2/3
C2_80860	Anion exchange protein 2	40/23.7	3/10
C2_98249	Ankyrin repeat and FYVE domain-containing protein 1	52/8	1/1
C2_16025	Ankyrin repeat and LEM domain-containing protein 2	31/3.3	3/4
C2_13947	Ankyrin repeat and MYND domain-containing protein 2	36/3.2	3/5
C2_16799	Ankyrin repeat and SOCS box protein 10	30/1.5	1/2
C2_3010	Ankyrin repeat domain-containing protein 11	38/4.1	7/12
C2_14715	Ankyrin repeat domain-containing protein 12	38/5.9	7/8
C2_39108	Ankyrin repeat domain-containing protein 50	43/5	3/13
C2_1550	Annexin A1	1078/41.7	23/56
C2_2096	Annexin A11	375/17.5	14/23
C2_3842	Annexin A13	111/3.1	2/5
C2_2251	Annexin A2	234/24.4	11/11
C2_930	Annexin A3	120/16.4	10/13
C2_88	Annexin A4	1605/47.8	23/70
C2_772	Annexin A5	548/28.7	14/19
C2_4250	Annexin A6	38/6.6	6/12
C2_98385	Anoctamin-10	32/10.2	4/4
C2_4218	Anterior gradient protein 2 homolog	889/22.9	13/74
AM955988	Anterior gradient protein 3 homolog	102/24.5	5/10
C2_6616	Antigen peptide transporter 1	66/9.2	9/9
C2_8891	Antigen peptide transporter 2	118/12	10/16
C2_17955	Antimicrobial peptide NK-lysin	44/14.9	3/5
C2_11830	AP-1 complex subunit beta-1	69/19.9	7/8
C2_8787	AP-1 complex subunit gamma-1	177/8.7	12/14
C2_2248	AP-1 complex subunit mu-2	38/8.5	6/6
C2_9477	AP-1 complex subunit sigma-1A	105/8.1	12/13
C2_5784	AP-2 complex subunit beta	42/2.2	2/5

C2_1474	AP-2 complex subunit mu-1-A	33/6.6	7/8
C2_21922	AP-4 complex subunit epsilon-1	39/4.4	3/7
C2_44642	Apelin receptor A	35/3.6	2/3
C2_6208	Apical endosomal glycoprotein	40/4	5/17
C2_1042	Apolipoprotein A-I	2471/48.8	29/123
C2_4177	Apolipoprotein A-I-binding protein	91/10	7/8
s_rl0001e11_f_1	Apolipoprotein B-100	34/3.9	1/1
C2_5591	Apolipoprotein Eb	295/25.6	11/20
C2_9385	Apoptosis inhibitor 5	83/6.1	6/7
C2_2702	Apoptosis regulator BAX	31/3.5	3/7
C2_322	Apoptosis-associated speck-like protein containing a CARD	129/20.6	6/8
C2_6260	Apoptosis-inducing factor 1, mitochondrial	52/6.1	4/6
C2_6887	Apoptosis-inducing factor 3	30/3.6	3/3
C2_489	Arachidonate 12-lipoxygenase, 12R-type	727/24	21/39
C2_4550	Arf-GAP with dual PH domain-containing protein 1	39/1.2	2/5
C2_1567	Arf-GAP with Rho-GAP domain, ANK repeat and PH domain-containing protein 1	62/2.4	3/4
FM156976	Arf-GAP with SH3 domain, ANK repeat and PH domain-containing protein 1	43/4.2	1/3
C2_2079	Argininosuccinate synthase	38/7.3	5/5
C2_19914	Arginyl-tRNA synthetase, cytoplasmic	84/13.2	5/6
C2_20526	Armadillo repeat-containing protein 5	46/3.4	2/14
C2_43004	Arylsulfatase B	37/6.2	1/1
C2_3390	Asparaginyl-tRNA synthetase, cytoplasmic	197/14.2	16/23
C2_645	Aspartate aminotransferase, mitochondrial	233/15.5	13/26
C2_23374	Aspartate beta-hydroxylase domain-containing protein 2	32/6.9	7/12
C2_15853	Aspartyl aminopeptidase	104/21.1	11/12
C2_2153	Aspartyl-tRNA synthetase, cytoplasmic	1337/38.3	25/70
C2_17558	Atlastin-2	40/7.5	2/3
C2_23566	Atlastin-3	1223/44.9	27/59
C2_958	ATP synthase lipid-binding protein, mitochondrial	45/11.2	4/5
C2_106937	ATP synthase subunit a	54/12.6	2/2
C2_1751	ATP synthase subunit alpha, mitochondrial	3966/55.1	37/158
C2_176	ATP synthase subunit b, mitochondrial	234/25.3	11/13
C2_1973	ATP synthase subunit beta, mitochondrial	2538/45.8	20/84
C2_6236	ATP synthase subunit d, mitochondrial	133/36.9	6/6
C2_6419	ATP synthase subunit delta, mitochondrial	62/10.2	2/2
C2_7051	ATP synthase subunit e, mitochondrial	34/11	4/4
C2_24277	ATP synthase subunit epsilon, mitochondrial	97/29.4	4/5
C2_1188	ATP synthase subunit f, mitochondrial	240/12.7	2/6
C2_1627	ATP synthase subunit g, mitochondrial	200/36.3	6/11
C2_18579	ATP synthase subunit gamma, mitochondrial	376/25.1	9/19
C2_123	ATP synthase subunit O, mitochondrial	791/44.6	13/32
C2_7409	ATP-binding cassette sub-family A member 2	80/7.7	8/11
C2_7005	ATP-binding cassette sub-family D member 3	47/3.5	7/12
C2_2323	ATP-binding cassette sub-family E member 1	140/5.6	5/6
C2_8958	ATP-citrate synthase	138/6.5	7/15
C2_1283	ATP-dependent RNA helicase DDX39A	480/29.9	13/37
C2_20811	ATP-dependent RNA helicase DDX3Y	139/11.7	4/5
C2_6410	ATP-dependent RNA helicase ded1	31/2.1	1/1
C2_5803	Autophagy-related protein 16	40/2.8	2/5
C2_1579	Band 3 anion exchange protein	69/2.8	3/3
C2_6477	Band 4.1-like protein 3	52/4.7	6/8
C2_10232	Barrier-to-autointegration factor-like protein	87/4.1	3/4
C2_871	Basic leucine zipper and W2 domain-containing protein 1-A	114/11.4	7/13

C2_5012	Basic leucine zipper and W2 domain-containing protein 2	42/6	3/6
C2_751	Basigin	173/6.5	7/13
C2_4217	B-cell receptor-associated protein 29	117/6.3	5/7
C2_4752	B-cell receptor-associated protein 31	370/14.3	20/35
C2_71540	Bcl-2-like protein 13	38/13.1	2/2
C2_59771	BCL-6 corepressor	31/4.7	2/4
C2_55399	Beta-1,3-galactosyltransferase 6	169/9.2	2/11
C2_28	Beta-2-microglobulin	102/8.1	7/9
C2_19834	Beta-actin-like protein 2	4717/42.4	21/270
C2_370	Beta-enolase	838/46.1	20/50
C2_111	Beta-galactoside-binding lectin	48/2.5	1/1
C2_3871	Beta-hexosaminidase subunit beta	68/10.5	8/8
C2_43304	Betaine--homocysteine S-methyltransferase 1	755/45.3	15/50
C2_3176	Bifunctional 3'-phosphoadenosine 5'-phosphosulfate synthase 2	172/4.2	5/31
C2_4884	Bifunctional aminoacyl-tRNA synthetase	599/27.5	20/37
C2_3912	Bifunctional apoptosis regulator	37/4.4	4/4
C2_4731	Bifunctional protein NCOAT	91/7.5	6/7
C2_4254	Bifunctional purine biosynthesis protein PURH	191/21.2	12/14
C2_73027	Bile salt-activated lipase	37/4.3	3/4
C2_1589	Biliverdin reductase A	39/8.2	3/4
C2_18559	Bone morphogenetic protein 1	67/8.4	5/11
C2_544	Brain protein 44	51/6.9	6/6
C2_2554	Brain protein 44-like protein	44/9.7	6/6
C2_13150	Branched-chain-amino-acid aminotransferase, cytosolic	973/14.6	14/54
C2_692	BRCA2 and CDKN1A-interacting protein	30/6.8	3/3
C2_8990	BRO1 domain-containing protein BROX	39/2.9	3/3
C2_21849	Bromodomain and PHD finger-containing protein 3	46/4.4	2/21
C2_1534	BTB/POZ domain-containing adapter for CUL3-mediated RhoA degradation protein 3	34/5.4	4/4
C2_6074	BTB/POZ domain-containing protein 2	37/6.6	3/23
C2_9940	Butyrophilin subfamily 1 member A1	41/9.6	5/7
C2_65091	Butyrophilin subfamily 2 member A3	41/8.2	2/2
C2_10965	Butyrophilin subfamily 3 member A3	41/5.5	3/3
C2_22710	CAAX prenyl protease 1 homolog	47/15.5	8/14
C2_2045	Cadherin-1	454/16.6	17/21
C2_11775	Cadherin-2	32/2	1/1
C2_6942	Calcineurin subunit B type 1	66/7.3	6/7
C2_67354	Calcium/calmodulin-dependent protein kinase type II delta 1 chain	122/20.1	3/7
C2_54503	Calcium/calmodulin-dependent protein kinase type II delta chain	79/16	4/5
C2_22563	Calcium/calmodulin-dependent protein kinase type II subunit gamma	76/17.4	6/9
C2_96091	Calcium-binding mitochondrial carrier protein SCaMC-2	156/4.2	1/15
C2_6728	Calcium-binding protein 39	76/10.9	8/10
C2_3736	Calcium-binding protein 39-like	62/15.7	9/10
C2_1929	Calcium-binding protein p22	48/6.1	4/5
AM952865	Calcium-regulated heat stable protein 1	109/6.9	1/2
C2_23310	Caldesmon	39/4.7	2/3
C2_103184	Calmodulin	36/11.1	2/2
C2_19770	Calnexin	289/14.6	12/18
C2_5497	Calpain small subunit 1	1440/40.7	20/50
C2_15607	Calpain-1 catalytic subunit	546/34	13/29
C2_4520	Calpain-2 catalytic subunit	2219/48.4	30/105
C2_55335	Calpain-5	61/14	4/20
C2_44459	Calpain-8	521/54.3	10/34
C2_78506	Calpain-9	232/24.2	11/15
C2_12340	Calpastatin	1687/24.3	18/62

C2_1782	Calponin-2	77/8	6/11
C2_1023	Calreticulin	298/25.4	16/22
C2_7975	Calumenin-A	44/3.7	2/9
C2_15813	cAMP-dependent protein kinase catalytic subunit alpha	44/3.2	2/2
C2_11147	cAMP-dependent protein kinase catalytic subunit beta	44/4	3/3
C2_12306	cAMP-dependent protein kinase inhibitor alpha	38/5.8	5/6
C2_4209	cAMP-regulated phosphoprotein 19-A	37/4.7	4/5
C2_27747	cAMP-responsive element-binding protein-like 2	40/4.8	3/8
C2_20033	Canalicular multispecific organic anion transporter 2	100/11.5	3/4
C2_9111	CAP-Gly domain-containing linker protein 1	33/6.2	5/5
C2_474	Carbonic anhydrase	63/7	5/5
C2_1891	Carbonic anhydrase 1	80/8	6/8
C2_85559	Carbonic anhydrase 2	83/18.1	2/2
C2_15533	Carbonic anhydrase 5B, mitochondrial	66/9.4	6/6
C2_14726	Carbonyl reductase [NADPH] 1	1035/45	16/52
C2_1812	Carboxymethylenebutenolidase homolog	113/14.3	6/9
C2_79368	Carboxypeptidase N subunit 2	45/3.4	1/2
C2_7894	Carboxy-terminal domain RNA polymerase II polypeptide A small phosphatase 1	46/8.3	13/15
C2_22283	Cardiomyopathy-associated protein 5	41/2.2	1/4
C2_4920	Carnitine O-palmitoyltransferase 1, liver isoform	41/2.9	3/7
C2_2166	Carnitine O-palmitoyltransferase 2, mitochondrial	209/7.6	5/9
C2_599	Cartilage intermediate layer protein 1	45/6.6	8/10
C2_7099	Casein kinase I isoform alpha	258/20.1	13/21
C2_540	Casein kinase II subunit alpha	91/6.9	4/4
C2_8203	Casein kinase II subunit beta	37/2.7	2/2
C2_16670	Caspase 1	59/5.4	2/3
C2_3535	Caspase 3	57/6.1	4/6
C2_5709	Caspase 6	226/21.4	11/16
C2_13472	Catenin alpha-1	476/36.2	10/17
C2_13521	Catenin alpha-2	54/8	2/2
C2_25912	Catenin beta-1	136/9.4	5/8
C2_23957	Catenin delta-1	82/7.1	4/7
C2_1477	Cathepsin B	86/10	6/11
C2_6658	Cathepsin L1 (Fragments)	74/7.1	3/5
C2_1656	Cathepsin S	55/9.8	4/4
C2_1045	Cathepsin Z	172/7.8	5/8
C2_6469	C-C chemokine receptor type 1	38/2.1	2/3
C2_7739	CCR4-NOT transcription complex subunit 1	44/4.7	4/7
C2_9606	CD2-associated protein	52/7.2	3/7
C2_5205	CD8 alpha chain	52/8.2	5/9
C2_1400	CD81 antigen	38/2.5	3/3
C2_14646	CD81 protein	34/2.3	1/3
C2_924	CD9 antigen	132/12.3	7/8
C2_16241	Cdc42 effector protein 2	41/3.8	4/6
C2_34748	Cdc42-interacting protein 4 homolog	36/3.6	1/1
C2_211	CDGSH iron-sulfur domain-containing protein 1	90/12.6	6/6
C2_7498	CDGSH iron-sulfur domain-containing protein 2A	89/5.1	2/3
C2_66058	CDK5 regulatory subunit-associated protein 2	69/5.7	2/9
C2_6264	CDK5 regulatory subunit-associated protein 3	43/5.4	4/11
C2_2412	Cell differentiation protein RCD1 homolog	99/6.7	5/7
C2_99597	Cell division control protein 42	441/16.4	3/14
C2_1548	Cell division control protein 42 homolog	213/6.8	12/26
C2_10332	Cell division cycle and apoptosis regulator protein 1	38/8.3	5/9
C2_1942	Cell division cycle-associated protein 7	93/5.6	8/8

C2_4779	Cellular nucleic acid-binding protein	229/17.2	7/7
C2_71833	Centrin-2	45/15.3	3/4
C2_6744	Centromere protein N	42/6	4/7
C2_15711	Centrosomal protein of 135 kDa	59/3	2/2
C2_5586	Ceramide synthase 5	30/2.7	2/2
C2_39986	Ceruloplasmin	34/16	4/4
C2_10174	Charged multivesicular body protein 1a	51/17.6	11/17
C2_520	Charged multivesicular body protein 1b	42/10.3	6/7
C2_11268	Charged multivesicular body protein 4c	56/2.4	1/1
C2_3579	Charged multivesicular body protein 5	31/2.3	1/1
C2_1563	Chitinase domain-containing protein 1	40/3.4	2/2
C2_3698	Chloride anion exchanger	54/4.2	4/5
C2_1437	Chloride intracellular channel protein 4	212/13.3	16/20
C2_6244	Choline transporter-like protein 2	65/5.3	5/5
C2_31948	Choline-phosphate cytidylyltransferase A	46/8.4	3/4
C2_19346	Chromatin-remodeling complex ATPase chain isw-1	48/2.4	2/4
C2_9123	Chromosome partition protein Smc	42/12.1	5/8
C2_19864	Cingulin	66/13.3	6/10
C2_2740	Citrate synthase, mitochondrial	405/8.1	10/30
C2_13187	Clathrin heavy chain 1	1700/63	22/60
C2_7312	Claudin-4	265/9.3	4/12
C2_3998	Claudin-7-B	134/4.7	2/6
C2_14161	Cleavage and polyadenylation specificity factor subunit 1	61/3.9	3/10
C2_1094	Cleavage and polyadenylation specificity factor subunit 5	50/5.7	3/5
C2_4149	Cleft lip and palate transmembrane protein 1 homolog	33/7.1	7/8
C2_8731	CLIP-associating protein 1	34/8.9	9/10
C2_76527	Coactosin-like protein	195/21.7	6/11
C2_80997	Coagulation factor XIII A chain	40/5.4	1/3
C2_5592	Coatomer subunit alpha	630/24.8	13/31
C2_4111	Coatomer subunit beta	916/34.7	24/60
C2_6399	Coatomer subunit beta'	259/16.6	15/28
C2_5687	Coatomer subunit delta	468/19	20/42
C2_191	Coatomer subunit epsilon	141/10.2	7/7
C2_3555	Coatomer subunit gamma-2	1617/35.8	27/68
C2_157	Coatomer subunit zeta-1	140/14.9	6/6
C2_1192	Cofilin-2	209/10.8	3/4
C2_3194	Cohesin subunit SA-2	40/4.1	8/9
C2_682	Coiled-coil domain-containing protein 109B	241/18.6	7/15
C2_31622	Coiled-coil domain-containing protein 157	39/15.2	5/11
C2_7339	Coiled-coil domain-containing protein 25	61/3.6	1/1
C2_2941	Coiled-coil domain-containing protein 56	68/8.7	3/3
C2_26160	Coiled-coil domain-containing protein 75	35/7.9	3/4
C2_88178	Coiled-coil domain-containing protein 97	56/6.3	2/3
C2_2709	Coiled-coil-helix-coiled-coil-helix domain-containing protein 1	44/6.3	2/10
C2_4483	Cold-inducible RNA-binding protein A	127/10.5	5/6
C2_3464	Cold-inducible RNA-binding protein B	118/6.6	3/4
C2_389	Collagen alpha-1(I) chain	104/10.7	6/6
C2_5595	Collagen alpha-1(VI) chain	37/5.6	3/4
C2_14462	Collagen alpha-1(X) chain	56/4.1	3/21
C2_2286	Collagen alpha-1(XXV) chain	199/4.6	2/11
C2_654	Collagen alpha-2(I) chain	54/4	4/9
C2_37905	Collagen alpha-3(VI) chain	351/27	6/20
C2_6735	Collagen type IV alpha-3-binding protein	40/1.4	2/8
C2_15503	Collectin-12	45/15.4	4/5
C2_1725	COMM domain-containing protein 7	38/3	1/3

C2_26115	Complement C1q subcomponent subunit A	30/11.6	5/5
C2_107825	Complement C1q-like protein 2	41/6	4/6
FP333165	Complement C2	38/6	2/3
C2_1398	Complement C3	1057/29.8	53/102
C2_52123	Complement C5	76/15.1	2/2
C2_2093	Complement component C6	37/4.9	8/11
s_rl0001d01_f_1	Complement component C9	49/7.7	1/1
C2_9760	Complement factor B	85/5.6	3/5
C2_2374	Complement factor H	140/12.2	10/10
C2_8711	Conserved oligomeric Golgi complex subunit 2	41/3.4	3/3
C2_754	Conserved oligomeric Golgi complex subunit 5	43/5.3	3/3
C2_10848	Conserved oligomeric Golgi complex subunit 6	37/5	3/3
C2_19596	Conserved oligomeric Golgi complex subunit 7	53/5.3	3/7
C2_26344	Constitutive coactivator of PPAR-gamma-like protein 1 homolog	46/10	7/10
C2_1232	COP9 signalosome complex subunit 1	47/5.3	4/4
C2_1948	COP9 signalosome complex subunit 3	35/2.3	2/5
C2_1203	COP9 signalosome complex subunit 5	43/6.4	3/5
C2_3742	COP9 signalosome complex subunit 6	88/12.6	5/5
C2_3473	COP9 signalosome complex subunit 7a	83/14.3	4/6
C2_2034	COP9 signalosome complex subunit 8	82/14.9	5/5
C2_6548	Copine-1	435/35.1	12/31
C2_2684	Copine-3	215/10.6	12/19
C2_3222	Copper homeostasis protein cutC homolog	77/4.7	2/4
C2_23990	Copper transport protein ATOX1	43/11.3	6/10
C2_2145	Core histone macro-H2A.1	120/12.1	5/9
C2_3803	Core histone macro-H2A.2	126/15.3	10/13
C2_451	Coronin-1A	257/18.5	10/16
C2_5290	Coronin-1C	1026/17.1	28/57
C2_5573	Coronin-7	158/10.9	10/16
C2_2727	Costars family protein C6orf115 homolog	88/3.9	4/5
C2_16210	Counting factor associated protein D	68/4.5	3/10
C2_3162	COX assembly mitochondrial protein homolog	57/9.1	4/21
C2_59873	CREB-regulated transcription coactivator 1	37/5.6	2/4
C2_1078	Crystallin J1C	119/9.3	4/4
C2_4406	Cullin-3	42/2.5	2/3
C2_85398	Cullin-associated NEDD8-dissociated protein 1	155/44.2	6/7
C2_58682	CXADR-like membrane protein	80/6.7	4/21
C2_94	Cyclin-G1	31/3.6	5/11
C2_77236	Cyclin-Y	36/13.4	2/3
C2_1820	Cystathionine beta-synthase	82/6.7	5/7
C2_2620	Cystathionine gamma-lyase	72/8.2	4/5
C2_91	Cystatin	178/21.4	7/7
C2_16817	Cystatin-A1	46/7.5	1/1
C2_617	Cystatin-B	522/24	5/17
C2_8210	Cysteine and glycine-rich protein 1	261/28.1	9/19
C2_42628	Cysteine-rich protein 1	43/18.3	6/6
C2_4479	Cysteinyl-tRNA synthetase, cytoplasmic	220/18.5	12/13
C2_424	Cystinosin	35/4.7	4/7
C2_26811	Cytochrome c iso-1/iso-2	121/21.8	4/5
C2_5715	Cytochrome c oxidase subunit 3	52/14.2	4/5
C2_270	Cytochrome c oxidase subunit 4 isoform 1, mitochondrial	200/17.6	3/9
C2_462	Cytochrome c oxidase subunit 4 isoform 2, mitochondrial	42/8.7	4/4
C2_238	Cytochrome c oxidase subunit 5A, mitochondrial	51/7.8	4/5
C2_16418	Cytochrome c oxidase subunit 6A, mitochondrial	38/13.8	2/2
C2_1197	Cytochrome c oxidase subunit 6B1	31/10.7	2/4

C2_2923	Cytochrome c oxidase subunit 6C-1	134/23.4	4/7
C2_3512	Cytochrome c oxidase subunit 7B, mitochondrial	35/11.7	2/2
C2_785	Cytochrome c1, heme protein, mitochondrial	189/22.3	10/16
C2_1778	Cytochrome c-b	148/8.1	4/4
C2_4087	Cytochrome P450 2J2	40/5.5	10/12
C2_2545	Cytolysin RTX-A	1585/60.8	15/64
C2_1467	Cytolysin Src-1	2316/38.4	12/70
C2_5034	Cytoplasmic dynein 1 heavy chain 1	943/41.3	32/49
C2_7060	Cytoplasmic dynein 1 intermediate chain 2	251/15.2	12/13
C2_2521	Cytoplasmic dynein 1 light intermediate chain 2	40/7.5	7/11
C2_91059	Cytoplasmic dynein 2 heavy chain 1	41/1.8	1/1
C2_11304	Cytoplasmic FMR1-interacting protein 1 homolog	638/27.6	14/39
C2_33608	Cytoplasmic FMR1-interacting protein 2	114/10	4/8
C2_7146	Cytosol aminopeptidase	40/8.9	7/12
C2_13504	Cytosolic 5'-nucleotidase 1B	46/10.5	5/10
C2_17093	Cytosolic carboxypeptidase 1	40/3	2/14
C2_14346	Cytosolic carboxypeptidase-like protein 5	33/1.8	1/1
C2_928	Cytosolic non-specific dipeptidase	251/27.3	13/19
C2_7071	Cytosolic phospholipase A2	37/9.2	9/21
C2_32889	Cytosolic phospholipase A2 gamma	107/18.4	3/5
C2_8292	D-3-phosphoglycerate dehydrogenase	413/31	18/24
C2_39429	DDB1- and CUL4-associated factor 6	35/4.7	1/2
C2_1763	D-dopachrome decarboxylase	86/2.7	2/8
C2_3243	Death ligand signal enhancer	53/6	5/7
C2_66882	Death-associated protein-like 1	41/12.8	2/3
C2_40372	Dedicator of cytokinesis protein 10	32/6.7	3/10
C2_21293	Dedicator of cytokinesis protein 7	35/7	6/15
C2_4594	Dehydrogenase/reductase SDR family member 11	149/19.4	6/7
C2_35778	Dehydrogenase/reductase SDR family member 12	68/2.3	1/1
C2_14343	Dehydrogenase/reductase SDR family member 4	50/4.8	1/1
C2_12746	Delta(24)-sterol reductase	30/3.7	2/2
C2_17169	Delta-aminolevulinic acid dehydratase	37/5.9	3/3
C2_41796	DENN domain-containing protein 1C	130/13.8	4/17
C2_734	DENN domain-containing protein 2D	38/5.8	5/10
C2_8532	Deoxycytidylate deaminase	82/4.6	4/6
C2_17301	Derlin-1	68/4.2	1/2
C2_14136	Desmocollin-2	188/13.1	12/16
C2_26247	Desmoglein-2	75/11.8	3/5
C2_20476	Desmoplakin	674/27.2	18/49
C2_521	Diablo homolog, mitochondrial	36/6.1	2/3
C2_6037	Differentially expressed in FDCP 6 homolog	36/4.4	4/13
C2_5593	Dihydrolipoyl dehydrogenase, mitochondrial	451/20.2	18/28
C2_11248	Dihydrolipoyllysine-residue acetyltransferase component of pyruvate dehydrogenase complex, mitochondrial	110/6.9	5/8
C2_1668	Dihydrolipoyllysine-residue succinyltransferase component of 2-oxoglutarate dehydrogenase complex, mitochondrial	78/11.4	9/15
C2_9692	Dihydrolipoyllysine-residue succinyltransferase component of 2-oxoglutarate dehydrogenase complex, mitochondrial	75/5.9	6/10
C2_192	Dipeptidase 1	38/5	4/5
C2_366	Dipeptidyl peptidase 1	149/5.7	5/8
C2_14794	Dipeptidyl peptidase 3	150/9.7	3/5
C2_6203	Dipeptidyl peptidase 9	140/9.8	8/10
C2_3656	Diphosphoinositol polyphosphate phosphohydrolase 2	33/4.4	4/8
C2_24677	Disintegrin and metalloproteinase domain-containing protein 10	64/8.6	3/4
C2_39959	Disintegrin and metalloproteinase domain-containing protein 17	31/8.1	2/5

C2_10283	Disks large homolog 2	70/5.5	6/7
C2_728	DLA class II histocompatibility antigen, DR-1 beta chain	356/18.8	7/17
C2_15432	DNA damage-binding protein 1	68/12.1	3/4
C2_13486	DNA damage-regulated autophagy modulator protein 2	46/6.4	4/6
C2_9250	DNA excision repair protein ERCC-1	39/4.9	3/6
C2_9234	DNA ligase 1	42/10.9	5/8
C2_2773	DNA polymerase beta	46/7.6	6/10
C2_26637	DNA polymerase kappa	34/7.8	2/4
C2_12924	DNA polymerase zeta catalytic subunit	42/5.9	4/5
C2_11250	DNA repair protein XRCC4	36/8.7	9/14
C2_2056	DNA-(apurinic or apyrimidinic site) lyase	34/5.1	2/2
C2_3639	DNA-directed RNA polymerase II subunit RPB1	39/2	2/4
C2_4769	DNA-directed RNA polymerase II subunit RPB2	36/2.6	3/3
C2_7938	DNA-directed RNA polymerase II subunit RPB9	67/9.8	1/1
C2_6701	DNA-directed RNA polymerases I, II, and III subunit RPABC3	47/12.8	3/4
C2_213	DnaJ homolog subfamily A member 1	332/9	9/37
C2_5322	DnaJ homolog subfamily C member 17	39/9.2	4/4
C2_8665	DnaJ homolog subfamily C member 22	40/2.5	2/2
C2_6745	Dolichol-phosphate mannosyltransferase	41/7.4	5/8
C2_1240	Dolichyl-diphosphooligosaccharide--protein glycosyltransferase 48 kDa subunit	213/21.3	12/17
C2_3023	Dolichyl-diphosphooligosaccharide--protein glycosyltransferase subunit 1	772/27	20/46
C2_1591	Dolichyl-diphosphooligosaccharide--protein glycosyltransferase subunit 2	909/18.8	10/22
C2_120875	Dolichyl-diphosphooligosaccharide--protein glycosyltransferase subunit DAD1	151/9.7	2/5
C2_1500	Dolichyl-diphosphooligosaccharide--protein glycosyltransferase subunit STT3A	316/17.8	17/20
C2_7853	Dolichyl-diphosphooligosaccharide--protein glycosyltransferase subunit STT3B	156/10.3	7/7
C2_3570	Dolichyl-phosphate beta-glucosyltransferase	127/8.6	11/21
C2_13975	Dr1-associated corepressor	47/4.8	5/12
C2_6436	Drebrin-like protein	39/6.6	5/8
C2_8403	Drebrin-like protein B	47/7.9	4/8
C2_24867	Dual specificity mitogen-activated protein kinase kinase 6	35/14.1	5/6
C2_8517	Dual specificity protein phosphatase 1	154/9.4	6/7
C2_1808	Dual specificity protein phosphatase 23	39/6.1	6/6
C2_58934	Dynactin subunit 1	66/14.3	2/3
C2_6275	Dynamin-2	111/16.3	15/16
C2_27580	Dynein light chain 2, cytoplasmic	289/28.2	8/13
C2_1293	Dynein light chain roadblock-type 1	61/9.7	4/9
C2_1352	Dynein light chain Tctex-type 3	47/5	2/3
C2_6118	E3 ubiquitin/ISG15 ligase TRIM25	37/7.5	7/14
C2_3233	E3 ubiquitin-protein ligase AMFR	38/2.7	4/4
C2_13765	E3 ubiquitin-protein ligase arih1l	34/5.5	6/7
C2_8265	E3 ubiquitin-protein ligase BRE1B	40/7	5/15
C2_73398	E3 ubiquitin-protein ligase HUWE1	87/19.2	2/2
C2_3451	E3 ubiquitin-protein ligase RNF128	34/5.6	7/16
C2_22755	E3 ubiquitin-protein ligase RNF168	37/7.8	2/2
C2_6373	E3 ubiquitin-protein ligase RNF8	41/7.1	5/9
C2_104308	E3 ubiquitin-protein ligase TRIM17	56/10.5	4/7
C2_86311	E3 ubiquitin-protein ligase UBR3	41/6.8	1/3
C2_2829	E3 ubiquitin-protein ligase UBR4	93/7.4	10/14
C2_4136	Echinoderm microtubule-associated protein-like 1	504/22.4	26/44

C2_21215	Echinoderm microtubule-associated protein-like 4	61/15.1	4/4
C2_97688	Echinoderm microtubule-associated protein-like 5	46/3.8	1/4
C2_2589	Ectonucleotide pyrophosphatase/phosphodiesterase family member 6	39/6.6	4/12
C2_8820	EF-hand domain-containing protein D2	440/24.4	9/16
C2_25319	EH domain-containing protein 1	86/30.3	8/8
C2_127	Electron transfer flavoprotein subunit alpha, mitochondrial	311/37.7	18/25
C2_1233	Electron transfer flavoprotein subunit beta	275/16.1	8/12
C2_2103	Electron transfer flavoprotein-ubiquinone oxidoreductase, mitochondrial	74/5.8	10/40
C2_18	Elongation factor 1-alpha	2513/43.4	26/151
C2_9057	Elongation factor 1-alpha 2	191/12.4	6/26
C2_35612	Elongation factor 1-alpha, somatic form	1016/42.4	5/42
C2_344	Elongation factor 1-beta	231/42.2	12/26
C2_2938	Elongation factor 1-delta	287/24	10/19
C2_55	Elongation factor 1-gamma	851/36.4	19/70
C2_534	Elongation factor 2	2354/49.8	36/139
C2_4121	Elongation factor Ts, mitochondrial	46/5.8	2/2
C2_63003	Elongation of very long chain fatty acids protein 7	35/9.3	1/1
C2_3632	Embryonic polyadenylate-binding protein A	177/12.4	8/13
C2_9305	Endophilin-A1	39/6.5	4/6
C2_11409	Endoplasmic reticulum aminopeptidase 1	327/22.5	10/16
C2_26383	Endoplasmic reticulum metallopeptidase 1	52/5.6	2/3
C2_21801	Endoplasmic reticulum resident protein 44	61/10.2	2/2
C2_17050	Endoplasmin	48/5.3	3/4
C2_1490	Endoplasmin (GRP-94)	536/21.7	22/33
C2_1311	Endothelial differentiation-related factor 1 homolog	42/10.1	5/5
C2_13119	Enolase	376/47.7	9/16
C2_22342	Enolase-phosphatase E1	100/19.8	5/5
C2_5154	Enoyl-CoA delta isomerase 1, mitochondrial	105/12.7	7/11
C2_64284	Enoyl-CoA hydratase domain-containing protein 2, mitochondrial	73/15.6	4/6
C2_3722	Enoyl-CoA hydratase, mitochondrial	277/30	13/19
C2_646	Eosinophil peroxidase	101/10	10/14
C2_24789	Ephexin-1	99/3.2	2/6
C2_16499	Ephrin type-A receptor 3	38/4.2	3/10
C2_5498	Epidermal growth factor receptor kinase substrate 8-like protein 3	115/7.3	9/11
C2_30021	Epidermis-type lipoxygenase 3	64/13.3	10/19
C2_782	Epididymal secretory protein E1	145/20.7	6/6
C2_20845	Epiplakin	69/26.8	6/8
C2_22855	Epoxide hydrolase 2	47/4.7	2/7
C2_4	Equilibrative nucleoside transporter 3	4214/8.2	20/168
C2_37202	Equistatin	31/6.1	3/8
C2_21291	ERI1 exoribonuclease 2	41/4.4	4/7
C2_16588	Erlin-2	33/7.1	5/5
C2_1732	Erythrocyte band 7 integral membrane protein	42/11	5/5
C2_800	ES1 protein homolog, mitochondrial	123/14.2	6/6
AM954774	Estradiol 17-beta-dehydrogenase 12-A	360/26.8	7/14
C2_12674	Estradiol 17-beta-dehydrogenase 12-B	46/4.1	3/4
C2_48308	Estrogen receptor	34/4.7	7/8
C2_406	Eukaryotic initiation factor 4A-I	1474/13.9	18/64
C2_746	Eukaryotic initiation factor 4A-II	709/14.3	8/33
C2_1414	Eukaryotic initiation factor 4A-III	214/7.7	5/9
C2_3344	Eukaryotic peptide chain release factor GTP-binding subunit ERF3A	112/11.5	6/15
C2_569	Eukaryotic peptide chain release factor subunit 1	68/5.4	5/7
C2_3415	Eukaryotic translation elongation factor 1 epsilon-1	79/9.3	7/7
C2_25618	Eukaryotic translation initiation factor 1A, X-chromosomal	68/14	3/4
C2_4011	Eukaryotic translation initiation factor 2 subunit 1	86/12.6	6/8

C2_5966	Eukaryotic translation initiation factor 2 subunit 2	88/11	9/14
C2_1614	Eukaryotic translation initiation factor 2 subunit 3	146/22.7	13/17
C2_533	Eukaryotic translation initiation factor 3 subunit A	240/14	13/25
C2_630	Eukaryotic translation initiation factor 3 subunit B	218/17.5	13/16
C2_114	Eukaryotic translation initiation factor 3 subunit E	42/11.4	7/13
C2_92	Eukaryotic translation initiation factor 3 subunit F	175/16.5	6/8
C2_1542	Eukaryotic translation initiation factor 3 subunit G	33/18.3	5/6
C2_312	Eukaryotic translation initiation factor 3 subunit I	195/19.6	11/13
C2_5501	Eukaryotic translation initiation factor 3 subunit K	172/36.8	9/13
C2_372	Eukaryotic translation initiation factor 3 subunit L	497/29.2	20/33
C2_68	Eukaryotic translation initiation factor 3 subunit M	165/13.3	5/8
C2_2506	Eukaryotic translation initiation factor 4 gamma 1	44/2.5	2/2
C2_1810	Eukaryotic translation initiation factor 4E	53/4.4	3/3
C2_2581	Eukaryotic translation initiation factor 4E-binding protein 2	48/5.4	5/15
C2_41590	Eukaryotic translation initiation factor 4H	136/16.1	4/6
C2_3882	Eukaryotic translation initiation factor 5A-1	119/8.9	8/11
C2_37824	Eukaryotic translation initiation factor 5A-2	186/10.9	4/8
C2_6319	Eukaryotic translation initiation factor 6	181/9.1	3/7
C2_2647	Exocyst complex component 3	43/9	9/10
C2_7782	Exocyst complex component 5	43/5.4	7/7
C2_54734	Exportin-1	64/8.6	1/1
C2_6241	Exportin-2	59/7	7/9
C2_56695	Exportin-7	36/3.7	1/2
C2_48857	Extended synaptotagmin-2-B	121/19.8	3/4
C2_8464	Extracellular superoxide dismutase [Cu-Zn]	35/4	4/5
C2_57820	F-actin-capping protein subunit alpha	123/14.9	3/7
C2_356	F-actin-capping protein subunit alpha-1	304/17.5	9/34
C2_207	F-actin-capping protein subunit alpha-2	178/14.5	3/5
C2_227	F-actin-capping protein subunit beta	560/25.7	19/42
C2_48653	Far upstream element-binding protein 1	37/5.6	3/4
C2_17942	Far upstream element-binding protein 3	44/7.3	4/5
C2_20701	Fas apoptotic inhibitory molecule 2	39/4	1/5
C2_32600	Fascin	35/3.6	1/1
C2_56579	Fatty acid synthase	165/16.4	5/7
C2_168	Fatty acid-binding protein, heart	287/23.9	9/13
C2_15334	F-box only protein 18	42/5.8	3/8
C2_10084	F-box only protein 3	134/1.9	2/19
C2_53257	F-box/WD repeat-containing protein 11	45/11.3	2/6
C2_5555	FCH domain only protein 2	79/6.1	13/40
C2_3523	Ferric-chelate reductase 1	37/5	5/18
C2_22101	Ferritin heavy chain	112/20.1	3/4
C2_105435	Ferritin light chain, oocyte isoform	36/8.2	1/1
C2_1658	Ferritin, heavy subunit	151/12.5	4/5
C2_101117	Ferritin, middle subunit	74/11.8	5/8
FP331537	Fibrinogen alpha chain	137/18.4	4/8
s_rlp0007c05_f_1	Fibrinogen beta chain	91/5.6	1/2
FP332283	Fibrinogen beta chain	91/53.5	4/5
C2_38689	Fibrinogen gamma chain	169/16.1	6/11
FM149950	Fibroblast growth factor-binding protein 1	39/13.3	3/4
C2_58470	Fibronectin	70/28.3	2/2
C2_66317	Fidgetin-like protein 1	60/6.6	2/4
C2_6274	Filamin-A	506/29.8	21/30
C2_79807	Filamin-B	179/25.9	3/10
C2_22478	Filamin-C	358/29	9/14
C2_2848	Flap endonuclease 1	38/3.9	2/5

C2_1227	Flavin reductase (NADPH)	62/12.3	11/14
C2_8431	Flocculation protein FLO11	57/2.3	3/5
C2_1217	Flotillin-1	558/16.7	17/33
C2_3325	Flotillin-2a	404/10.8	16/32
C2_15353	Folliculin-interacting protein 1	39/4.5	3/6
C2_49308	Formin-binding protein 1 homolog	53/7.1	3/9
C2_27579	Fragile X mental retardation syndrome-related protein 1 homolog B	41/6.5	3/5
C2_6314	Fragile X mental retardation syndrome-related protein 2	52/5	4/11
C2_38182	FRAS1-related extracellular matrix protein 1	33/6.5	4/9
C2_90759	Frizzled-2	73/6.3	1/6
C2_519	Fructose-bisphosphate aldolase A	155/14.8	5/18
C2_185	Fructose-bisphosphate aldolase B	463/28.1	12/32
C2_4027	Fructose-bisphosphate aldolase C	203/28.1	4/15
C2_1285	Fructose-bisphosphate aldolase C-B	263/11.4	9/16
C2_23658	Fructose-bisphosphate aldolase, muscle type	273/28.6	3/12
C2_32246	Fucolectin	190/7.3	4/22
C2_3057	Fucolectin-4	151/7.6	3/20
C2_1866	Fucose mutarotase	67/4.9	2/2
C2_5086	Fumarate hydratase, mitochondrial	153/13.4	10/13
C2_3348	Fumarylacetoacetase	35/4.9	3/4
C2_24896	Fumarylacetoacetate hydrolase domain-containing protein 1	120/9.4	2/2
C2_1568	Fumarylacetoacetate hydrolase domain-containing protein 2	382/22.3	14/22
C2_2065	FUN14 domain-containing protein 1	53/2.6	1/2
C2_395	FXYD domain-containing ion transport regulator 3	99/4.2	5/7
C2_20462	FYVE and coiled-coil domain-containing protein 1	39/4.6	4/7
C2_2035	Galactocerebrosidase	82/12.5	8/8
C2_28569	Galectin-3	816/17.2	8/28
AM954422	Gamma-glutamylaminecyclotransferase A	30/8.8	3/5
C2_63	Gamma-interferon-inducible lysosomal thiol reductase	110/5	9/10
C2_2606	Ganglioside GM2 activator	50/6.2	9/15
C2_8810	Gastrotropin	101/15.3	4/6
C2_73371	GDP-fucose transporter 1	35/10.2	3/3
C2_8486	GDP-L-fucose synthase	91/7.7	9/9
C2_3719	GDP-mannose 4,6 dehydratase	108/13	7/8
C2_265	Gelsolin	3503/41.2	41/156
C2_5479	General transcription factor IIH subunit 2	37/5.5	3/13
C2_3592	Geranylgeranyl pyrophosphate synthase	36/6.3	5/5
C2_36790	Glia maturation factor gamma	104/14.3	3/6
C2_4961	Glucocorticoid receptor	35/3.2	7/8
C2_62435	Glucokinase	38/2.3	2/2
C2_2386	Glucosamine 6-phosphate N-acetyltransferase	129/9.1	6/6
C2_768	Glucosamine-6-phosphate isomerase 1	52/9.5	4/7
C2_8719	Glucosamine--fructose-6-phosphate aminotransferase [isomerizing] 1	508/16.8	17/42
C2_20259	Glucosamine--fructose-6-phosphate aminotransferase [isomerizing] 2	987/46.4	15/41
C2_5943	Glucose-6-phosphate 1-dehydrogenase	105/10.9	6/9
C2_394	Glucose-6-phosphate isomerase	948/36.5	18/39
C2_3924	Glutamate dehydrogenase, mitochondrial	654/19.4	17/44
C2_15470	Glutamine-rich protein 1	54/1.5	2/4
C2_9383	Glutaminyl-peptide cyclotransferase	33/2.4	1/1
C2_2268	Glutaminyl-tRNA synthetase	83/17.4	17/17
C2_46090	Glutaredoxin	40/10.4	3/3
C2_22341	Glutaredoxin-related protein 5, mitochondrial	33/7.3	1/1
C2_345	Glutathione peroxidase 1	228/25.7	14/23
C2_19719	Glutathione reductase, mitochondrial	45/7.5	4/4
C2_5598	Glutathione S-transferase 3	515/27.7	14/42

C2_117130	Glutathione S-transferase A	606/52.8	18/49
C2_112663	Glutathione S-transferase A4	321/19.7	8/23
C2_112228	Glutathione S-transferase alpha M14	116/23.1	5/8
C2_430	Glutathione S-transferase kappa 1	54/9.5	5/5
C2_868	Glutathione S-transferase Mu 3	322/31.7	10/14
C2_1660	Glutathione S-transferase theta-1	175/15.5	11/22
C2_29	Glyceraldehyde 3-phosphate dehydrogenase, testis-specific	5529/49.5	26/184
C2_17	Glyceraldehyde-3-phosphate dehydrogenase	127/9.8	6/27
C2_30748	Glyceraldehyde-3-phosphate dehydrogenase 1	112/8.9	4/24
C2_2813	Glycerol-3-phosphate dehydrogenase 1-like protein	393/37.5	19/32
C2_27493	Glycine N-methyltransferase	263/35.2	13/17
C2_8433	Glycine N-methyltransferase	145/15.9	13/17
C2_2473	Glycogen [starch] synthase, muscle	131/8.5	11/16
C2_15427	Glycogen debranching enzyme	212/22.8	10/17
C2_75238	Glycogen phosphorylase, brain form	70/11.6	2/2
C2_4003	Glycogen phosphorylase, liver form	2575/41.4	42/133
C2_41881	Glycogen phosphorylase, muscle form	268/9	3/9
C2_1791	Glycogenin-1	212/15.5	9/11
C2_1350	Glycolipid transfer protein	55/6.4	3/3
C2_64886	Glycoprotein endo-alpha-1,2-mannosidase-like protein	36/16.6	2/8
C2_637	Glycylpeptide N-tetradecanoyltransferase 1	57/4.6	3/3
C2_1726	Glycyl-tRNA synthetase	553/21	17/25
C2_2114	Glyoxalase domain-containing protein 4	67/6.8	5/6
C2_2680	Glyoxalase domain-containing protein 5	86/14.9	5/7
C2_111769	Glyoxylate/hydroxypyruvate reductase B	113/19.1	3/9
C2_17359	GMP reductase 1	103/8.9	3/7
C2_55209	GMP reductase 2	73/8.3	1/1
C2_1539	GMP synthase [glutamine-hydrolyzing]	121/4.4	6/10
C2_8843	Golgi apparatus protein 1	115/12.2	11/13
C2_95316	Golgi phosphoprotein 3	97/10.8	4/8
C2_27025	Golgin subfamily A member 4	54/7	3/4
C2_3346	GPI mannosyltransferase 3	35/5.2	8/9
C2_16081	GPN-loop GTPase 1	47/6.8	5/10
C2_3971	Grancalcin	180/9	5/6
C2_1586	Granulins	41/4	5/5
C2_4386	Granulins	34/1.4	1/6
C2_1707	Growth arrest and DNA damage-inducible protein GADD45 alpha	35/4.8	3/3
C2_4717	Growth factor receptor-bound protein 2	39/4.5	3/3
C2_836	Growth inhibition and differentiation-related protein 88 homolog	45/5.9	6/7
C2_1926	GrpE protein homolog 1, mitochondrial	41/8.8	4/5
C2_1191	GTP:AMP phosphotransferase, mitochondrial	62/4.2	4/4
C2_80217	GTPase IMAP family member 2	50/5.3	2/2
C2_11768	GTPase IMAP family member 4	49/7.5	4/5
C2_5033	GTPase IMAP family member 7	53/3.4	2/7
C2_2112	GTPase KRas	172/22.9	7/10
C2_6491	GTPase SLIP-GC	35/3.7	4/5
C2_1141	GTP-binding nuclear protein Ran	806/30.9	11/34
C2_1272	GTP-binding protein SAR1b	246/14.1	10/12
C2_3738	Guanine nucleotide-binding protein G(i) subunit alpha-1	433/15	12/27
C2_64382	Guanine nucleotide-binding protein G(I)/G(S)/G(O) subunit gamma-12	76/16.3	2/2
C2_7512	Guanine nucleotide-binding protein G(I)/G(S)/G(O) subunit gamma-2	60/2.8	2/5
C2_8162	Guanine nucleotide-binding protein G(I)/G(S)/G(T) subunit beta-1	653/26.2	10/25
C2_41327	Guanine nucleotide-binding protein G(I)/G(S)/G(T) subunit beta-2	51/15.8	3/4
C2_6805	Guanine nucleotide-binding protein G(k) subunit alpha	409/12.4	10/26
C2_35672	Guanine nucleotide-binding protein G(t) subunit alpha-2	187/7	6/14

C2_75053	Guanine nucleotide-binding protein subunit alpha-11	43/18.1	5/8
C2_5820	Guanine nucleotide-binding protein subunit alpha-13	187/11.6	4/9
C2_45	Guanine nucleotide-binding protein subunit beta-2-like 1	1662/68.9	23/78
C2_3160	Guanine nucleotide-binding protein subunit beta-4	246/8	6/13
C2_1122	Guanine nucleotide-binding protein-like 3	47/4.5	3/5
C2_10055	Guanosine-3',5'-bis(diphosphate) 3'-pyrophosphohydrolase MESH1	58/11.7	4/4
C2_33360	H/ACA ribonucleoprotein complex subunit 2-like protein	82/18.5	6/7
C2_10333	H/ACA ribonucleoprotein complex subunit 3	36/8.9	2/2
C2_2205	H/ACA ribonucleoprotein complex subunit 4	233/16.7	8/15
C2_1165	H-2 class II histocompatibility antigen, A-Q beta chain	273/17	7/13
C2_104432	H-2 class II histocompatibility antigen, A-R alpha chain	225/9.5	4/8
C2_2861	H-2 class II histocompatibility antigen, A-S beta chain	270/17.9	5/10
C2_105193	H-2 class II histocompatibility antigen, E-D alpha chain	120/30.3	8/9
C2_100478	H-2 class II histocompatibility antigen, E-U alpha chain	94/11	4/5
C2_104583	H-2 class II histocompatibility antigen, I-E beta chain	340/18.3	7/17
C2_6599	HAUS augmin-like complex subunit 5	50/3.3	4/9
C2_4246	HBS1-like protein	45/4.9	6/13
C2_5365	HEAT repeat-containing protein 7A	41/2.9	2/4
C2_6606	Heat shock 70 kDa protein	1119/18.8	17/64
C2_15999	Heat shock 70 kDa protein 1	1215/20.5	18/62
C2_6720	Heat shock 70 kDa protein 4	56/8.6	7/8
C2_58	Heat shock cognate 70 kDa protein	3701/44.2	42/168
C2_4763	Heat shock cognate 71 kDa protein	2385/23	19/94
C2_2116	Heat shock protein 105 kDa	358/18.2	18/28
C2_10046	Heat shock protein beta-11	39/7.2	8/12
C2_4132	Heat shock protein HSP 90-alpha 1	737/15.4	15/41
C2_42	Heat shock protein HSP 90-beta	1787/40.9	40/102
C2_2149	Heme oxygenase	31/2.1	1/1
C2_16976	Hemoglobin anodic subunit alpha	138/13.7	2/4
C2_22936	Hemoglobin subunit alpha-A	2204/23.1	13/72
C2_121	Hemoglobin subunit alpha-B	1311/11.8	12/52
C2_6148	Hemoglobin subunit beta	6041/29	19/266
C2_27397	Hemopexin	276/20.9	12/15
C2_14772	Hepatoma-derived growth factor-related protein 2	40/7	5/10
C2_54989	Hermansky-Pudlak syndrome 3 protein	35/5.4	3/7
C2_30860	Heterochromatin protein 1-binding protein 3	48/9.4	2/2
C2_6512	Heterogeneous nuclear ribonucleoprotein A/B	136/11.2	6/13
C2_1787	Heterogeneous nuclear ribonucleoprotein A0	428/20.7	9/22
C2_24145	Heterogeneous nuclear ribonucleoprotein A1	435/29.9	8/18
C2_15328	Heterogeneous nuclear ribonucleoprotein C	74/8.3	11/14
C2_3241	Heterogeneous nuclear ribonucleoprotein H	52/7.8	4/4
C2_61488	Heterogeneous nuclear ribonucleoprotein K	140/13.2	2/4
C2_4888	Heterogeneous nuclear ribonucleoprotein L	55/4.2	3/3
C2_15127	Heterogeneous nuclear ribonucleoprotein M	48/11.5	4/11
C2_26519	Heterogeneous nuclear ribonucleoprotein Q	130/19.6	7/14
C2_46867	Heterogeneous nuclear ribonucleoprotein U	47/11.6	3/3
C2_1517	Heterogeneous nuclear ribonucleoprotein U-like protein 1	115/6.4	6/9
C2_75390	Hexokinase-1	71/11.7	3/3
C2_52031	Hexokinase-2	72/29.7	4/5
C2_8165	Hibernation-specific plasma protein HP-55	156/16.6	7/11
C2_13670	High affinity cGMP-specific 3',5'-cyclic phosphodiesterase 9A	48/5.3	4/6
C2_49345	High affinity immunoglobulin gamma Fc receptor I	60/5.1	2/2
C2_12834	High mobility group protein B1	130/18.2	5/8
C2_811	High mobility group protein B2	444/27.1	9/25
C2_25588	High mobility group protein B3	103/16.6	4/4

C2_2901	Histidine triad nucleotide-binding protein 1	185/40.5	5/6
C2_3982	Histidine triad nucleotide-binding protein 2, mitochondrial	337/25.8	7/14
C2_65292	Histidyl-tRNA synthetase, cytoplasmic	133/23.6	7/7
C2_121539	Histo-blood group ABO system transferase 1	39/13.9	2/3
C2_95666	Histo-blood group ABO system transferase 2	89/28	4/4
C2_458	Histone deacetylase 1	82/9.7	6/7
C2_31901	Histone deacetylase complex subunit SAP18	83/17.4	5/6
C2_11401	Histone deacetylase complex subunit SAP30L	37/4.4	4/5
C2_14306	Histone H1	589/17.6	9/25
C2_6291	Histone H1.03	463/24.9	8/36
C2_65	Histone H1.5	145/6.7	3/4
C2_12456	Histone H2A	586/21.4	10/43
C2_73474	Histone H2A type 1	552/23.9	7/28
C2_390	Histone H2A.V	505/12.2	8/28
C2_39438	Histone H2A.V	303/17.1	8/15
C2_47755	Histone H2B	1550/23.3	12/98
C2_101057	Histone H2B 1/2	1672/45.2	12/105
C2_1274	Histone H3.3	489/11.1	10/58
C2_19495	Histone H4	2109/37.1	14/99
C2_3745	Histone-binding protein RBBP4	58/9.5	5/7
C2_2830	Histone-binding protein RBBP7	39/4.3	4/5
C2_11055	Histone-lysine N-methyltransferase MLL	36/7.2	6/7
C2_14653	Histone-lysine N-methyltransferase MLL2	86/5.8	2/6
C2_7626	Histone-lysine N-methyltransferase MLL3	56/4.5	5/10
C2_15004	Histone-lysine N-methyltransferase SETD2	38/4.8	2/2
C2_32143	Histone-lysine N-methyltransferase SETDB2	40/10.1	5/7
C2_121377	HLA class II histocompatibility antigen, DP beta 1 chain	226/17.6	5/9
C2_113147	HLA class II histocompatibility antigen, DRB1-4 beta chain	342/18.5	6/16
C2_44896	Homeobox protein Hox-A9	59/5	2/6
C2_7364	Hyaluronan and proteoglycan link protein 1	45/4.3	5/8
C2_39681	Hyaluronan-binding protein 2	201/26.9	8/11
C2_465	Hydroxyacyl-coenzyme A dehydrogenase, mitochondrial	352/26.1	9/14
C2_610	Hydroxyacylglutathione hydrolase, mitochondrial	74/13.6	7/7
C2_1134	Hydroxymethylglutaryl-CoA lyase	43/8.4	6/6
C2_3330	Hydroxysteroid dehydrogenase-like protein 2	118/15.4	7/10
C2_679	Hypoxanthine-guanine phosphoribosyltransferase	114/16.8	13/17
C2_110618	Ig heavy chain Mem5	333/21.9	5/14
C2_114835	Ig heavy chain V-III region GAL	31/18.2	3/3
C2_58543	Ig kappa chain V region 3315	119/31.3	9/14
C2_121690	Ig kappa chain V-III region MOPC 63	122/25.8	6/7
C2_26869	Ig kappa-b4 chain C region	167/29.9	7/7
C2_107145	Ig lambda chain V-I region NEW	94/17.6	2/4
C2_108234	Ig lambda-6 chain C region	79/20.8	6/9
C2_46036	Ig mu chain C region	332/20.6	10/17
C2_717	Ig mu chain C region membrane-bound form	468/23.7	13/24
C2_65865	IgGFc-binding protein	58/7.5	2/2
C2_808	Immediate early response 3-interacting protein 1	50/5.5	5/12
C2_19893	Importin subunit alpha-4	522/21.6	9/27
C2_1404	Importin-7	37/3.4	3/6
C2_6463	Importin-9	40/5.3	4/5
C2_18835	Inactive rhomboid protein 1	38/7	4/6
C2_3141	Inhibitor of growth protein 4	37/4.1	3/17
C2_476	Inhibitor of growth protein 5	35/3.5	3/4
C2_23533	Inhibitor of nuclear factor kappa-B kinase subunit epsilon	41/6.5	6/8
C2_126	Inorganic pyrophosphatase	48/6	3/3

C2_109450	Inorganic pyrophosphatase 2, mitochondrial	100/29.4	5/6
C2_7657	Inosine triphosphate pyrophosphatase	51/5.7	1/1
C2_31224	Inosine-5'-monophosphate dehydrogenase 1	46/5.5	1/1
C2_1885	Inosine-5'-monophosphate dehydrogenase 2	81/11.3	7/10
C2_5546	Inositol 1,4,5-triphosphate receptor-interacting protein	273/10.2	17/33
C2_30594	Inositol 1,4,5-trisphosphate receptor type 1	51/3.7	3/3
C2_1723	Inositol hexakisphosphate kinase 2	48/4.2	4/5
C2_14114	Inositol monophosphatase 1	666/11.2	7/48
C2_78142	Inositol monophosphatase 2	62/20.2	3/3
C2_14682	Insulin-like growth factor 2 mRNA-binding protein 3	34/6.6	5/9
C2_4932	Integral membrane protein 2C	41/6.5	5/5
FP333792	Inter-alpha-trypsin inhibitor heavy chain H2	34/8.4	2/2
C2_12615	Inter-alpha-trypsin inhibitor heavy chain H3	103/3.4	4/7
C2_16818	Interferon-induced GTP-binding protein Mx	288/22.2	16/20
C2_82040	Interferon-induced helicase C domain-containing protein 1	38/7.1	2/2
C2_11371	Interferon-induced protein 44-like	43/3	2/2
C2_9595	Interferon-induced protein with tetratricopeptide repeats 1	42/4.4	4/6
C2_25725	Interleukin-18 receptor accessory protein	31/3.8	4/5
C2_757	Interleukin-6 receptor subunit alpha	55/6	6/9
C2_118270	Intermediate filament protein ON3	1431/59.8	10/126
C2_71380	Intestinal mucin-like protein	407/19.9	5/18
C2_44760	Intraflagellar transport protein 140 homolog	37/6.7	3/9
C2_116847	Iron-sulfur cluster assembly enzyme ISCU, mitochondrial	32/4.9	2/2
C2_17229	Isochorismatase domain-containing protein 2, mitochondrial	218/34.9	11/15
C2_3444	Isocitrate dehydrogenase [NAD] subunit gamma, mitochondrial	32/6.7	5/6
C2_1776	Isocitrate dehydrogenase [NADP] cytoplasmic	139/12.6	10/23
C2_8767	Isocitrate dehydrogenase [NADP], mitochondrial	176/12.6	8/13
C2_22347	Isocitrate dehydrogenase [NADP], mitochondrial	54/8.3	5/6
C2_46456	Isoleucyl-tRNA synthetase, cytoplasmic	182/21.1	8/12
C2_56026	Isoleucyl-tRNA synthetase, mitochondrial	76/12	1/1
C2_18025	IST1 homolog	130/21.8	5/7
C2_23019	Junction plakoglobin	2603/44	26/112
C2_1814	Junctional adhesion molecule A	134/11.7	10/13
C2_83200	Katanin p60 ATPase-containing subunit A-like 2	52/10.7	3/7
C2_11924	Kelch domain-containing protein 1	40/3.2	6/9
C2_13039	Kelch-like protein 12	42/2.8	2/3
C2_19579	Keratin, type I cytoskeletal 13	18155/72.3	43/708
C2_2372	Keratin, type I cytoskeletal 18	602/13.9	13/103
C2_86457	Keratin, type I cytoskeletal 19	1834/34.4	7/59
C2_51684	Keratin, type I cytoskeletal 50 kDa	314/28.7	6/19
AM971218	Keratin, type II cytoskeletal	50/5.5	1/1
C2_1442	Keratin, type II cytoskeletal 8	7349/37.1	37/443
C2_1049	KH domain-containing, RNA-binding, signal transduction-associated protein 1	59/2.8	5/9
C2_27379	Kinase suppressor of Ras 1	30/2.8	1/1
C2_3990	Kinectin	656/27.1	24/43
C2_7678	Kinesin light chain 1	31/6.1	4/4
C2_91639	Kinesin-1 heavy chain	39/12.2	3/3
C2_93570	Kinesin-like protein KIF1C	42/5.5	1/1
C2_10487	Kinetochore-associated protein NSL1 homolog	46/1.9	1/1
C2_29495	Kininogen (Fragments)	300/21.2	9/13
C2_23314	KN motif and ankyrin repeat domain-containing protein 1	33/2.3	2/2
C2_12624	Kunitz-type protease inhibitor 1	51/13.5	8/8
C2_3062	Kynurenine 3-monooxygenase	48/2.8	2/3
C2_17452	L antigen family member 3	48/5.7	2/2

C2_22722	L-2-hydroxyglutarate dehydrogenase, mitochondrial	57/6.4	3/3
C2_16409	Lactation elevated protein 1	38/5.4	3/10
C2_25570	Lactoperoxidase	193/11.8	4/10
C2_380	Lactoylglutathione lyase	169/16	10/11
C2_4354	Lambda-crystallin homolog	95/15.3	10/10
C2_43633	Lamin-B1	38/11.2	5/13
C2_19670	Lamin-B2	99/7.9	9/12
C2_14118	Lamin-L(II)	192/17.2	5/12
C2_4810	L-amino-acid oxidase	237/10.4	10/18
C2_68332	La-related protein 1	43/10.5	4/4
C2_4456	Large subunit GTPase 1 homolog	40/6.7	7/18
C2_141	Latexin	151/14.4	5/8
C2_121010	Lectoxin-Phi1	40/5.5	2/16
C2_186	Legumain	30/8	8/12
C2_68954	Leptin receptor gene-related protein	115/6.9	2/3
C2_93583	Leucine-rich repeat-containing G-protein coupled receptor 4	44/4.9	1/3
C2_12844	Leucine-rich repeat-containing protein 27	33/6.3	4/5
C2_29839	Leucine-rich repeat-containing protein C10orf11 homolog	35/9.1	5/12
C2_74310	Leucyl-tRNA synthetase, cytoplasmic	224/38.4	8/13
C2_76064	Leukocyte cysteine proteinase inhibitor 1	40/4.6	1/4
C2_2628	Leukocyte elastase inhibitor	85/9.1	11/28
C2_36942	Leukocyte receptor cluster member 9	38/4.4	2/3
C2_695	Leukotriene A-4 hydrolase	111/11.7	7/8
C2_80344	Lipopolysaccharide-responsive and beige-like anchor protein	168/11.9	2/3
C2_17725	Lissencephaly-1 homolog A	39/8.5	4/4
C2_1393	L-lactate dehydrogenase A chain	117/6.9	5/6
C2_6197	L-lactate dehydrogenase B chain	31/8.4	6/9
C2_117493	LON peptidase N-terminal domain and RING finger protein 2	74/14.9	1/1
C2_3	Low choriolytic enzyme	80/21.2	8/8
C2_57935	Low molecular weight phosphotyrosine protein phosphatase	95/22.9	8/11
C2_98485	Low-density lipoprotein receptor-related protein 2	43/2.4	1/3
C2_8471	Lumican	47/5.9	2/2
C2_17468	L-xylulose reductase	37/5.1	2/2
C2_27840	Lymphocyte antigen 6I	39/21.8	3/3
C2_2452	Lymphocyte antigen 75	217/13	13/22
C2_22063	Lymphocyte-specific protein 1	40/8.1	7/14
C2_115831	LYR motif-containing protein 4B	31/6.1	2/2
C2_52412	Lysine-specific demethylase 6A	38/4.4	3/5
C2_5997	LysM and putative peptidoglycan-binding domain-containing protein 3	40/2.8	3/8
C2_11596	Lysophospholipid acyltransferase 7	36/3.9	2/2
C2_3027	Lysosomal acid lipase/cholesteryl ester hydrolase	102/6.7	11/14
C2_2497	Lysosomal acid phosphatase	39/6.6	7/7
C2_8076	Lysosomal protective protein	71/8.2	5/11
C2_2284	Lysosome-associated membrane glycoprotein 2	48/6.5	4/7
C2_6251	Lysozyme g	152/7.1	4/5
C2_3105	Lysyl-tRNA synthetase	290/34.2	19/22
C2_20703	Macoilin-1	43/9.8	8/12
C2_7220	MACRO domain-containing protein 1	130/19.3	9/13
C2_4500	MACRO domain-containing protein 2	68/7.3	7/7
C2_3335	Macrophage colony-stimulating factor 1 receptor 2	52/3	4/5
C2_439	Macrophage migration inhibitory factor	157/13.6	5/6
C2_5305	Macrophage-capping protein	98/9.4	6/7
C2_4378	Macrosialin	37/5.1	3/3
C2_300	Magnesium transporter protein 1	77/8.9	10/19
C2_1864	Major facilitator superfamily domain-containing protein 10	77/6.3	3/3

C2_121640	Major histocompatibility complex class I-related gene protein	346/18	5/15
C2_72990	Major vault protein	154/31.9	4/11
C2_2796	Malate dehydrogenase	1776/29.7	19/76
C2_11452	Malate dehydrogenase, cytoplasmic	577/26.4	17/31
C2_612	Malate dehydrogenase, mitochondrial	2540/45.3	28/83
C2_98386	Malate synthase	374/12.1	4/14
C2_1108	Malignant T cell-amplified sequence 1-A	63/15.9	5/7
C2_9147	Mamu class II histocompatibility antigen, DR alpha chain	285/7.3	5/9
C2_23669	Mannose-1-phosphate guanyltransferase beta	48/8.5	3/5
C2_1921	Mannose-P-dolichol utilization defect 1 protein	131/12.5	8/11
C2_12214	MAP kinase-activated protein kinase 3	105/5.1	5/8
C2_25098	Max-binding protein MNT	30/8	2/11
C2_26268	Mediator of RNA polymerase II transcription subunit 22	37/4.4	2/15
C2_1730	Mediator of RNA polymerase II transcription subunit 6	60/7	4/8
C2_2487	Membrane-associated progesterone receptor component 1	30/5.8	5/6
C2_1690	Membrane-spanning 4-domains subfamily A member 12	37/10.3	6/7
C2_15744	Mesencephalic astrocyte-derived neurotrophic factor	200/26.7	6/7
C2_97466	Mesoderm induction early response protein 2	31/8.1	2/3
C2_1799	Metaxin-2	59/5.7	3/3
C2_40950	Methionine adenosyltransferase 2 subunit beta	40/12.5	3/3
C2_9097	Methionine aminopeptidase 2	110/7.1	3/3
C2_6737	Methionyl-tRNA synthetase, cytoplasmic	77/14	8/8
C2_3669	Methyl-CpG-binding domain protein 3	43/6.6	6/10
C2_62902	Methylcrotonoyl-CoA carboxylase subunit alpha, mitochondrial	38/10.3	2/3
C2_1710	Methylglutaconyl-CoA hydratase, mitochondrial	57/7.7	5/7
C2_5987	Methylmalonate-semialdehyde dehydrogenase [acylating], mitochondrial	210/23.4	12/15
C2_45829	Methylmalonyl-CoA epimerase, mitochondrial	37/5.7	2/2
C2_16016	Methylmalonyl-CoA mutase, mitochondrial	37/4	2/3
C2_5762	Methyltransferase-like protein 17, mitochondrial	38/7	4/8
C2_6033	Methyltransferase-like protein 23	32/5.7	2/4
C2_14451	MHC class II regulatory factor RFX1	31/3.7	3/3
C2_4020	Microfibrillar-associated protein 1	42/4.1	3/5
C2_26887	Microphthalmia-associated transcription factor	37/6.3	3/4
C2_830	Microsomal glutathione S-transferase 3	252/14.4	5/7
C2_15543	Microtubule-associated protein RP/EB family member 1	205/19.5	8/10
C2_51514	Midasin	46/9.2	2/5
C2_24629	Midline-1	35/2.4	2/6
C2_7863	Minor histocompatibility antigen H13	49/5	5/13
C2_8481	Mitochondrial 2-oxodicarboxylate carrier	49/4.5	2/4
C2_6253	Mitochondrial 2-oxoglutarate/malate carrier protein	106/13.3	6/7
C2_1996	Mitochondrial carrier homolog 2	184/9.6	10/15
C2_22005	Mitochondrial dynamic protein MID51	31/3.7	2/3
C2_198	Mitochondrial fission 1 protein	73/5.7	3/9
C2_44643	Mitochondrial fission process protein 1	46/7.6	1/1
C2_17904	Mitochondrial import receptor subunit TOM34	68/15.1	6/7
C2_3680	Mitochondrial inner membrane protein	265/15	9/11
C2_10488	Mitogen-activated protein kinase 1	85/7.9	3/7
C2_5937	Mixed lineage kinase domain-like protein	119/15.4	9/9
C2_2663	Mob-like protein phocein	36/4.5	3/3
C2_86	Moesin	355/13.6	20/42
C2_24890	Monocarboxylate transporter 7	39/5.1	6/13
C2_32779	Mothers against decapentaplegic homolog 2	49/5.7	2/2
C2_23192	Mothers against decapentaplegic homolog 3	30/3	3/5
C2_14284	M-phase phosphoprotein 6	69/8.7	3/9

C2_19684	Mps one binder kinase activator-like 1A	62/7	3/4
C2_480	Mps one binder kinase activator-like 1B	83/6	8/11
C2_3810	mTERF domain-containing protein 2	39/1.5	1/2
C2_2524	Mucin-5AC (Fragments)	37/5.5	4/12
C2_9660	Multidrug resistance protein 1	43/4	5/20
C2_3216	Multidrug resistance-associated protein 4	37/2	2/2
C2_3786	Multifunctional protein ADE2	397/32.1	16/52
C2_5717	Multiple inositol polyphosphate phosphatase 1	33/4.5	3/7
C2_4141	Multivesicular body subunit 12A	94/7.7	4/4
C2_10428	Myelin proteolipid protein	49/3.5	2/5
C2_8107	Myeloid cell surface antigen CD33	42/6.2	4/20
C2_78634	Myeloperoxidase	61/28.1	5/5
C2_27590	Myoferlin	109/10.5	2/2
C2_179	Myosin light chain 1, skeletal muscle isoform	117/12.9	6/15
C2_2556	Myosin light chain 3, skeletal muscle isoform	117/12.6	4/8
C2_3500	Myosin light polypeptide 6	286/23.5	9/14
C2_43	Myosin regulatory light chain 2, smooth muscle minor isoform	275/22.4	8/16
C2_2090	Myosin regulatory light polypeptide 9	111/7.6	6/9
C2_25606	Myosin-10	149/12.7	6/21
C2_29145	Myosin-11	66/2.9	1/1
C2_14197	Myosin-6	44/3.8	3/5
C2_515	Myosin-9	3710/42	63/174
C2_62971	Myosin-Ic	62/25.7	5/8
C2_28432	Myosin-Id	32/22.4	1/2
C2_59686	Myosin-Ie	33/5.8	1/1
C2_23428	Myosin-If	214/10.3	4/13
C2_52582	Myosin-Vc	44/9	5/8
C2_62194	Myosin-VI	53/6.7	1/1
C2_23167	Myosin-XV	208/4.8	4/14
C2_3970	Myotrophin	73/9	3/4
C2_4307	Myotubularin	62/3.3	3/3
C2_42501	Myotubularin-related protein 6	43/5.7	2/8
C2_21024	N(G),N(G)-dimethylarginine dimethylaminohydrolase 1	78/15.6	4/4
FM151450	N6-adenosine-methyltransferase 70 kDa subunit	42/3.8	1/5
C2_2369	N-acetyl-D-glucosamine kinase	87/11.5	4/4
C2_14558	N-acetylgalactosamine kinase	58/8.8	6/10
C2_11912	N-acetylneuraminate lyase	94/10.7	6/7
C2_109007	NACHT, LRR and PYD domains-containing protein 14	109/9.6	1/2
C2_115361	NACHT, LRR and PYD domains-containing protein 6	80/22.3	2/3
C2_98404	NACHT, LRR and PYD domains-containing protein 9B	45/8.8	2/4
C2_11708	NAD-dependent deacetylase sirtuin-1	39/5.2	5/10
C2_6042	NAD-dependent deacetylase sirtuin-5	39/7.1	4/5
C2_43421	NAD-dependent deacetylase sirtuin-6	30/7.4	3/3
C2_4705	NAD-dependent malic enzyme, mitochondrial	132/9.7	15/38
C2_8082	NADH dehydrogenase [ubiquinone] 1 alpha subcomplex subunit 1	46/12.8	3/5
C2_110117	NADH dehydrogenase [ubiquinone] 1 alpha subcomplex subunit 12	82/19.2	3/3
C2_1985	NADH dehydrogenase [ubiquinone] 1 alpha subcomplex subunit 2	64/13.5	2/2
C2_3631	NADH dehydrogenase [ubiquinone] 1 alpha subcomplex subunit 4	91/27.6	9/10
C2_9313	NADH dehydrogenase [ubiquinone] 1 alpha subcomplex subunit 6	34/11.9	2/2
C2_332	NADH dehydrogenase [ubiquinone] 1 alpha subcomplex subunit 9, mitochondrial	192/27.3	9/13
C2_497	NADH dehydrogenase [ubiquinone] 1 beta subcomplex subunit 10	115/17.2	5/5
C2_3428	NADH dehydrogenase [ubiquinone] 1 beta subcomplex subunit 4	47/19.6	3/4
C2_3928	NADH dehydrogenase [ubiquinone] 1 beta subcomplex subunit 6	90/29.1	5/5
C2_1170	NADH dehydrogenase [ubiquinone] 1 beta subcomplex subunit 7	41/6.4	1/1

C2_4941	NADH dehydrogenase [ubiquinone] 1 subunit C2	60/10.7	2/2
C2_3103	NADH dehydrogenase [ubiquinone] flavoprotein 2, mitochondrial	166/20.6	5/6
C2_1740	NADH dehydrogenase [ubiquinone] iron-sulfur protein 3, mitochondrial	119/21.2	8/8
C2_10276	NADH dehydrogenase [ubiquinone] iron-sulfur protein 6, mitochondrial	44/21	4/4
C2_1860	NADH dehydrogenase [ubiquinone] iron-sulfur protein 7, mitochondrial	52/8.5	3/3
C2_62722	NADH dehydrogenase [ubiquinone] iron-sulfur protein 8, mitochondrial	58/6.7	3/3
C2_568	NADH-cytochrome b5 reductase 3	83/21.8	8/10
C2_46308	NADH-cytochrome b5 reductase 3	44/10	4/6
C2_1488	NADH-ubiquinone oxidoreductase 75 kDa subunit, mitochondrial	42/11.3	9/11
C2_4956	NADP-dependent malic enzyme, mitochondrial	105/10	7/10
C2_1828	NADPH:adrenodoxin oxidoreductase, mitochondrial	37/4.9	4/11
C2_2964	NADPH--cytochrome P450 reductase (Fragments)	44/11.6	7/7
C2_30076	N-alpha-acetyltransferase 16, NatA auxiliary subunit	38/11.2	5/7
C2_15705	N-alpha-acetyltransferase 50	84/18.9	6/10
C2_31233	Nanos homolog 1	44/7	3/6
C2_34824	Nascent polypeptide-associated complex subunit alpha	513/14.8	4/11
C2_339	Nascent polypeptide-associated complex subunit alpha, muscle-specific form	513/17.2	7/14
C2_22742	Nattectin	457/23.2	7/20
C2_41935	Nck-associated protein 1	196/34.3	8/13
C2_3367	NEDD8	49/4.4	4/5
C2_6397	Negative elongation factor D	37/8.7	7/17
C2_2448	Neighbor of COX4	46/4.5	4/4
C2_6635	Neoverrucotoxin subunit alpha	350/10.1	19/37
C2_9617	Neoverrucotoxin subunit beta	145/14.2	9/32
C2_9448	Neprilysin	87/3.2	7/22
C2_68793	Neural cell adhesion molecule L1-like protein	47/20.1	3/4
C2_121614	Neurexin-1a-alpha	40/5.9	2/2
C2_25205	Neuroblast differentiation-associated protein AHNAK	555/47.4	32/57
C2_96825	Neuron navigator 2	39/7.2	2/14
C2_43849	Neuroserpin	31/10.7	1/1
C2_4237	Neutral alpha-glucosidase AB	115/10.4	9/9
C2_17550	Neutral amino acid transporter B(0)	126/8.3	5/9
C2_18906	Neutral ceramidase	40/5.3	2/6
C2_34370	NHL repeat-containing protein 2	30/8.1	3/3
C2_2429	NHP2-like protein 1	135/14.6	3/4
C2_5958	Nicastrin	61/7.7	8/9
C2_1457	Nicotinamide phosphoribosyltransferase	281/13.1	19/28
AM979735	Ninjurin-1	137/5	1/2
C2_13544	Nipped-B-like protein	50/3	2/2
C2_3276	N-lysine methyltransferase SMYD2-B	46/4.4	4/11
C2_4674	Nocturnin	41/4.8	5/11
C2_76382	Nodal modulator 1	31/7.2	1/1
C2_183	Non-specific cytotoxic cell receptor protein 1 homolog	104/17.6	5/9
C2_2827	Non-specific lipid-transfer protein	48/8.6	4/6
C2_4492	Non-syndromic hearing impairment protein 5	39/4.6	5/7
C2_2148	NTF2-related export protein 2	30/4.8	5/7
C2_14783	Nuclear factor 1 C-type	58/12	4/5
C2_20656	Nuclear factor 7, ovary	56/9.1	5/9
C2_82048	Nuclear factor NF-kappa-B p105 subunit	37/12.1	3/4
C2_8084	Nuclear pore complex protein Nup155	40/7.1	6/11
C2_12779	Nuclear pore complex protein Nup88	53/2.9	2/2
C2_16468	Nuclear pore complex protein Nup93	57/6.9	5/7
C2_10636	Nuclear protein localization protein 4 homolog	30/3.8	2/2
C2_7087	Nuclear receptor subfamily 5 group A member 2	44/5.5	5/5

C2_9241	Nuclear receptor-binding protein	73/4.1	2/2
C2_8072	Nuclear speckle splicing regulatory protein 1	34/5	5/9
C2_915	Nuclease-sensitive element-binding protein 1	58/6.3	4/4
C2_9442	Nucleolin	139/14.4	7/13
C2_27287	Nucleolysin TIA-1 isoform p40	54/5.5	3/4
C2_4203	Nucleoside diphosphate kinase 3	82/10.6	5/6
C2_7989	Nucleoside diphosphate kinase 6	34/4.7	4/4
C2_115	Nucleoside diphosphate kinase 7	1407/17	25/100
C2_113264	Nucleoside diphosphate kinase A1	407/25.9	7/18
C2_3380	Nucleosome assembly protein 1-like 1	61/7.4	4/6
C2_19448	Nucleosome assembly protein 1-like 4	124/10.8	4/6
C2_65706	Oligoribonuclease, mitochondrial	38/8.3	2/3
C2_2469	Ornithine decarboxylase antizyme 2	32/2.7	3/4
C2_846	Osteoclast-stimulating factor 1	118/10	5/5
FM150202	Oxysterol-binding protein-related protein 7	60/5.7	1/2
C2_29624	Oxysterol-binding protein-related protein 9	44/3.7	1/1
C2_2344	P2X purinoceptor 1	54/4.4	3/3
C2_9948	P2X purinoceptor 7	64/4.6	7/16
C2_11974	P2Y purinoceptor 3	42/4.5	6/9
C2_2885	p53 apoptosis effector related to PMP-22	152/14	8/16
C2_557	Palmitoyl-protein thioesterase 1	111/12.1	5/5
C2_19333	Papilin	43/6.7	4/4
C2_229	Parkinson protein 7	50/11.5	4/5
C2_20544	Parvalbumin, thymic CPV3	38/6	2/2
C2_6875	PCTP-like protein	176/18.2	8/13
C2_5258	PDZ and LIM domain protein 1	81/18.3	8/8
C2_52676	PDZ and LIM domain protein 2	123/22.9	4/5
C2_6912	PDZ and LIM domain protein 5	34/5.6	3/3
C2_8967	Peptide methionine sulfoxide reductase MsrA	71/8.1	4/10
C2_1296	Peptidyl-prolyl cis-trans isomerase	1731/41.5	16/63
C2_1675	Peptidyl-prolyl cis-trans isomerase B	349/18.2	6/20
C2_1790	Peptidyl-prolyl cis-trans isomerase F, mitochondrial	176/29.9	10/14
C2_7868	Peptidyl-prolyl cis-trans isomerase FKBP11	136/19.8	3/3
C2_24899	Peptidyl-prolyl cis-trans isomerase FKBP1A	146/16.7	4/11
C2_119898	Peptidyl-prolyl cis-trans isomerase FKBP3	49/14.8	6/6
C2_21705	Peptidyl-prolyl cis-trans isomerase FKBP9	39/3.3	2/3
C2_472	Peptidyl-prolyl cis-trans isomerase H	41/1.4	1/1
C2_8872	Peptidyl-prolyl cis-trans isomerase NIMA-interacting 1	94/5.6	3/3
C2_28915	Peptidyl-prolyl cis-trans isomerase-like 1	49/3.5	1/1
C2_6230	Peptidylprolyl isomerase domain and WD repeat-containing protein 1	32/3.6	2/2
C2_2443	Peptidyl-tRNA hydrolase 2, mitochondrial	64/7.7	4/9
C2_19295	Peptidyl-tRNA hydrolase ICT1, mitochondrial	55/5.1	2/2
C2_40424	Periplakin	1799/52.9	33/147
C2_1653	Peroxiredoxin 1	433/51.9	18/30
C2_2010	Peroxiredoxin 3	191/29.1	9/9
C2_579	Peroxiredoxin 4	351/50.8	15/17
C2_4821	Peroxiredoxin 5, mitochondrial	332/26.6	8/10
C2_199	Peroxiredoxin 6	324/28.9	9/18
C2_8400	Peroxisomal 2,4-dienoyl-CoA reductase	48/5.4	3/3
C2_11035	Peroxisomal membrane protein 4	30/9.4	3/9
C2_5419	Peroxisomal membrane protein PEX16	40/5	5/7
C2_4577	Peroxisomal multifunctional enzyme type 2	40/4.7	6/9
C2_11600	PERQ amino acid-rich with GYF domain-containing protein 2	39/4.1	5/7
C2_17726	PHD finger-like domain-containing protein 5A	83/21.9	4/4
C2_44498	Phenylalanine-4-hydroxylase	33/15.5	2/2

C2_2121	Phenylalanyl-tRNA synthetase alpha chain	111/10.8	5/5
C2_2810	Phenylalanyl-tRNA synthetase beta chain	105/19.7	12/13
C2_71999	Phosphatase and actin regulator 1	54/4.3	2/2
C2_457	Phosphate carrier protein, mitochondrial	244/22.2	16/26
C2_63463	Phosphate regulon transcriptional regulatory protein phoB	33/11.5	2/3
C2_3888	Phosphatidylcholine transfer protein	54/10.5	5/5
C2_99	Phosphatidylethanolamine-binding protein 1	1097/36.2	9/35
C2_4295	Phosphatidylinositide phosphatase SAC1-B	791/21.2	24/49
C2_1495	Phosphatidylinositol transfer protein alpha isoform	471/28	20/37
C2_18575	Phosphatidylinositol transfer protein beta isoform	187/38.4	9/9
C2_24090	Phosphatidylinositol-3,4,5-trisphosphate 5-phosphatase 2A	42/2.6	1/6
C2_24059	Phosphatidylinositol-3,4,5-trisphosphate 5-phosphatase 2B	53/9.3	2/10
C2_19167	Phosphoacetylglucosamine mutase	139/15.7	5/5
C2_12073	Phosphoenolpyruvate carboxykinase [GTP], mitochondrial	136/11.5	9/9
C2_6725	Phosphoglucomutase-2	80/10.6	9/9
C2_1379	Phosphoglycerate kinase 1	640/14.8	16/42
C2_2366	Phosphoglycerate mutase 1	404/27.8	12/22
C2_85	Phospholipid hydroperoxide glutathione peroxidase, mitochondrial	46/6.6	1/1
C2_2880	Phospholysine phosphohistidine inorganic pyrophosphate phosphatase	34/6.4	4/4
C2_1982	Phosphomannomutase 2	90/8.6	8/13
C2_4391	Phosphorylase b kinase regulatory subunit beta	36/2.8	3/5
C2_1208	Phosphoserine aminotransferase	62/6.5	3/5
C2_19159	Phosphotriesterase-related protein	36/3.2	3/3
C2_31716	Phytanoyl-CoA dioxygenase domain-containing protein 1	48/5.2	2/2
C2_12242	Piezo-type mechanosensitive ion channel component 2	44/2.3	2/2
C2_7139	PITH domain-containing protein 1	78/7.2	4/6
AM957606	Pituitary homeobox 2	35/4.2	1/1
C2_10398	Pituitary tumor-transforming gene 1 protein-interacting protein	32/4.4	2/3
C2_1194	Placenta-specific gene 8 protein	52/5.3	2/8
C2_27201	Plakophilin-1	167/8.9	3/5
C2_13726	Plakophilin-3	392/24.6	15/36
C2_4530	Plasma alpha-L-fucosidase	35/4.1	3/6
C2_24573	Plasma membrane calcium-transporting ATPase 2	58/10.4	9/12
C2_184	Plasminogen activator inhibitor 1 RNA-binding protein	55/6.1	3/4
C2_4248	Plastin-1	42/5	4/5
C2_952	Plastin-2	937/39.8	24/45
C2_6788	Plastin-3	924/28.7	24/49
C2_2316	Platelet-activating factor acetylhydrolase IB subunit gamma	226/10.6	7/11
C2_10096	Platelet-derived growth factor receptor-like protein	30/8	4/4
C2_1339	Pleckstrin	32/4	4/9
C2_6665	Pleckstrin homology domain-containing family M member 2	39/2.4	3/4
C2_16967	Plectin	1648/23.9	17/58
C2_63006	Plectin	203/45.4	11/20
C2_98090	Plexin-B2	37/4.7	2/2
C2_49013	Poly [ADP-ribose] polymerase 12	30/6.5	2/2
C2_4201	Poly [ADP-ribose] polymerase 3	319/19.5	12/14
C2_17116	Poly(A) polymerase gamma	39/4.4	4/20
C2_4828	Poly(rC)-binding protein 2	43/3.2	3/3
C2_54396	Poly(U)-binding-splicing factor PUF60-B	39/10	1/1
C2_3168	Polyadenylate-binding protein 1	391/13.8	12/22
C2_12680	Polyadenylate-binding protein 2	81/15.3	7/8
C2_9708	Polypeptide N-acetylgalactosaminyltransferase 6	84/7.7	5/10
C2_8361	Polypyrimidine tract-binding protein 1	129/12.8	10/14
C2_15477	Polyribonucleotide nucleotidyltransferase 1, mitochondrial	34/3.3	4/5
C2_4566	Porphobilinogen deaminase	42/2.1	3/3

C2_47948	POU domain, class 4, transcription factor 3	44/6.9	2/3
C2_653	Prefoldin subunit 2	136/9.1	4/5
C2_1949	Prefoldin subunit 3	46/16.5	5/8
C2_41817	Pregnancy zone protein	85/24.4	6/7
C2_17155	Prelamin-A/C	46/9.1	6/10
C2_1514	Pre-mRNA-processing factor 19	220/26.6	14/17
C2_3804	Pre-mRNA-processing factor 39	114/12.6	13/17
C2_10800	Pre-mRNA-processing-splicing factor 8	186/11.9	7/12
C2_28626	Prickle-like protein 2	41/7.9	3/14
C2_1682	Probable aminopeptidase NPEPL1	349/15.7	8/14
C2_7253	Probable ATP-dependent RNA helicase DDX10	35/5.7	5/7
C2_19562	Probable ATP-dependent RNA helicase DDX5	63/6.6	3/3
C2_2745	Probable ATP-dependent RNA helicase ddx6	123/8.8	12/19
C2_20500	Probable Bax inhibitor 1	34/4.2	1/1
C2_51219	Probable E3 ubiquitin-protein ligase TRIM8	31/6.6	2/7
C2_69516	Probable G-protein coupled receptor 132	42/7.5	3/3
C2_96751	Probable histone deacetylase 1-A	112/18.3	3/4
C2_1359	Probable methylthioribulose-1-phosphate dehydratase	38/4.8	3/3
C2_7381	Probable palmitoyltransferase ZDHHC20	93/1.7	3/7
C2_5342	Probable palmitoyltransferase ZDHHC6	41/2.4	2/5
C2_1019	Probable saccharopine dehydrogenase	417/8.8	8/19
C2_242	Probable signal peptidase complex subunit 2	84/9.9	4/7
C2_8372	Probable threonyl-tRNA synthetase 2, cytoplasmic	92/7.2	7/17
C2_1594	Probable tRNA threonylcarbamoyladenosine biosynthesis protein osgep	156/12	8/10
C2_27370	Probable tRNA(His) guanylyltransferase	38/7.9	2/2
C2_19855	Probable ubiquitin carboxyl-terminal hydrolase FAF-X	38/6	4/4
C2_12479	Probable UDP-sugar transporter protein SLC35A4	37/5.3	4/11
C2_212	Pro-cathepsin H	41/5.5	3/3
C2_22976	Profilin-1	249/25.2	7/11
C2_2738	Profilin-2	96/10.1	7/14
C2_4359	Programmed cell death 1 ligand 1	37/4.9	4/12
C2_22586	Programmed cell death 6-interacting protein	347/31.4	9/18
C2_21771	Programmed cell death protein 10	42/10.3	5/7
C2_20093	Programmed cell death protein 4	117/15.8	6/10
C2_576	Programmed cell death protein 6	136/16.2	8/9
C2_4581	Programmed cell death protein 7	64/4.9	5/6
C2_285	Prohibitin	157/11.9	7/17
C2_1142	Prohibitin-2	212/19.4	9/18
C2_228	Proliferating cell nuclear antigen	45/5.2	3/3
C2_447	Proliferation-associated protein 2G4	141/12.3	11/15
C2_816	Proline synthase co-transcribed bacterial homolog protein	83/15	5/5
C2_7079	Proline-rich protein PRCC	32/5.3	7/7
C2_35352	Prolyl endopeptidase	267/38.4	9/13
C2_8219	Propionyl-CoA carboxylase alpha chain, mitochondrial	66/9	8/13
C2_6056	Propionyl-CoA carboxylase beta chain, mitochondrial	109/11.3	8/8
C2_18955	Prostaglandin E synthase 2	31/1.6	1/7
C2_4229	Prostamide/prostaglandin F synthase	65/11.9	3/3
C2_1731	Protease-associated domain-containing protein 1	40/2.5	2/9
C2_62037	Proteasomal ubiquitin receptor ADRM1	34/25.3	3/10
C2_7901	Proteasomal ubiquitin receptor ADRM1-A	38/5	2/3
C2_19056	Proteasome activator complex subunit 1	643/48.5	15/61
C2_52053	Proteasome activator complex subunit 2	621/36.3	15/37
C2_17033	Proteasome activator complex subunit 4	41/1.6	1/3
C2_8230	Proteasome assembly chaperone 2	42/6.1	5/7
C2_1050	Proteasome assembly chaperone 3	32/10.8	3/9

C2_8536	Proteasome inhibitor PI31 subunit	34/3.7	2/3
C2_276	Proteasome subunit alpha type-1	570/42.7	18/42
C2_39253	Proteasome subunit alpha type-2	777/29.5	10/19
C2_667	Proteasome subunit alpha type-3	494/35	14/30
C2_1506	Proteasome subunit alpha type-4	590/33	8/27
C2_89	Proteasome subunit alpha type-5	543/31.3	11/24
C2_979	Proteasome subunit alpha type-6	502/40.1	15/27
C2_486	Proteasome subunit alpha type-7	674/38.8	14/31
C2_53426	Proteasome subunit beta type-10	55/17.3	4/6
C2_303	Proteasome subunit beta type-1-B	372/15.9	8/15
C2_4220	Proteasome subunit beta type-2	303/26.4	8/17
C2_1113	Proteasome subunit beta type-3	467/37.5	10/17
C2_1989	Proteasome subunit beta type-4	384/41.3	10/17
C2_2719	Proteasome subunit beta type-5	69/9.5	4/4
C2_43253	Proteasome subunit beta type-6	154/28.6	10/10
C2_104936	Proteasome subunit beta type-6-B like protein	194/26.5	6/11
C2_1603	Proteasome subunit beta type-8	287/19.7	12/26
C2_4274	Proteasome subunit beta type-9	76/14.3	7/9
C2_2100	Protein AMBP	177/13.2	6/8
C2_2985	Protein arginine N-methyltransferase 1	153/14.4	8/12
C2_5961	Protein argonaute-2	181/6.7	10/14
C2_120051	Protein BMH1	177/29.4	3/20
C2_2048	Protein BUD31 homolog	61/2.9	1/1
C2_5817	Protein chibby homolog 1	377/10.6	6/47
C2_1344	Protein Churchill	44/13.1	4/5
C2_100838	Protein CutA homolog	59/14	3/3
C2_3894	Protein disulfide-isomerase	861/29.1	34/55
C2_251	Protein disulfide-isomerase A3	983/34.2	20/49
C2_2492	Protein disulfide-isomerase A4	249/19.1	13/16
C2_37040	Protein disulfide-isomerase A5	58/14.1	2/2
C2_10370	Protein disulfide-isomerase A6	181/17.5	11/20
C2_2641	Protein DPCD	31/4.8	3/3
C2_9522	Protein dpy-19 homolog 4	42/7.6	5/5
C2_1216	Protein ETHE1, mitochondrial	134/9.8	8/11
C2_30518	Protein FAM110C	73/10	4/9
C2_11485	Protein FAM114A2	39/2.6	3/3
C2_6080	Protein FAM115	630/17.1	12/25
C2_20394	Protein FAM118B	45/3.5	4/10
C2_6170	Protein FAM160B1	52/3.9	6/12
C2_12775	Protein FAM26E	44/8.1	5/5
C2_6517	Protein FAM36A	43/10	4/10
C2_1887	Protein FAM49B	39/6.8	5/6
C2_10389	Protein FAM53C	35/7.6	7/19
C2_43947	Protein FAM55C	36/5.8	2/2
C2_15938	Protein FAM83H	60/3.6	4/11
C2_28268	Protein FAM91A1	82/11.5	4/15
C2_31326	Protein fat-free	44/5.2	1/7
C2_15037	Protein furry homolog-like	157/2.5	1/15
C2_7128	Protein GPR108	34/4.8	2/2
C2_10282	Protein IWS1 homolog	39/10.1	3/5
C2_5831	Protein jagunal homolog 1-B	93/4.9	2/2
C2_66932	Protein KIAA0664 homolog	62/21.7	5/7
C2_30726	Protein kinase C alpha type	102/10.3	8/15
C2_1038	Protein kinase C and casein kinase substrate in neurons protein 2	40/3.9	5/6
C2_9126	Protein kinase C beta type	107/13.1	8/23

C2_6026	Protein LAS1 homolog	37/5	5/7
C2_158	Protein LSM12 homolog A	69/7.4	3/3
C2_24702	Protein mago nashi homolog 2	154/17.7	7/9
C2_1083	Protein NDRG1	97/6.3	8/9
C2_1013	Protein NipSnap homolog 2	59/3.8	4/7
C2_2272	Protein NipSnap homolog 3A	102/20.2	8/12
C2_56802	Protein O-linked-mannose beta-1,2-N-acetylglucosaminyltransferase 1	207/20.5	7/8
C2_4186	Protein phosphatase Slingshot homolog	38/4.9	3/3
C2_14239	Protein polybromo-1	42/2.9	2/4
C2_2446	Protein Red	41/5.9	6/10
C2_2956	Protein RER1	89/16.8	6/6
C2_1280	Protein S100-A10	228/18	5/10
C2_19606	Protein S100-A14	100/7	2/4
C2_24087	Protein S100-A16	38/3.7	1/1
C2_4144	Protein SEC13 homolog	129/20.3	7/8
C2_8005	Protein SET	95/4.2	4/8
C2_37688	Protein SPT2 homolog	41/11.7	6/8
C2_12184	Protein strawberry notch homolog 2	35/4.1	6/9
C2_89684	Protein TFG	66/16.1	2/2
C2_15806	Protein transport protein Sec23A	93/9.2	4/5
C2_7030	Protein transport protein Sec24A	38/4.2	4/13
C2_40599	Protein transport protein Sec24C	60/10.7	2/12
C2_45211	Protein transport protein Sec31A	36/4.3	1/2
C2_324	Protein transport protein Sec61 subunit alpha-like 1	341/10.4	8/14
C2_1291	Protein transport protein Sec61 subunit beta	75/18.6	6/11
C2_70877	Protein turtle homolog B	46/2.3	1/3
C2_1220	Protein tyrosine phosphatase type IVA 2	78/4.3	3/3
C2_20295	Protein tyrosine phosphatase type IVA 3	56/3.4	3/8
C2_13679	Protein YIPF6	31/6.7	6/6
C2_13589	Protein-arginine deiminase type-2	1080/22.6	24/60
C2_107666	Proteolipid protein 2	64/13.7	4/7
C2_31468	Prothrombin	88/23.2	3/4
C2_69301	Protocadherin gamma-A12	38/11.2	2/2
C2_13732	Protocadherin-like wing polarity protein stan	44/4.5	4/9
C2_16519	Pterin-4-alpha-carbinolamine dehydratase	97/10	5/5
C2_21104	Puromycin-sensitive aminopeptidase	167/17.7	9/9
C2_102229	Putative 60S ribosomal protein L37a	233/38	7/16
C2_1163	Putative adenosylhomocysteinase 3	135/4.8	3/11
C2_962	Putative aminopeptidase W07G4.4	1372/37.6	23/53
C2_31441	Putative ATP-dependent RNA helicase an3	72/13.2	7/7
C2_18015	Putative deoxyribose-phosphate aldolase	103/9.7	5/6
C2_4760	Putative L-aspartate dehydrogenase	44/7	3/6
C2_22381	Putative nascent polypeptide-associated complex subunit alpha-like protein	646/22.2	4/16
C2_4814	Putative P2Y purinoceptor 10	50/4.4	5/8
C2_105694	Putative protein PHLOEM PROTEIN 2-LIKE A3	42/15.3	2/2
C2_6662	Putative sodium-coupled neutral amino acid transporter 7	39/2.9	3/7
C2_8233	Pyridoxal kinase	34/4	4/11
C2_107720	Pyrin	98/12.3	4/8
C2_20729	Pyrroline-5-carboxylate reductase 1, mitochondrial	50/8.3	2/2
C2_65516	Pyrroline-5-carboxylate reductase 2	51/7.2	1/1
C2_2292	Pyruvate dehydrogenase E1 component subunit alpha, somatic form, mitochondrial	91/6.4	8/13
C2_2277	Pyruvate dehydrogenase E1 component subunit beta, mitochondrial	362/22	12/15
C2_13692	Pyruvate kinase isozyme M1	611/15.5	9/25

C2_96589	Pyruvate kinase isozymes M1/M2	365/35.1	8/25
C2_7049	Pyruvate kinase isozymes R/L	99/21.2	2/5
C2_2874	Pyruvate kinase muscle isozyme	552/13.3	9/44
C2_1390	Quinone oxidoreductase	55/7.2	4/6
C2_5856	R3H domain-containing protein C19orf22 homolog	38/4.5	6/9
C2_32560	Rab GDP dissociation inhibitor alpha	41/23.3	4/6
C2_455	Rab GDP dissociation inhibitor beta	1397/38.1	26/63
C2_11802	Rab GTPase-binding effector protein 2	46/7.6	9/12
C2_3297	Rab proteins geranylgeranyltransferase component A 1	95/6.2	6/14
C2_35707	Rab11 family-interacting protein 2	38/5.4	1/3
C2_16564	Radixin	164/17.3	13/29
C2_106557	Ragulator complex protein LAMTOR3	81/18.6	2/2
C2_107555	Rano class II histocompatibility antigen, D-1 beta chain	226/24.4	5/9
C2_24742	Ran-specific GTPase-activating protein	246/31	8/12
C2_97593	Rap guanine nucleotide exchange factor 4	39/21.5	5/10
C2_10857	Rap1 GTPase-GDP dissociation stimulator 1	35/7.6	10/15
C2_4283	Ras association domain-containing protein 1	36/11.4	7/13
C2_1935	Ras GTPase-activating protein 2	37/9.2	3/5
C2_5031	Ras GTPase-activating-like protein IQGAP1	174/15.3	16/28
C2_7014	Ras GTPase-activating-like protein IQGAP2	60/4.4	5/7
C2_1097	Ras-related C3 botulinum toxin substrate 1	343/15.4	15/28
C2_121610	Ras-related C3 botulinum toxin substrate 2	316/32.3	8/22
C2_6071	Ras-related protein Rab-10	966/18.6	19/59
C2_3156	Ras-related protein Rab-11A	344/16.2	15/34
C2_452	Ras-related protein Rab-11B	379/17.7	18/42
C2_10036	Ras-related protein Rab-14	283/19.6	14/22
C2_7624	Ras-related protein Rab-1A	1046/31.8	18/55
C2_67687	Ras-related protein Rab-1B	149/13.6	1/2
C2_6604	Ras-related protein Rab-21	53/5.4	2/3
C2_508	Ras-related protein Rab-25	169/15	9/15
C2_4796	Ras-related protein Rab-27A	62/6.6	7/8
C2_1465	Ras-related protein Rab-2A	310/23.3	13/17
C2_11923	Ras-related protein Rab-30	89/5	3/7
C2_14787	Ras-related protein Rab-33B	123/13.4	9/17
C2_2646	Ras-related protein Rab-34	60/5.2	4/5
C2_573	Ras-related protein Rab-35	315/17.4	7/22
C2_15936	Ras-related protein Rab-3C	119/4.7	6/10
C2_9276	Ras-related protein Rab-4B	89/7.4	9/14
C2_4291	Ras-related protein Rab-5A	235/21.4	10/14
C2_6579	Ras-related protein Rab-5B	158/3.9	4/8
C2_7828	Ras-related protein Rab-5C	332/19.7	11/19
C2_30388	Ras-related protein Rab-6A	146/21.6	9/13
C2_53708	Ras-related protein Rab7	181/12.4	5/8
C2_523	Ras-related protein Rab-7a	612/18.9	14/28
C2_5197	Ras-related protein Rab-8A	428/10.4	11/30
C2_935	Ras-related protein Ral-B	89/8.5	9/17
C2_879	Ras-related protein Rap-1b	407/23.1	10/17
C2_18047	Ras-related protein Rap-1b-like protein	245/15.7	7/11
C2_23094	Ras-related protein R-Ras	51/3.4	2/5
C2_7026	Ras-related protein R-Ras2	69/8.5	5/6
C2_1650	Receptor expression-enhancing protein 5	76/12.3	4/6
C2_2387	Receptor-type tyrosine-protein phosphatase C	67/3.5	4/5
C2_8301	Receptor-type tyrosine-protein phosphatase eta	30/4.3	2/5
C2_1647	Regucalcin	133/22.8	8/8
C2_8435	Regulator of chromosome condensation	96/11.4	6/7

C2_19184	Regulator of G-protein signaling 1	44/3.1	2/2
C2_81776	Regulator of G-protein signaling 21	69/8.7	2/2
C2_17814	Regulator of G-protein signaling 4	38/7.7	3/4
C2_97685	Regulator of nonsense transcripts 1	39/7.9	2/2
C2_606	Reticulon-3	678/14.4	13/35
C2_1570	Reticulon-4	48/18.8	5/7
C2_25541	Retinal dehydrogenase 1	38/9.7	1/1
C2_2184	Retinoic acid receptor RXR-beta-A	33/5.5	7/8
C2_1894	Retinoic acid receptor RXR-gamma-B	33/5.7	4/10
C2_223	Retinoid-inducible serine carboxypeptidase	53/8.1	7/8
C2_3697	Retinol dehydrogenase 12	85/18.1	8/10
C2_6098	Retinol dehydrogenase 13	47/7.3	5/14
C2_41464	Retinol dehydrogenase 7	161/10.8	7/9
C2_1604	Rho GDP-dissociation inhibitor 1	813/18.6	12/32
C2_37660	Rho GTPase-activating protein 10	38/7.3	3/6
C2_4323	Rho GTPase-activating protein 25	36/2.5	3/3
C2_22184	Rho guanine nucleotide exchange factor 5	35/8.6	5/10
C2_30428	Rho-related GTP-binding protein RhoC	668/32.6	13/31
C2_11478	Rho-related GTP-binding protein RhoG	224/36.4	11/16
C2_20423	Ribonuclease inhibitor	93/14.8	6/10
C2_13816	Ribonuclease UK114	269/16	7/8
C2_1415	Ribose-phosphate pyrophosphokinase 1	218/14.5	9/9
C2_3757	Ribose-phosphate pyrophosphokinase 2	215/15	10/12
C2_2840	Ribosomal protein S6 kinase 2 alpha	93/14.2	11/12
C2_38170	Ribosomal protein S6 kinase alpha-3	123/7.1	3/4
C2_1091	Ribosome maturation protein SBDS	70/15.1	6/12
C2_959	Ribosome production factor 2 homolog	34/9	6/11
C2_752	Ribosome-binding protein 1	1447/12.1	20/71
C2_79457	Ribulose-phosphate 3-epimerase	42/12.1	1/1
C2_5832	RING finger protein 185	36/2.2	3/6
C2_121535	RLA class II histocompatibility antigen, DP alpha-1 chain	357/30.3	5/9
C2_110373	RLA class II histocompatibility antigen, DP beta chain	384/21.4	4/9
C2_4962	RNA exonuclease 4	40/7.6	6/7
C2_6712	RNA methyltransferase-like protein 1A	40/5.5	3/5
C2_5423	RNA-binding protein 4	131/8.3	3/4
C2_13076	RNA-binding protein 45	49/3.2	2/3
C2_21199	RNA-binding protein 8A	40/8.6	1/2
C2_9599	rRNA 2'-O-methyltransferase fibrillarin	56/12.3	4/5
C2_9420	RUN and FYVE domain-containing protein 1	37/2.4	2/3
C2_3458	RuvB-like 1	85/10.9	5/5
C2_57479	Ryanodine receptor 3	45/7.1	2/2
C2_32775	SAM domain and HD domain-containing protein 1	440/7.7	2/19
C2_904	Sarcoplasmic/endoplasmic reticulum calcium ATPase 1	187/8.7	10/12
C2_58759	Sarcoplasmic/endoplasmic reticulum calcium ATPase 2	309/33.6	4/9
C2_1769	Sarcoplasmic/endoplasmic reticulum calcium ATPase 3	418/25.9	15/22
C2_1565	Scavenger mRNA-decapping enzyme DcpS	47/15.3	8/9
C2_13913	Sciellin	926/45.6	22/59
C2_3154	Secretory carrier-associated membrane protein 2	68/8.2	6/9
C2_1954	Sel1 repeat-containing protein 1	54/1.3	1/2
C2_28759	Semaphorin-3C	42/8.2	3/4
C2_48720	Sentrin-specific protease 7	32/15.1	2/4
C2_16505	Septin-10	44/3.5	2/3
C2_5603	Septin-2	155/9.2	7/11
C2_22270	Septin-6	80/7.6	2/5
C2_8875	Septin-7	170/26.2	9/14

C2_9888	Septin-8-A	155/13	7/11
C2_13008	Serine hydroxymethyltransferase, mitochondrial	31/6.8	4/7
C2_2494	Serine palmitoyltransferase 1	74/7.6	8/9
C2_1270	Serine/arginine-rich splicing factor 2	69/4.7	5/7
C2_18056	Serine/arginine-rich splicing factor 9	37/13	4/7
C2_6871	Serine/threonine-protein kinase 10	732/9.2	13/38
C2_6812	Serine/threonine-protein kinase 16	40/4.4	6/9
C2_91193	Serine/threonine-protein kinase 38	33/6	1/1
C2_24435	Serine/threonine-protein kinase D2	49/7.6	6/7
C2_71318	Serine/threonine-protein kinase Nek1	41/7.3	2/2
C2_5769	Serine/threonine-protein kinase Nek7	30/2.3	1/1
C2_3226	Serine/threonine-protein kinase PAK 2	38/4.6	5/10
C2_6002	Serine/threonine-protein kinase RIO1	33/5.1	4/5
C2_26513	Serine/threonine-protein kinase SIK2	34/5.3	2/3
C2_35102	Serine/threonine-protein kinase SRPK2	36/10	3/6
C2_10352	Serine/threonine-protein kinase SRPK3	36/9.4	9/13
C2_4691	Serine/threonine-protein kinase VRK1	82/8.5	4/6
C2_8940	Serine/threonine-protein phosphatase 1 regulatory subunit 10	38/1.4	1/4
C2_5266	Serine/threonine-protein phosphatase 2A 56 kDa regulatory subunit gamma isoform	38/2.3	4/5
C2_7698	Serine/threonine-protein phosphatase 2A 65 kDa regulatory subunit A beta isoform	795/16.2	12/33
C2_11251	Serine/threonine-protein phosphatase 2A catalytic subunit alpha isoform	361/15.9	12/22
C2_7368	Serine/threonine-protein phosphatase 2A catalytic subunit beta isoform	447/27.3	12/24
C2_22338	Serine/threonine-protein phosphatase 2B catalytic subunit alpha isoform	209/26.3	10/15
C2_90751	Serine/threonine-protein phosphatase 4 catalytic subunit	42/5.7	1/1
C2_23324	Serine/threonine-protein phosphatase 4 catalytic subunit A	40/10.8	2/5
C2_2026	Serine/threonine-protein phosphatase 4 catalytic subunit B	100/7	5/8
C2_7354	Serine/threonine-protein phosphatase 5	53/5.3	5/6
C2_2436	Serine/threonine-protein phosphatase 6 catalytic subunit	44/9.4	8/8
C2_84628	Serine/threonine-protein phosphatase 6 regulatory ankyrin repeat subunit C	48/10.9	1/1
C2_12297	Serine/threonine-protein phosphatase PGAM5, mitochondrial	92/11.6	5/5
C2_25240	Serine/threonine-protein phosphatase PP1-alpha catalytic subunit	161/15.3	6/9
C2_1167	Serine/threonine-protein phosphatase PP1-beta catalytic subunit	377/36.4	12/19
C2_553	Serine/threonine-protein phosphatase PP1-gamma catalytic subunit	350/20.7	11/18
C2_79340	Serine/threonine-protein phosphatase PP2A-1 catalytic subunit	33/13.6	2/2
C2_866	Serine-threonine kinase receptor-associated protein	148/10.6	5/6
C2_110965	Serpin B8	54/15.7	2/4
C2_14294	Serpin peptidase inhibitor, clade A (alpha-1 antiproteinase, antitrypsin), member 1	136/21.1	8/13
C2_9982	Serpin peptidase inhibitor, clade F (alpha-2 antiplasmin, pigment epithelium derived factor), member 2	35/9.1	7/14
C2_14608	Serum amyloid P-component	39/5.8	3/5
C2_66732	Serum deprivation-response protein	89/6.6	2/3
C2_7237	Seryl-tRNA synthetase, cytoplasmic	580/34.6	20/29
C2_7917	Sestrin-2	40/5.3	3/4
C2_778	S-formylglutathione hydrolase	201/25.7	8/9
C2_107681	SH3 domain-binding glutamic acid-rich-like protein 3	30/3.7	1/1
C2_4801	SHC-transforming protein 1	42/6	9/15
C2_61267	Short transient receptor potential channel 4	39/15.8	2/3
C2_2197	Sialic acid synthase	105/10.1	6/8
C2_3266	Sialic acid-binding Ig-like lectin 15	37/7.8	6/12
C2_14736	Sialic acid-binding Ig-like lectin 7	39/11.8	5/11
C2_8533	Sialoadhesin	46/3.7	3/4

C2_54523	Sideroflexin-3	53/11.1	2/2
C2_15619	Sideroflexin-4	39/5.2	4/9
C2_72	Signal peptidase complex catalytic subunit SEC11A	76/10.4	5/6
C2_3318	Signal peptidase complex subunit 3	45/19.9	6/8
C2_672	Signal recognition particle 14 kDa protein	66/3.8	4/4
C2_45522	Signal recognition particle 68 kDa protein	30/26	3/3
C2_7347	Signal recognition particle 72 kDa protein	46/12.8	4/4
C2_1865	Signal recognition particle 9 kDa protein	57/5.6	2/2
C2_95561	Signal recognition particle receptor subunit alpha	39/3.3	1/2
C2_627	Signal recognition particle receptor subunit beta	63/7.5	6/9
C2_2871	Signal transducer and activator of transcription 3	31/10.8	13/19
C2_20239	Signal-regulatory protein beta-2	45/9.4	4/6
C2_2831	Single-stranded DNA-binding protein, mitochondrial	73/6	3/5
C2_20869	Sister chromatid cohesion protein PDS5 homolog A	91/9.6	2/2
C2_44615	Sister chromatid cohesion protein PDS5 homolog B	93/5.2	2/5
C2_4819	Small nuclear ribonucleoprotein E	124/20.2	4/6
C2_27604	Small nuclear ribonucleoprotein F	98/10.8	2/3
C2_44786	Small nuclear ribonucleoprotein G	36/17.5	4/4
C2_68502	Small nuclear ribonucleoprotein Sm D1	100/17.2	3/4
C2_2690	Small nuclear ribonucleoprotein Sm D2	131/22.3	5/5
C2_7038	Small nuclear ribonucleoprotein Sm D3	229/26.5	5/10
C2_63585	Small ubiquitin-related modifier 3	109/21.8	4/5
C2_116817	SMH class II histocompatibility antigen, beta-1 chain	339/18.9	3/8
C2_21636	Sodium- and chloride-dependent GABA transporter 3	32/2.2	1/1
C2_82623	Sodium/calcium exchanger 3	41/5.9	2/4
C2_28204	Sodium/hydrogen exchanger 8	61/1.6	1/5
C2_837	Sodium/potassium-transporting ATPase subunit alpha-1	2815/44.4	40/110
C2_5237	Sodium/potassium-transporting ATPase subunit alpha-3	2694/40.6	50/127
C2_8762	Sodium-coupled neutral amino acid transporter 2	40/4.7	3/6
C2_9006	Solute carrier family 12 member 2	99/6.7	4/4
C2_9328	Solute carrier family 12 member 9	40/2.1	1/4
C2_1662	Solute carrier family 13 member 2	41/4.2	6/10
C2_20129	Solute carrier family 2, facilitated glucose transporter member 1	49/2.6	2/3
C2_40766	Solute carrier family 25 member 34	39/6.4	3/5
C2_27770	Solute carrier family 40 member 1	37/4.2	4/11
C2_1290	Sorbitol dehydrogenase	316/30.7	16/33
C2_408	Sorcin	69/4.8	3/3
C2_2983	Sorting nexin-1	63/12.7	12/14
C2_59693	Sorting nexin-11	36/13.5	2/3
C2_629	Sorting nexin-12	34/7.9	3/8
C2_963	Spectrin alpha chain, brain	106/6.1	6/7
C2_46596	Spectrin beta chain, brain 1	54/7.6	3/6
C2_56894	Spectrin beta chain, brain 2	55/11.2	2/3
C2_37239	Sperm acrosome membrane-associated protein 4	202/21.8	4/7
C2_19938	Spermatogenesis-associated protein 13	40/5.7	3/3
C2_8146	Spermidine synthase	60/14.5	4/4
C2_3740	Spermine synthase	44/6.4	7/10
C2_159	S-phase kinase-associated protein 1	38/14.8	6/7
C2_1133	Sphingolipid delta(4)-desaturase DES1	44/3.9	3/7
C2_117	Spliceosome RNA helicase DDX39B	4344/22.5	22/183
C2_7011	Spliceosome RNA helicase DDX39B homolog	693/30.2	16/47
C2_991	Splicing factor 3B subunit 1	85/11.1	12/20
C2_17969	Splicing factor 3B subunit 3	167/20.6	7/12
C2_1156	Splicing factor U2AF 35 kDa subunit	38/8.4	2/2
C2_5025	Splicing factor, proline- and glutamine-rich	62/8.8	6/7

C2_121417	Sporulation-specific protein 15	41/11.3	3/5
C2_2583	SPRY domain-containing protein 4	53/3	1/2
C2_25237	Squamous cell carcinoma antigen recognized by T-cells 3	39/7.2	6/9
C2_39937	Src kinase-associated phosphoprotein 1	78/11.4	2/5
C2_1302	Staphylococcal nuclease domain-containing protein 1	956/27.8	30/68
C2_2582	StAR-related lipid transfer protein 7, mitochondrial	344/7.6	12/22
C2_6573	Stomatin-like protein 2	44/6.2	5/10
C2_82883	Stress-70 protein, mitochondrial	129/16.7	11/18
C2_8732	Stress-induced-phosphoprotein 1	111/19.3	11/21
C2_33905	Striatin	45/6.9	1/1
C2_15011	Stromal cell-derived factor 2-like protein 1	82/8.8	8/9
C2_4197	Stromal interaction molecule 1	45/2.4	2/2
C2_4258	Structural maintenance of chromosomes protein 1A	52/6.9	8/11
C2_3231	Structural maintenance of chromosomes protein 2	41/8	12/16
C2_4823	Structural maintenance of chromosomes protein 3	39/7.2	11/17
C2_1571	Succinate dehydrogenase [ubiquinone] flavoprotein subunit, mitochondrial	88/5.1	5/6
C2_1237	Succinate dehydrogenase [ubiquinone] iron-sulfur subunit, mitochondrial	52/4.9	3/3
C2_255	Succinyl-CoA ligase [ADP/GDP-forming] subunit alpha, mitochondrial	109/15.6	7/13
C2_1085	Sulfotransferase 1C1	71/12	4/5
C2_3986	Sulfotransferase 1C2	89/13.5	5/5
C2_7228	Sulfotransferase 6B1	87/6.6	6/8
C2_6124	SUMO-activating enzyme subunit 2	49/9.6	4/4
C2_368	Superoxide dismutase [Cu-Zn]	802/47.4	11/25
C2_1642	Superoxide dismutase 2, mitochondrial	119/19.1	5/5
C2_2004	Surfeit locus protein 4	141/9.3	7/9
C2_117981	Sushi domain-containing protein 1	34/18	2/2
C2_79043	SWI/SNF complex subunit SMARCC2	34/22.1	4/5
C2_19516	SWI/SNF-related matrix-associated actin-dependent regulator chromatin subfamily E member 1	42/6.3	3/3
C2_31339	Synaptic vesicle membrane protein VAT-1 homolog	82/6.5	2/3
C2_2785	Synaptobrevin homolog YKT6	61/5.2	4/7
C2_1838	Synaptogyrin-2	144/9.2	6/10
C2_8525	Synaptojanin-2-binding protein	41/7.3	8/10
C2_3925	Synaptophysin-like protein 1	136/6.5	9/13
C2_4210	Synaptopodin	39/3.7	4/15
C2_10514	Synaptopodin 2-like protein	47/3.8	7/18
C2_1809	Synaptosomal-associated protein 23	50/7.1	7/13
C2_1071	Syntaxin-12	36/4.8	3/5
C2_2320	Syntaxin-5	35/3	4/6
C2_3553	Syntaxin-7	47/5.6	4/7
C2_3302	Syntaxin-binding protein 2	36/6.4	3/3
C2_1047	Syntenin-1	32/6.7	6/7
C2_15378	Talin-1	105/11	5/8
C2_27605	Tapasin	47/17.6	3/5
C2_13909	Tapasin-related protein	59/4.4	5/6
C2_4568	TAR DNA-binding protein 43	96/7.2	5/8
C2_7259	TATA element modulatory factor	67/9.1	9/20
C2_13458	Tax1-binding protein 3	111/6	6/10
C2_8627	TBC domain-containing protein kinase-like protein	32/5.9	5/13
C2_75516	TBC1 domain family member 4	47/12.8	3/3
C2_2664	T-cell activation Rho GTPase-activating protein	43/7.3	10/20
C2_3143	TCF3 fusion partner homolog	36/2.2	1/1
C2_342	T-complex protein 1 subunit alpha	342/25.1	20/29

C2_2982	T-complex protein 1 subunit beta	401/22.7	15/25
C2_4858	T-complex protein 1 subunit delta	641/38.2	19/30
C2_298	T-complex protein 1 subunit epsilon	188/20.5	8/12
C2_1691	T-complex protein 1 subunit eta	297/20.7	14/18
C2_1250	T-complex protein 1 subunit gamma	840/38.3	22/40
C2_397	T-complex protein 1 subunit theta	410/23.5	15/24
C2_3533	T-complex protein 1 subunit zeta	318/13.6	5/10
C2_106651	T-complex protein 1 subunit zeta-2	44/40.4	2/2
C2_22620	Telomere-associated protein RIF1	31/6.5	5/10
C2_14486	Telomeric repeat-binding factor 2-interacting protein 1	56/9.1	6/10
C2_46605	Tensin-1	31/2.5	1/1
AM973187	Terminal uridylyltransferase 7	30/7.5	2/4
C2_8778	Testican-3	33/6.2	5/8
C2_7177	Testin	53/7.6	6/8
C2_16403	Tetranectin	31/5.1	2/2
C2_11322	Tetraspanin-8	140/9.1	5/14
C2_98024	Tetratricopeptide repeat protein 13	51/11.7	2/10
C2_652	Tetratricopeptide repeat protein 35	36/4.5	2/2
C2_15542	Thimet oligopeptidase	85/20.4	8/12
C2_1158	Thioredoxin	92/4.8	1/2
C2_4240	Thioredoxin domain-containing protein 12	41/4.1	2/2
C2_2180	Thioredoxin domain-containing protein 17	75/6.8	2/2
C2_5341	Thioredoxin domain-containing protein 5	59/3.1	4/6
C2_1093	Thioredoxin reductase 3	489/22.9	22/27
C2_3764	Thioredoxin-interacting protein	30/4.5	2/4
C2_1183	Thioredoxin-like protein 1	194/15.4	7/8
C2_13380	Thiosulfate sulfurtransferase/rhodanese-like domain-containing protein 1	205/22.4	8/13
C2_6564	THO complex subunit 4	75/11.6	7/14
C2_8209	THO complex subunit 6 homolog	33/2.6	1/1
C2_7672	Threonine synthase-like 2	38/6.3	4/5
C2_3045	Threonyl-tRNA synthetase, cytoplasmic	383/34.4	30/38
C2_21128	Thymidylate kinase	33/5.1	1/1
C2_1355	Thyrotroph embryonic factor	34/2.1	3/5
C2_3466	TIP41-like protein	38/10.2	5/5
C2_7405	Titin	58/6.2	8/10
C2_4043	Torsin-1A-interacting protein 2	49/8	6/8
C2_39632	Torsin-1B	40/7.4	3/3
C2_40379	Torsin-2A	46/7	2/2
C2_1998	TP53-regulating kinase	47/7.2	2/2
C2_1186	TP53RK-binding protein	107/9.4	4/4
C2_10331	Trafficking protein particle complex subunit 1	39/16.7	3/3
C2_44315	Trafficking protein particle complex subunit 11	52/6.3	1/1
C2_3017	Trafficking protein particle complex subunit 2	57/8.5	2/2
C2_8250	Trafficking protein particle complex subunit 8	69/5.6	5/7
C2_22469	TRAF-interacting protein	59/8.3	3/8
C2_18467	Trans-1,2-dihydrobenzene-1,2-diol dehydrogenase	48/15.3	6/7
C2_887	Trans-2,3-enoyl-CoA reductase	69/3.5	2/3
C2_564	Transaldolase	399/29.7	13/30
C2_529	Transcription elongation factor B polypeptide 1	122/15.4	6/6
C2_558	Transcription elongation factor B polypeptide 2	105/9.5	5/9
C2_52076	Transcription elongation regulator 1	51/14.1	4/16
C2_15171	Transcription factor 7-like 2	35/7.2	4/4
C2_670	Transcription factor BTF3	89/17.4	6/11
C2_14940	Transcription factor BTF3 homolog 4	93/9	2/3
C2_10954	Transcription factor E2F4	36/9.9	6/10

C2_22843	Transcription factor VBP	34/5.4	3/16
C2_10538	Transcriptional activator protein Pur-alpha	36/8.7	5/8
C2_1687	Transcriptional activator protein Pur-beta-B	55/6.5	5/5
C2_22296	Transcriptional regulator ATRX homolog	34/10	5/7
C2_3664	Transcriptional repressor CTCF	31/5.3	8/12
C2_7158	Transferrin	3916/45.9	39/155
C2_2328	Transforming acidic coiled-coil-containing protein 3	46/5.7	6/22
C2_131	Transforming protein RhoA	574/16.4	10/26
C2_10194	Transient receptor potential cation channel subfamily M member 7	47/6.1	4/4
C2_21917	Transient receptor potential cation channel subfamily V member 1	38/6.5	5/12
C2_15562	Transitional endoplasmic reticulum ATPase	1325/41.9	26/60
C2_824	Transketolase-like protein 2	2920/32.3	36/114
C2_8982	Translational activator GCN1	70/10.4	5/6
C2_15	Translationally-controlled tumor protein homolog	319/21.4	10/17
C2_23686	Translin	129/4.3	2/3
C2_28067	Translin-associated protein X	47/9.2	5/7
C2_5336	Translocation protein SEC63 homolog	58/7.8	4/6
C2_105	Translocon-associated protein subunit delta	345/16.1	7/18
C2_28031	Translocon-associated protein subunit gamma	239/7.5	2/5
C2_84253	Transmembrane 6 superfamily member 1	74/6.6	1/1
C2_3465	Transmembrane 9 superfamily member 2	40/4.5	4/4
C2_3796	Transmembrane 9 superfamily member 3	205/9.3	9/17
C2_9589	Transmembrane 9 superfamily member 4	187/8.1	10/10
C2_56013	Transmembrane and coiled-coil domains protein 1	167/14.2	5/9
C2_671	Transmembrane and immunoglobulin domain-containing protein 1	40/4.8	6/16
C2_8456	Transmembrane channel-like protein 7	43/5	6/8
C2_69	Transmembrane emp24 domain-containing protein 10	491/14	13/27
C2_12865	Transmembrane emp24 domain-containing protein 2	84/15.2	9/12
C2_2279	Transmembrane emp24 domain-containing protein 7	167/6.8	7/16
C2_1978	Transmembrane emp24 domain-containing protein 9	118/8.4	8/13
C2_12029	Transmembrane protease serine 3	36/5.8	5/5
C2_101217	Transmembrane protein 17	47/7	2/4
C2_30572	Transmembrane protein 173	65/12.9	2/2
C2_10320	Transmembrane protein 177	32/6.9	6/8
C2_11487	Transmembrane protein 203	48/5.3	5/11
C2_3271	Transmembrane protein 43	114/5.8	5/8
C2_25392	Transmembrane protein 60	47/1.9	1/12
C2_19623	Transportin-2	80/12.1	4/6
C2_22440	Transportin-3	41/4.9	4/4
C2_75383	Transthyretin	42/19.9	5/6
C2_10341	Tricarboxylate transport protein, mitochondrial	312/15.5	8/22
C2_2675	Trichohyalin	45/10.4	10/22
C2_3043	Trifunctional enzyme subunit alpha, mitochondrial	432/26.9	21/47
C2_2435	Trifunctional enzyme subunit beta, mitochondrial	107/6.2	4/5
C2_3068	Trinucleotide repeat-containing gene 18 protein	48/3.4	4/7
C2_11125	Triosephosphate isomerase	1357/51.1	22/58
C2_17605	Triosephosphate isomerase A	229/12.3	3/7
C2_52	Triosephosphate isomerase B	1391/45.9	23/60
C2_7955	Tripartite motif-containing protein 16	262/25	19/36
C2_10685	Tripartite motif-containing protein 29	171/20.6	15/29
C2_7124	Tripartite motif-containing protein 35	52/9.8	6/8
C2_108145	Tripartite motif-containing protein 62	77/14.5	2/3
C2_52881	Tripartite motif-containing protein 66	30/7.3	2/3
C2_27720	Tripeptidyl-peptidase 2	51/1.9	1/1
C2_10863	tRNA (guanine-N(7)-)-methyltransferase subunit WDR4	39/7.7	7/10

C2_37	Tropomyosin alpha-1 chain	142/16.3	11/15
C2_44653	Tropomyosin alpha-4 chain	164/12.6	8/11
C2_437	Tropomyosin beta chain	206/13.4	12/19
C2_121419	Trypsin-2	45/5.4	2/2
C2_18199	Tubulin alpha chain	2065/42.5	19/89
C2_101373	Tubulin alpha-1 chain	552/31.3	9/24
C2_110248	Tubulin alpha-1/alpha-2 chain	1766/42.9	14/74
C2_38179	Tubulin alpha-1A chain	1436/38.7	17/71
C2_7711	Tubulin alpha-1B chain	2579/40.1	21/101
C2_20113	Tubulin alpha-4A chain	1743/41.1	11/69
C2_905	Tubulin beta chain	4202/62.9	27/173
C2_90	Tubulin beta-1 chain	4743/55.3	30/183
C2_14202	Tubulin beta-2C chain	3960/45.4	25/148
FP334522	Tubulin gamma-2 chain	39/8.3	2/4
C2_36704	Tubulin polyglutamylase TTLL4	38/6.4	2/10
C2_26980	Tumor necrosis factor receptor superfamily member 11A	30/3.5	1/1
C2_1309	Tumor necrosis factor receptor superfamily member 1B	34/3.7	5/11
C2_2326	Tumor necrosis factor receptor type 1-associated DEATH domain protein	67/19.1	8/9
C2_625	Tumor necrosis factor, alpha-induced protein 8-like protein 2 B	93/12.5	6/6
C2_6149	Tumor protein D52	34/1.6	1/2
C2_20971	Tumor protein D54	165/18.2	7/11
C2_2309	Tumor suppressor candidate 3	36/6.7	4/10
C2_877	Tumor-associated calcium signal transducer 2	2142/32.9	21/86
C2_6143	Twinfilin-1	239/22.7	17/34
C2_5179	Twinfilin-2	37/9.6	6/7
C2_3196	Tyrosine-protein kinase JAK1	36/4.5	7/19
C2_28698	Tyrosine-protein kinase receptor Tie-1	54/7.7	3/3
C2_98117	Tyrosine-protein kinase transforming protein Fgr	1282/25.8	7/62
C2_40419	Tyrosine-protein phosphatase non-receptor type 7	32/10.4	3/5
C2_6304	Tyrosyl-tRNA synthetase, cytoplasmic	333/17.3	13/18
C2_28595	U1 small nuclear ribonucleoprotein 70 kDa	40/3.5	1/1
C2_6732	U1 small nuclear ribonucleoprotein A	63/6.5	3/5
C2_3206	U11/U12 small nuclear ribonucleoprotein 48 kDa protein	35/4.9	2/5
C2_5199	U2 small nuclear ribonucleoprotein B"	47/19.1	5/5
C2_2600	U2 snRNP-associated SURP motif-containing protein	37/5.3	6/7
C2_5077	U5 small nuclear ribonucleoprotein 200 kDa helicase	308/14.3	17/22
C2_28625	U5 small nuclear ribonucleoprotein 40 kDa protein	78/10.4	3/3
C2_3229	U6 snRNA-associated Sm-like protein LSm1	66/5.9	2/2
C2_28803	U6 snRNA-associated Sm-like protein LSm2	140/18.2	3/4
C2_26124	U6 snRNA-associated Sm-like protein LSm3	74/6.8	2/3
C2_7872	U6 snRNA-associated Sm-like protein LSm6	74/30.1	7/7
C2_516	Ubiquinol-cytochrome c reductase binding protein	48/21.2	4/4
C2_2118	Ubiquinol-cytochrome c reductase hinge protein	30/7.9	2/2
C2_12420	Ubiquinol-cytochrome c reductase, complex III subunit VII, 9.5kDa	108/35.4	7/9
C2_1166	Ubiquinol-cytochrome c reductase, complex III subunit X	120/22.9	4/7
C2_507	Ubiquinol-cytochrome c reductase, Rieske iron-sulfur polypeptide 1	75/16.5	8/10
C2_114174	Ubiquitin	1891/27.5	8/95
C2_1964	Ubiquitin carboxyl-terminal hydrolase 14	66/3.6	3/4
C2_11890	Ubiquitin carboxyl-terminal hydrolase 22	33/5.7	4/9
C2_18121	Ubiquitin carboxyl-terminal hydrolase 37	38/6.4	6/6
C2_20830	Ubiquitin carboxyl-terminal hydrolase 5	129/41.7	6/6
C2_18831	Ubiquitin carboxyl-terminal hydrolase 7	96/14.7	11/15
C2_66335	Ubiquitin carboxyl-terminal hydrolase 8	43/5.1	1/3
C2_5640	Ubiquitin carboxyl-terminal hydrolase isozyme L1	486/16.1	11/16

C2_660	Ubiquitin carboxyl-terminal hydrolase isozyme L3	127/8.7	4/4
C2_470	Ubiquitin fusion degradation protein 1 homolog	58/7.4	4/5
C2_155	Ubiquitin thioesterase OTUB1	170/11.6	3/3
C2_33738	Ubiquitin-40S ribosomal protein S27a	2191/42.1	10/100
C2_6783	Ubiquitin-60S ribosomal protein L40	1911/10	10/99
C2_5217	Ubiquitin-conjugating enzyme E2 29	65/3.6	6/7
C2_5227	Ubiquitin-conjugating enzyme E2 D2	125/16.7	5/8
C2_187	Ubiquitin-conjugating enzyme E2 D3	160/12.8	7/11
C2_17030	Ubiquitin-conjugating enzyme E2 N	461/30.5	12/29
C2_1249	Ubiquitin-conjugating enzyme E2 variant 1	94/9.1	4/4
C2_23677	Ubiquitin-conjugating enzyme E2 variant 1C	162/29.3	6/7
C2_112839	Ubiquitin-conjugating enzyme E2-18 kDa	54/17	4/8
C2_2467	Ubiquitin-fold modifier 1	52/7.7	2/4
C2_8231	Ubiquitin-like modifier-activating enzyme 1	2478/46.8	38/109
C2_39433	Ubiquitin-like modifier-activating enzyme 6	112/13.2	2/3
C2_6707	Ubiquitin-like modifier-activating enzyme ATG7	88/8.2	10/13
C2_19123	Ubiquitin-like protein 5	74/15.5	3/3
C2_5019	Ubiquitin-like-conjugating enzyme ATG3	32/3.3	4/4
C2_9398	Ubiquitin-protein ligase E3A	36/2.7	4/6
C2_7714	UDP-glucose 4-epimerase	121/20.2	11/20
C2_11633	UDP-glucose 6-dehydrogenase	86/4.4	3/12
C2_57655	UDP-glucose:glycoprotein glucosyltransferase 1	160/16.6	4/7
C2_110166	UDP-glucuronosyltransferase 1-4	56/9.5	2/2
C2_7856	UDP-glucuronosyltransferase 2A1	46/5.1	4/6
C2_3128	UDP-N-acetylglucosamine transporter	32/7.4	5/8
C2_9527	UDP-N-acetylglucosamine--peptide N-acetylglucosaminyltransferase 110 kDa subunit	42/0.7	1/1
C2_12651	UDP-N-acetylhexosamine pyrophosphorylase	259/22.1	12/12
C2_7433	UDP-N-acetylhexosamine pyrophosphorylase-like protein 1	152/9	6/7
C2_6090	UMP-CMP kinase	209/22	10/14
C2_21626	Uncharacterized family 31 glucosidase KIAA1161	41/11.3	4/5
C2_2371	Uncharacterized gene 87 protein	49/4.4	3/8
C2_3672	Uncharacterized oxidoreductase C663.06c	47/5.9	2/11
C2_21757	Uncharacterized protein C10orf47	43/9.4	4/4
C2_8312	Uncharacterized protein C18orf54	57/3.9	4/8
C2_1210	Uncharacterized protein C19orf52	43/2.3	2/3
C2_3059	Uncharacterized protein C2orf47 homolog, mitochondrial	45/10	8/12
C2_59282	Uncharacterized protein C4H3.07c	161/5.8	2/27
C2_13712	Uncharacterized protein C6orf106 homolog	37/6.9	7/13
C2_35069	Uncharacterized protein C6orf118	40/12.7	3/5
C2_90310	Uncharacterized protein C6orf203	55/13.4	3/8
C2_19837	Uncharacterized protein KIAA1522 homolog	40/4.6	3/5
C2_78488	Uncharacterized protein YJR142W	55/4.1	1/3
C2_17137	Unconventional prefoldin RPB5 interactor	46/5.4	2/8
C2_9334	UPF0160 protein MYG1, mitochondrial	33/4.6	3/4
C2_5877	UPF0462 protein C4orf33 homolog	43/6.6	2/3
C2_4195	UPF0489 protein C5orf22 homolog	40/4.1	3/4
C2_83574	UPF0505 protein C16orf62 homolog	39/6.4	2/3
C2_3292	UPF0534 protein C4orf43 homolog	35/5.8	3/3
C2_2220	UPF0556 protein C19orf10 homolog	104/11.2	4/7
C2_9002	UPF0684 protein C5orf30 homolog	40/4.7	5/9
C2_7447	UPF0687 protein C20orf27	35/1.5	2/2
C2_29534	Up-regulated during skeletal muscle growth protein 5	37/14	2/2
C2_15956	Up-regulator of cell proliferation	30/4	5/7
C2_6382	Upstream stimulatory factor 2	58/7.2	6/14

C2_2947	Uracil phosphoribosyltransferase homolog	42/4.6	9/14
C2_4863	UTP--glucose-1-phosphate uridylyltransferase	42/4.7	4/18
C2_26997	V(D)J recombination-activating protein 2	31/4.1	2/3
C2_860	Vacuolar ATPase assembly integral membrane protein VMA21	57/11.6	5/6
C2_9798	Vacuolar protein sorting-associated protein 13A	38/2.6	4/4
C2_12800	Vacuolar protein sorting-associated protein 13C	37/12.3	9/9
C2_17173	Vacuolar protein sorting-associated protein 28 homolog	42/7.1	2/2
C2_6316	Vacuolar protein sorting-associated protein 29	154/12.1	4/7
C2_5410	Vacuolar protein sorting-associated protein 33B	58/4.3	5/6
C2_3610	Vacuolar protein sorting-associated protein 35	363/19.8	16/20
C2_2867	Vacuolar protein sorting-associated protein 4B	141/7.1	6/10
C2_9965	Vacuolar protein sorting-associated protein VTA1 homolog	43/8.9	4/6
C2_6246	Vacuolar-sorting protein SNF8	69/7	4/6
C2_5432	Valyl-tRNA synthetase	498/17	12/26
C2_59269	Vang-like protein 2	42/5.5	1/2
C2_770	Vesicle-associated membrane protein 3	60/3.9	3/3
C2_8727	Vesicle-associated membrane protein 8	34/9.6	6/8
C2_7834	Vesicle-associated membrane protein-associated protein A	39/3.9	1/1
C2_3642	Vesicle-associated membrane protein-associated protein B	73/14.1	6/12
C2_4322	Vesicle-associated membrane protein-associated protein B/C	317/12.5	11/35
C2_8296	Vesicle-fusing ATPase	100/24	11/15
C2_5259	Vesicle-trafficking protein SEC22b-B	74/6.9	3/7
C2_1304	Vigilin	161/11.5	18/34
C2_37611	Villin-1	6150/59.7	22/231
C2_3381	Vimentin	641/6.9	6/47
C2_9594	Vinculin	40/8.9	6/7
C2_38345	Vitellogenin-1	42/6	3/6
C2_318	Voltage-dependent anion-selective channel protein 1	617/51.7	20/32
C2_661	Voltage-dependent anion-selective channel protein 2	1033/28.5	15/38
C2_14015	Voltage-gated potassium channel subunit beta-1	48/6.6	4/5
C2_2397	von Willebrand factor A domain-containing protein 5A	511/13.8	12/33
C2_1031	V-type proton ATPase 16 kDa proteolipid subunit	59/7.5	2/2
C2_3806	V-type proton ATPase catalytic subunit A	99/13.8	9/11
C2_1084	V-type proton ATPase subunit B	268/19.6	14/16
C2_5241	V-type proton ATPase subunit E 1	51/6	2/2
C2_18009	V-type proton ATPase subunit G 1	93/11.5	4/4
C2_275	V-type proton ATPase subunit H	56/10.4	6/11
C2_29757	WAS/WASL-interacting protein family member 2	43/11.3	6/7
C2_7457	WASH complex subunit FAM21	40/6.7	2/3
C2_11192	WD and tetratricopeptide repeats protein 1	38/5.5	5/7
C2_1420	WD repeat-containing protein 1	2220/49.8	33/110
C2_97790	WD repeat-containing protein 26	63/3.5	2/5
C2_65738	WD repeat-containing protein 36	61/4.6	1/9
C2_1995	WD repeat-containing protein 61	42/8.8	4/4
C2_16653	WD repeat-containing protein 85	30/4.4	2/3
C2_66255	Williams-Beuren syndrome chromosomal region 16 protein homolog	35/4.3	1/1
C2_81255	Wings apart-like protein homolog	48/8.2	1/3
C2_4369	Xaa-Pro aminopeptidase 1	67/12.9	9/11
C2_1116	Xaa-Pro dipeptidase	89/15.1	8/11
C2_17251	Xin actin-binding repeat-containing protein 2	62/2.8	3/6
C2_6902	X-ray repair cross-complementing protein 5	73/5.9	6/10
C2_10177	Xylose isomerase	30/7.9	5/8
C2_2803	YEATS domain-containing protein 4	37/9.6	4/10
C2_853	Zinc finger matrin-type protein 2	40/5.9	12/21
C2_37106	Zinc finger protein 112	41/2.6	1/6

C2_36552	Zinc finger protein 227	37/2.5	1/2
C2_15025	Zinc finger protein 236	40/5.3	6/9
C2_1528	Zinc finger protein 502	38/6.8	10/13
C2_18520	Zinc finger protein 544	41/7.1	5/13
C2_29736	Zinc finger protein 57	47/2.5	1/2
C2_12406	Zinc finger protein 609	33/7.9	5/8
C2_1743	Zinc finger protein 675	30/7.1	4/6
C2_201	Zinc finger protein 706	38/8.1	4/11
C2_35539	Zinc finger protein RFP	127/14.3	8/15
