# Supplementary material for: Skin Mucus of Gilthead Sea Bream (Sparus aurata L.). Protein Mapping and Regulation in Chronically Stressed Fish
Source: Front Physiol. 2017 Feb 1;8:34. doi: 10.3389/fphys.2017.00034 (PMC5288811; doi:10.3389/fphys.2017.00034)

Figure S1. Principal component analysis of image processing of 2-DE of skin mucus proteins from individual samples of CTRL (blue) and M-ST (yellow) groups.

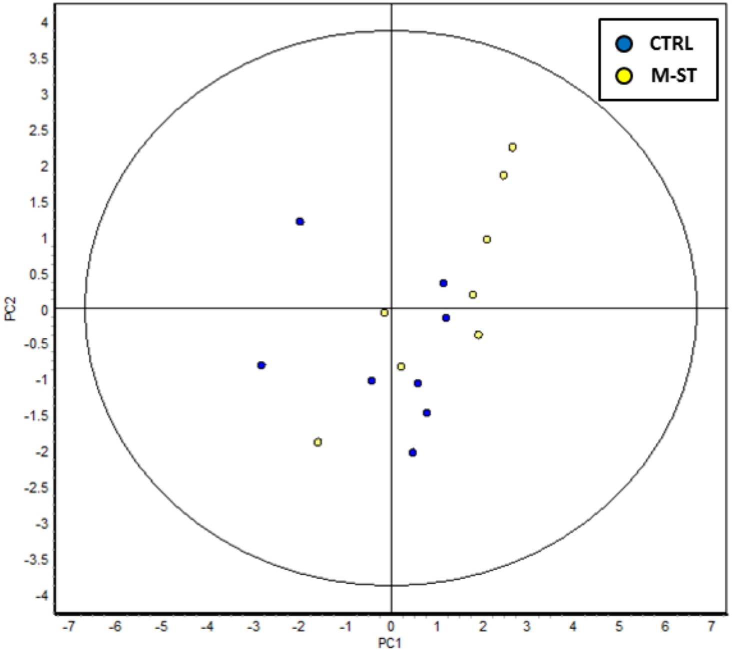

Supplement: Supplementary file 2 [file Image1.pdf]
